# Expanding Meroterpenoid Chemical Space Via Intermolecular Trapping of Cationic Cyclization Intermediates

**DOI:** 10.1021/jacsau.5c00492

**Published:** 2025-06-27

**Authors:** Ivan Cornu, Daniel Häussinger, Alessandro Prescimone, Konrad Tiefenbacher

**Affiliations:** † Department of Chemistry, 27209 University of Basel, Mattenstrasse 22, 4058 Basel, Switzerland; ‡ Department of Biosystems Science and Engineering, ETH Zurich, Klingelbergstrasse 48, 4056 Basel, Switzerland

**Keywords:** Resorcin[4]arene catalysis, carbocation trapping, meroterpenoid synthesis, supramolecular catalysis

## Abstract

Meroterpenoids are a subclass of terpenes that consist of a nonterpene part, that is, in many cases, an aryl residue, linked to a terpene component. Their biosynthesis involves the attachment of the aryl group to the tail end of the acyclic terpene part, followed by terpene cyclization. Here we explore an alternative method for accessing meroterpenoids. The terpene cyclization is performed in the presence of arenes that trap key cationic intermediates in an intermolecular fashion. Our study demonstrates the feasibility of this approach that, to our knowledge, has not been reported before. It enables direct access to the novel meroterpenoid chemical space.

## Graphical abstract

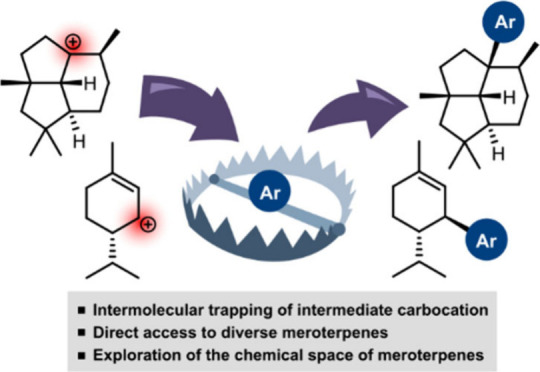

Terpenes represent the largest class of natural products, distinguished by their highly diverse and complex structural architectures.^1–5^ Remarkably, this diversity arises from a limited set of highly conserved precursor molecules such as farnesyl pyrophosphate (FPP, Figure 1a). One diversification strategy employed by nature is the incorporation of nonterpene moieties, resulting in the formation of so-called meroterpenoids.^6–8^ Their biosynthesis involves the attachment of the nonterpene part, which is in many cases an aryl residue, to the tail end of the acyclic terpene part. Only afterward, terpene cyclization, in a head-to-tail fashion, is initiated to form the final cyclized meroterpenoid (Figure 1a). The cationic intermediate formed during the cyclization is thus quenched by the aryl part in an *intra*molecular fashion. *Inter*molecular trapping would (i) enable more direct access to meroterpenes and (ii) enable access to unexplored chemical space. However, there are no examples of natural terpene cyclizations that are terminated by an *inter*molecular trapping with an aryl group to the best of our knowledge. Such a methodology would further help expand the non-natural terpene space.^9–16^ Even in the broader context of chemical terpene-like cyclizations, intermolecular trapping is rarely observed. Such examples include the linkage of the menthane framework with aryl groups to form menthane meroterpenes without a preceding cyclization reaction.^17,18^ Furthermore, Prins-type cyclizations were combined with aryl trapping.^19–24^

**Figure 1:**
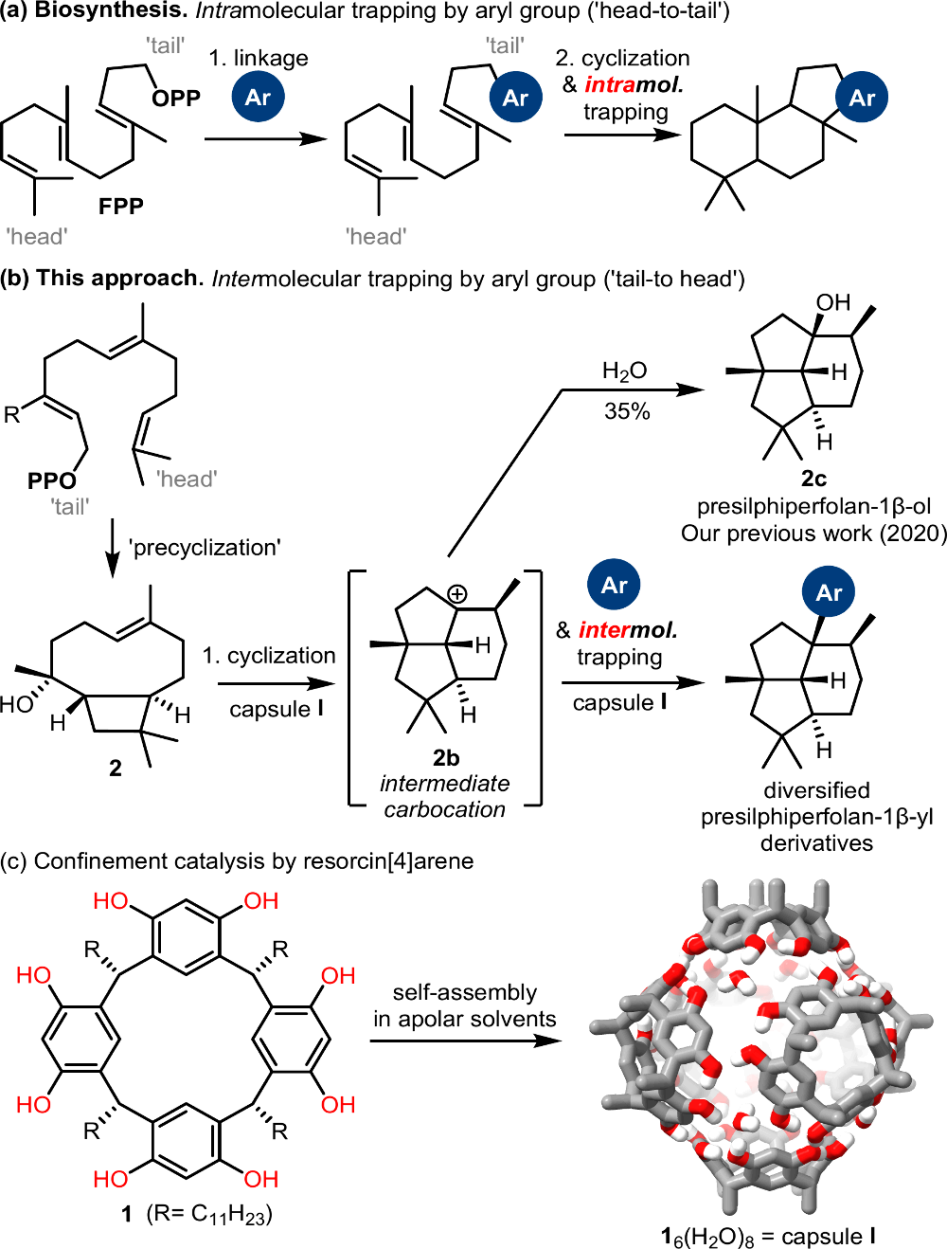
(a) Typical biosynthetic pathway of meroterpene; (b) cyclization of caryophyllene alcohol **2** in capsule **I** in the presence of an external nucleophile; (c) self-assembly of resorcin[4]­arene into a hexameric capsule.

Our group reported the four-step synthesis of presilphiperfolan-1β-ol **2c** by confinement catalysis,^25,26^ utilizing the resorcin[4]­arene capsule **I** (Figure 1c).^27–32^ The modification of the precursor also permitted *intra*molecular trapping of the carbocation,^33^ distantly related to meroterpene biosynthesis. The Chappell group also reported *intra*molecular trapping of a non-natural arene-containing substrate, albeit via enzymatic catalysis.^34^ In this work, we explore the possibility of trapping the tail-to-head^35^ cyclization with an aryl residue in an *inter*molecular fashion.

While intermolecular Friedel–Crafts alkylation and acylation have been reported in the resorcin[4]­arene capsule,^36,37^ to our knowledge, the combination with terpene cyclizations has been observed in neither biosynthesis nor a chemical laboratory.

We started the investigation with the sesquiterpene caryophyllene alcohol **2**, which was the direct precursor for our capsule-catalyzed cyclization to presilphiperfolan-1β-ol **2c** via the intermediary carbocation **2b** (Figure 1b).^25^ A first nucleophile screening led to a hit for the reaction with 2-methylfuran **4**, producing the furan derivative **10** (Table 1, see Supporting Information chapter S2 for details). The reaction conditions were optimized regarding temperature, equivalents of nucleophile, and amount of HCl cocatalyst (see chapter S3). For further reactions, the following optimized conditions were used; 10 mol % capsule **I**, 4 mol % HCl, and 10 equiv of nucleophile at 60 °C. Under these conditions, an isolated yield of 29% of **10** (GC yield of 55%) was obtained. Additional product (18% **10**) was collected in separate fractions as a mixture with its C19 epimer, named **10b** (ratio of **10**/**10b** approximately 1.8/1). A range of additional nucleophiles, 3-methylfuran **5**, 2,3-dimethylfuran **6**, 1,3-dimethoxybenzene **7**, resorcinol **8**, and *N*-methylpyrrole **9**, were tested. Besides 2-methylfuran **4**, 2,3-dimethylfuran **6** also delivered the desired product. To our knowledge, these examples represent the first meroterpenoids formed by an intermolecular quench of a terpene cyclization.

**Table 1:**
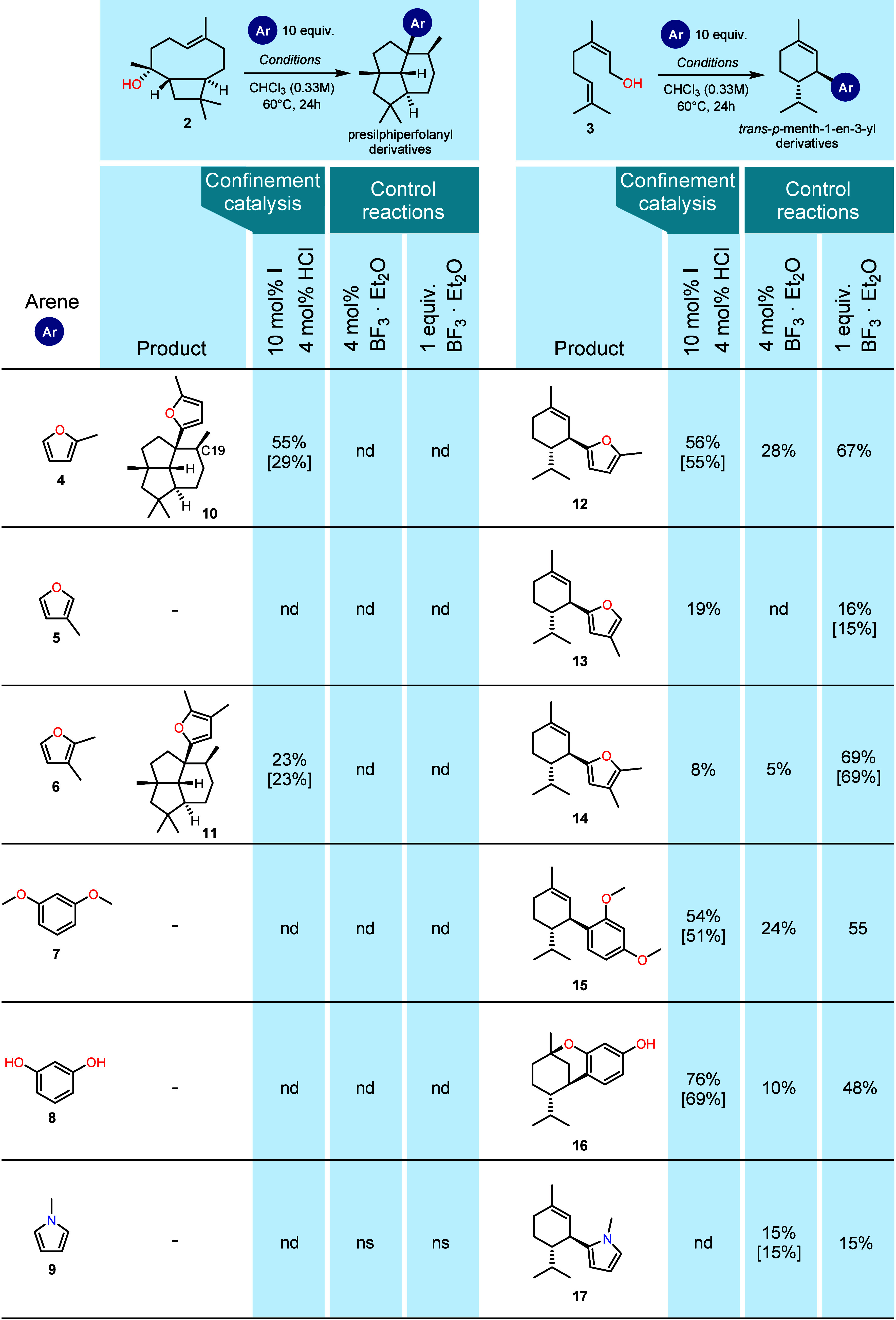
Scope of the Substrates and Nucleophiles in the Cyclization Reactions^a^

a Control reactions with boron trifluoride diethyl etherate in catalytic and stoichiometric amounts are shown. GC yields were corrected using calculated relative response factors.^42^ Isolated yields are indicated in [brackets]. nd = not detected, ns = not selective. Reaction conditions: 0.033M substrate in CHCl_3_ at 60°C; catalyst details and amounts; see table.

The closely related 3-methylfuran **5** did not lead to a detectable product formation. Unsubstituted furan, which is less nucleophilic,^38,39^ only led to very low yields of product mixtures which did not allow for their isolation (see chapter S9.1). Thus, the 2-methyl substituent on the furan might be required to stabilize the dearomatized intermediate (see chapter S6.3). Related to this observation, 2-methylthiophene also displayed low reactivity (see chapter S9.2), most likely again owing to its lower nucleophilicity. Furthermore, none of the six-membered aromatic nucleophiles explored (such as 1,3-dimethoxybenzene **7** and resorcinol **8**) yielded products in isolatable yield. It seems that intermediate carbocation **2b** (Figure 1b) is sterically too encumbered for an attack by benzene derivatives. *N*-methylpyrrole **9**, on the other hand, displayed poor selectivity, and no defined product was isolable.

We then investigated the cyclization of nerol **3**, a commercially available monoterpene, that shows tail-to-head cyclization reactivity also with Lewis and Brønsted acids.^40^ Its cyclization inside capsule **I** has been previously studied by our group and led to the formation of a mixture of products.^41^ In this work, the reaction demonstrated a higher selectivity. A *trans*-substituted *p*-menth-1-en-3-yl carbon skeleton was formed as the major product. To the best of our knowledge, this is the first time that the formation of this structure has been observed from the cyclization of a linear precursor. In contrast to substrate **2**, nerol **3** afforded products for all three furans tested (**12**-**14**, Table 1). Moreover, it was compatible with benzene derivatives (products **15**-**16**). In the case of resorcinol **8**, a cyclic ether was formed, most likely owing to the protonation of the endocyclic double bond after the formation of the *p*-menth-1-en-3-yl scaffold. With the idea of extending the scope to a less nucleophilic benzene derivative, *m*-xylene was explored as a solvent. However, the reaction was unselective and led to multiple product peaks on the GC trace (see chapter S9.3). Nitrogen heterocycles such as pyrrole **9** led to poor results with resorcin[4]­arene-catalysis. Despite their high nucleophilicity, they may act as a base and neutralize the required cocatalyst HCl. Thus, the exploration of amine nucleophiles has not been further pursued.

For all reactions, the main product was isolated from the reaction mixture using either silica gel flash chromatography or recycling GPC (see chapter S1.1 for details). The product structures were elucidated via ^1^H, ^13^C, DEPT135, COSY, NOESY, HSQC, and HMBC NMR measurement. In two cases, for products **10** and **19** (Figure 2), additionally, a 1,1-ADEQUAT NMR experiment was required to assign the structure since the ^1^H NMR spectra showed significant signal overlap. Moreover, ^13^C chemical shifts were calculated for all the structures and were used to exclude other potential isomers (see chapter S14).

**Figure 2:**
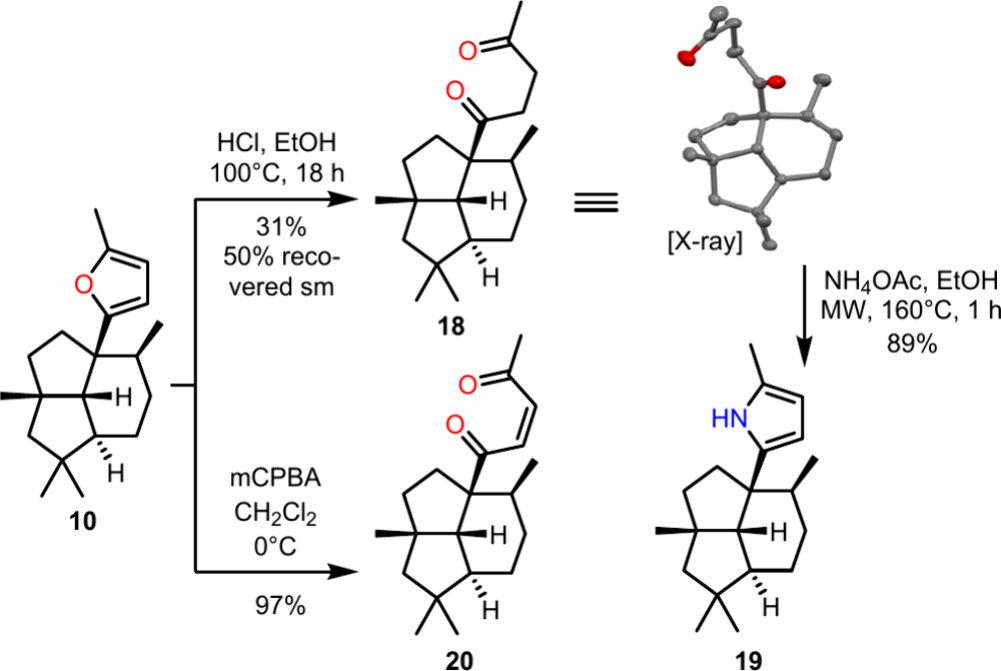
Derivatization options for furan **10**.

Next, we sought to determine whether the observed reactions could also proceed outside the supramolecular capsule. In our previous work^25^ on the cyclization of caryophyllene alcohol **2**, we screened 12 different reaction conditions but did not identify any Lewis or Brønsted acid capable of promoting the cyclization to the presilphiperfolane framework. Thus, a screening (see chapter S8) of different acids was performed with the alternative substrate nerol **3** (see also Table 1, right part). Only boron trifluoride diethyl etherate, in either catalytic or stoichiometric amounts, was identified as a suitable promoter for the cyclization/intermolecular quench reaction of **3** and thus was used as promoter instead of capsule **I** and HCl in control reactions for all entries. For the substrate nerol **3**, in some cases, even higher yields were obtained using boron trifluoride diethyl etherate in catalytic or stoichiometric amounts. Lewis acid-catalyzed cyclization in solution is thus an alternative to produce monoterpene-based meroterpenoids via this strategy. In fact, it was the only successful reaction condition to produce compound **17**. Interestingly, when the nucleophile was omitted from the boron trifluoride reactions, no defined product was formed, and the GC trace remained largely silent. Instead, a black suspension was obtained. Based on this observation, it seems that the nucleophile quench decreases the undesirable formation of undefined oligomeric products.

However, for the caryophyllene alcohol substrate **2**, all control experiments with either catalytic or stoichiometric amounts of boron trifluoride diethyl etherate failed to deliver any traces of product, confirming our earlier finding^25^ that the presilphiperfolane framework was not accessible from **2** without the capsule catalyst. However, the question remained if, after successful capsule-catalyzed cyclization to the presilphiperfolanyl cation **2b** (Figure 1b), the electrophilic aromatic substitution with aryl residues is possible without the capsule. Thus, two additional control experiments were run. (1) Subjecting presilphiperfolan-1β-ol **2c** to HCl (4 mol %) and 2-methylfuran **4** (10 equiv) did not lead to the formation of the furan derivative **10**. In the presence of resorcin[4]­arene **1** and HCl, the derivative **10** was formed in 65% yield. (2) Subjecting presilphiperfolan-1β-ol **2c** to the control reaction conditions of Table 1 (boron trifluoride diethyl etherate in catalytic or stoichiometric amount) also did not lead to the formation of the derivative **10**. Thus, both steps of the caryophyllene substrate conversion, cyclization, and carbocation trapping require the presence of the capsule. It seems that the capsule is required to stabilize the intermediate carbocation as in its absence alkene formation via elimination was observed.

After having explored the scope and limitations of intermolecular meroterpenoid formation, we tried to expand the chemical space of the meroterpenoids formed. This seemed especially of interest for the presilphiperfolane meroterpenoids, as the aryl nucleophile scope turned out to be limited to 2-substituted furans. Thus, we explored the ring opening of the furan in compound **10** (Figure 2). Standard reaction conditions^43^ (HCl, EtOH, 100 °C) for the ring-opening step afforded 31% diketone **18**. Although incomplete conversion of the starting material was observed, it could be recovered easily (50% recovered sm). The crystal structure of diketone **18** was obtained and unambiguously confirmed the assignment of the presilphiperfolanyl carbon skeleton. Acid-catalyzed Paal-Knorr formation^44^ of pyrrole **19** was unsuccessful and led to the formation of the parent furan **10**. Moreover, it was found that traces of formed pyrrole **19** decomposed under acidic conditions. Thus, neutral conditions using ammonium acetate were explored,^45^ making the pyrrole derivative **19** that was inaccessible directly, attainable via this two-step procedure. Alternatively, furan **10** can be opened oxidatively with mCPBA^46^ to the unsaturated diketone **20** in high yield, enabling additional derivatization options.

The study successfully demonstrates a novel approach to meroterpenoids by intermolecular trapping of carbocations formed during terpene cyclizations. It enables direct access to novel meroterpenoid chemical space. For the caryophyllene alcohol substrate **2**, the resorcinarene capsule **I** plays a crucial role in catalyzing the initial terpene cyclization as well as the final intermolecular electrophilic aromatic substitution. These results further illustrate the benefits of conducting reactions within supramolecular containers.^47–64^ For nerol **3**, however, the Lewis acid boron trifluoride also turned out to be a suitable catalyst and/or promoter. The reaction is sensitive to the structure of the nucleophile, with 2-substituted furans showing the best results for the sterically encumbered cationic intermediate formed from the caryophyllene alcohol. Nerol displays a much broader arene scope, reacting with furans, benzene derivatives, and pyrrole derivatives. Chemical derivatization of the furan-containing meroterpenoids allows for the further expansion of the chemical space. The study demonstrates the potential of intermolecular trapping as a valuable tool in the synthesis of structurally novel meroterpenes, providing a foundation for future exploration and development.

## Supplementary Material



## Data Availability

Raw data for the table and figures have been deposited at https://zenodo.org/records/15754845.

## Notes

General information, additional reaction screenings, reaction optimization, experimental procedures, characterization data of compounds, crystallographic details, ^13^C NMR shift calculations and NMR spectra (PDF)

## Notes

CCDC 2441112 contains the supplementary crystallographic data for this paper. These data can be obtained free of charge via www.ccdc.cam.ac.uk/data_request/cif.
