# Supplementary material for: Expanding Meroterpenoid Chemical Space Via Intermolecular Trapping of Cationic Cyclization Intermediates
Source: JACS Au. 2025 Jun 27;5(7):3021–6. doi: 10.1021/jacsau.5c00492 (PMC12308376; doi:10.1021/jacsau.5c00492)

# Supporting Information for Publication.

# Expanding Meroterpenoid Chemical Space via Intermolecular Trapping of Cationic Cyclization Intermediates

*Ivan Cornu,[1] Daniel Häussinger,[1] Alessandro Prescimone,[1] Konrad Tiefenbacher[1,2]*

*[1] Department of Chemistry, University of Basel, Mattenstrasse 22, 4058 Basel, Switzerland*
*[2] Department of Biosystems Science and Engineering, ETH Zurich, Klingelbergstrasse 48, 4056 Basel, Switzerland*

*Email: konrad.tiefenbacher@unibas.ch*

# Table of Contents


S1 General information ....4
S1.1 Experimental ....4
S1.2 Gas chromatography analyses ....6
S1.3 Retention times ....7
S2 First nucleophile screening ....8
S3 Optimization of the cyclization reaction conditions ....9
S4 Synthesis of catalyst and substrates ....10
S4.1 Resorcin[4]arene 1 ....10
S4.2 Presilphiperfolan-1β-ol 2c ....10
S4.3 Kobusone s2 ....10
S4.4 Reduced kobusone s3 ....11
S4.5 Caryophyllene alcohol 2 ....11
S5 General procedures for screening scale reactions ....12
S5.1 General procedure A: Using resorcin[4]arene as catalyst ....12
S5.2 General procedure B: Using $BF_3 \cdot Et_2O$ as catalyst ....13
S6 General procedure for large scale reactions ....13
S6.1 General procedure C: Using resorcin[4]arene as catalyst ....13
S6.2 General procedure D: Using $BF_3 \cdot Et_2O$ as catalyst ....14
S6.3 2-(presilphiperfolanyl)-5-methylfuran 10 ....14
S6.4 2-(presilphiperfolanyl)-5-methylfuran epimer 10b ....15
S6.5 2-(presilphiperfolanyl)-4,5-dimethylfuran 11 ....16
S6.6 2-(*trans*-*p*-menth-1-en-3-yl)-5-methylfuran 12 ....17
S6.7 2-(*trans*-*p*-menth-1-en-3-yl)-4-methylfuran 13 ....17
S6.8 2-(*trans*-*p*-menth-1-en-3-yl)-4,5-dimethylfuran 14 ....18
S6.9 2-(*trans*-*p*-menth-1-en-3-yl)-1,5-dimethoxybenzene 15 ....19
S6.10 Cyclic ether 16 ....19
S6.11 2-(*trans*-*p*-menth-1-en-3-yl)-1-methylpyrrole 17 ....20
S7 Synthesis of 2-(presilphiperfolanyl)-5-methylfuran 10 derivatives ....21
S7.1 1-presilphiperfolanylpentane-1,4-dione 18 ....21
S7.2 2-presilphiperfolanyl-1-methylpyrrole 19 ....22
S7.3 (2Z)-1-presilphiperfolanylpent-2-ene-1,4-dione 20 ....22
S8 Screening of control reactions ....24
S9 Cyclization with other nucleophiles ....25
S9.1 Furan ....25
S9.2 2-methylthiophene ....25

S9.3 *m*-xylene ..... 26
S9.4 Other unseccessulf nucleophiles ..... 26
S10 Cyclization with other electrophiles ..... 26
S11 Nucleophile encapsulation study ..... 27
S12 References ..... 28
S13 Crystallographic details for compound 18 ..... 29
S14 $^{13}$C chemical shift calculations ..... 36
S15 NMR spectra ..... 37
S15.1 2-presilphiperfolanyl-5-methylfuran 10 ..... 37
S15.2 2-presilphiperfolanyl-5-methylfuran epimer 10b ..... 41
S15.3 2-presilphiperfolanyl-4,5-dimethylfuran 11 ..... 44
S15.4 2-(*trans*-*p*-menth-1-en-3-yl)-5-methylfuran 12 ..... 47
S15.5 2-(*trans*-*p*-menth-1-en-3-yl)-4-methylfuran 13 ..... 50
S15.6 2-(*trans*-*p*-menth-1-en-3-yl)-4,5-dimethylfuran 14 ..... 53
S15.7 2-(*trans*-*p*-menth-1-en-3-yl)-1,5-dimethoxybenzene 15 ..... 56
S15.8 Cyclic ether 16 ..... 59
S15.9 2-(*trans*-*p*-menth-1-en-3-yl)-1-methylpyrrole 17 ..... 62
S15.10 1-presilphiperfolanylpentane-1,4-dione 18 ..... 65
S15.11 2-presilphiperfolanyl-1-methylpyrrole 19 ..... 68
S15.12 (2Z)-1-presilphiperfolanylpent-2-ene-1,4-dione 20 ..... 72

# S1 General information

## S1.1 Experimental

Reactions were carried out under air unless otherwise indicated.

Analytical thin-layer chromatography (TLC) was performed on Merck silica gel 60 $F_{254}$ aluminium-backed plates unless otherwise stated.

All NMR experiments were performed on a Bruker Avance Neo and a Bruker Avance III HD NMR spectrometer operating at 500 MHz and 600 MHz proton frequency, respectively. The instruments were equipped with a direct observe 5-mm BBFO smart probe (500 MHz) or a five-channel cryogenic 5 mm QCI probe (600 MHz). All probes were equipped with actively shielded z-gradients (10 A). The measurements were performed at 298 K. Chemical shifts of $^{1}$H NMR and $^{13}$C NMR are given in ppm. The following solvent residual signals of the deuterated solvents were used as reference: $CDCl_3$: 7.26 ppm ($\delta^{1}$H), 77.16 ppm ($\delta^{13}$C). Coupling constants (*J*) are reported in Hertz (Hz). Standard abbreviations indicating multiplicity were used as follows: s (singlet), d (doublet), t (triplet), q (quartet), p (quintet), h (sextet), dd (doublet of doublets), m (multiplet).

Infrared spectra were recorded on an Alpha-II FT-IR spectrometer from Bruker, equipped with a Platinum ATR probe. Standard abbreviations for peak description were used as follow: s (strong), m (medium), w (weak), br (broad).

High-resolution mass spectra were obtained on a Thermo Scientific LTQ-FT Ultra via electrospray ionization (ESI) or a Finnigan MAT 8200 (EI) (ESI source parameters for positive polarity mode were: spray voltage, 4.0 kV; capillary temperature, 275 °C; capillary voltage, 48 V; and tube lens, –120 V).

Optical rotations were measured on an Anton Paar MCP 100 Circular Polarimeter operating on the sodium D-line (589 nm).

Ozonolysis was carried out using a BMT Ozone Generator 802N.

Microwave syntheses were carried out in a Monowave 400 from Anton Paar.

Medium Pressure Liquid Chromatography (MPLC) was carried out with RediSep® Silica Gel Disposable Flash $SiO_2$ columns (particle size 40-60 µm) performed on a CombiFlash NextGen version 5.1.11 with a fraction collector version 00.92.00, detector version 7, and a pump version 2.81 from Teledyne.

For manual flash chromatography, SilicaFlash P60 (40-63 µm) from Silicycle was used.

Recycling gel permeation chromatography (GPC) purification were performed on a LaboACE LC-5060 Plus II from Japan Analytical Industry Co. Ltd equipped with a JAIGEL-2HR Plus and JAIGEL-2.5HR Plus column in series. Chloroform stabilized with 0.75% ethanol was used as eluent.

Transfer of liquids with a volume ranging from 1 to 10 µL or from 10 to 100 µL was performed with a Microman M1 pipette (Gilson) equipped with 10 µL or 100 µL pipette tips, respectively.

## Sources of chemicals:

| Chemical | Cat. No. | Supplier | Comment |
|---|---|---|---|
| (−)-caryophyllene oxide 95% | W509647 | Sigma-Aldrich | Stored at -20°C. |
| 1,3-dimethoxybenzene ≥98% | 126306 | Sigma-Aldrich | |
| 2,3-dimethylfuran 99% | 428469 | Sigma-Aldrich | |
| 2-methylfuran >97% | 85420 | Fluka | It was distilled from KOH.[1] |
| 3-methylfuran | F005088 | Fluorochem | |
| acetic acid ≥99.8% | 33209 | Honeywell | |
| aluminium oxide basic, Brockmann I | 463350010 | ThermoScientific | |
| ammonium acetate 98+% | 218365000 | Acros Organics | |
| boron trifluoride etherate | 175501 | Sigma-Aldrich | |
| chloroform stabilized with amylene (for cyclization) | C2432 | Sigma-Aldrich | It was passed through a plug of basic aluminium oxide and kept over molecular sieves, in a dark place. |
| chloroform stabilized with ethanol (for recycling GPC) | 7386 | J. T. Baker | |
| copper (II) acetate monohydrate 98+% | 203121000 | Acros Organics | |
| deuterated chloroform 99.8 % D | D007HAG | Eurisotop | |
| dichloromethane (HPLC grade) | 34856 | Honeywell | |
| diethyl ether stabilized with butylated hydroxytoluene (BHT) | 00052802A5BS | Biosolve | It was carefully distilled in a dedicated rotary evaporator to remove the BHT. |
| dimethylsulfoxide 99.7+% extra dry | 348441000 | ThermoScientific | |
| ethanol (HPLC grade) | 111727 | Merck | |
| furan ≥99% | 185922 | Sigma-Aldrich | Stored at 4°C. |
| hydrochloric acid ≥37% aqueous | 30721 | Honeywell | |
| mCPBA ≤77% | 273031 | Sigma-Aldrich | The carboxylic acid impurity was removed by extraction with a phosphate buffer pH 7.5 (0.2 mol/L).[1] Stored at 4°C. |
| methylmagnesium bromide 3.0 mol/L in $Et_2O$ | 189898 | Sigma-Aldrich | Stored at 4°C. |
| m-xylene 99+% | 180862500 | ThermoScientific | |
| nerol 97% | 268909 | Sigma-Aldrich | Stored at 4°C. |
| *n*-dodecane 99% | 117590250 | Acros Organics | |
| N-methylpyrrole 99% | M78801 | Sigma-Aldrich | It was distilled from KOH.[1] |
| pentane (HPLC grade) | 83632.320 | VWR Chemicals | |
| phenol red | P3532 | Sigma-Aldrich | |
| potassium hydroxide 85% | 232550010 | ThermoScientific | |
| resorcinol 99% | 307521 | Sigma-Aldrich | |
| sodium chloride techn. | 27788.297 | VWR Chemicals | |
| sulphuric acid 95% | 20685.295 | VWR Chemicals | |
| triethylamine | 471283 | Sigma-Aldrich | |
| zinc dust ≥98% | 209988 | Sigma-Aldrich | |

## S1.2 Gas chromatography analyses

GC analyses were carried out on a Shimadzu GC-2010 Plus instrument equipped with a flame ionization detector (FID) set at 340°C and an Rtx-5 capillary column (length = 30 m, crossbonded diphenyl dimethyl polysiloxane). Hydrogen was used as the carrier gas at a constant linear velocity of 70 cm/s The injector was set to 250°C with a split ratio of 1:20. The two following temperature programs were used:

Temperature program A: 70 °C for 1 min, 25 °C/min to 330 °C, and 330 °C for 4 min.

Temperature program B: 150 °C for 10.5 min, 25 °C/min to 200 °C, and 200 °C for 1 min.

*n*-Dodecane was used as internal standard for the determination of GC yield in the small scale screening reactions. It was omitted for the large scale reaction to avoid purification issues. Relative response factors employing the effective carbon number (ECN)[2] were used to correct the obtained GC yield. They were calculate as follow:

$$RRF(X) = \frac{ECN(X)}{ECN(IS)}$$

with

RRF(X) : relative response factor of compound X
ECN(X) : effective carbon number of compound X
ECN(IS) : effective carbon number of the internal standard (*n*-dodecane=12)

The corrected GC yields were then calculated as follow;

$$Yield(X) = \frac{\frac{\frac{A(X)_t}{RFF(X)}}{A(IS)_t}}{\frac{\frac{A(S)_0}{RFF(S)}}{A(IS)_0}} \cdot 100 = \frac{RFF(S)}{RFF(X)} \frac{\frac{A(X)_t}{A(IS)_t}}{\frac{A(S)_0}{A(IS)_0}} \cdot 100$$

with

A : area
X : product X
IS : internal standard (*n*-dodecane)
S : substrate
RRF : relative response factor
t : at time t
0 : at time 0

Retention indexes (RI) were calculated after characterizing the GC column with a mixture of *n*-alkanes. The number of carbons times one hundred in each *n*-alkanes was reported as a function of the retention time (in minutes) of the *n*-alkane. The data was fitted with a quadratic model and was valid between 1 to 10 min;

$$RI = 11.3\ x^2 + 75.6\,x + 732$$

with

RI : retention index
x : retention time (min)

## S1.3 Retention times

On temperature program A

| Compound | | Retention time (min) | Retention index (-) |
|---|---|---|---|
| caryophyllene oxide | **s1** | 6.08 | 1609 |
| kobusone | **s2** | 6.56 | 1714 |
| reduced kobusone | **s3** | 5.85 | 1561 |
| caryophyllene alcohol | **2** | 6.05 | 1603 |
| presilphiperfolan-1β-ol | **2c** | 5.72 | 1534 |
| 2-(presilphiperfolanyl)-5-methylfuran | **10** | 6.93 | 1799 |
| 2-(presilphiperfolanyl)-4,5-dimethylfuran | **11** | 7.24 | 1872 |
| 2-(*trans*-*p*-menth-1-en-3-yl)-5-methylfuran | **12** | 5.48 | 1486 |
| 2-(*trans*-*p*-menth-1-en-3-yl)-4-methylfuran | **13** | 5.43 | 1476 |
| 2-(*trans*-*p*-menth-1-en-3-yl)-4,5-dimethylfuran | **14** | 5.94 | 1580 |
| 2-(*trans*-*p*-menth-1-en-3-yl)-1,5-dimethoxybenzene | **15** | 7.79 | 2007 |
| cyclic ether | **16** | 7.83 | 2017 |
| 2-(*trans*-*p*-menth-1-en-3-yl)-1-methylpyrrole | **17** | 6.16 | 1626 |
| 1-presilphiperfolanylpentane-1,4-dione | **18** | 8.23 | 2120 |
| 2-presilphiperfolanyl-1-methylpyrrole | **19** | 7.56 | 1949 |
| (2*Z*)-1-presilphiperfolanylpent-2-ene-1,4-dione | **20** | 8.30 | 2138 |

On temperature program B

| Compound | | Retention time (min) |
|---|---|---|
| 2-(presilphiperfolanyl)-5-methylfuran | **9** | 9.70 |
| 2-(presilphiperfolanyl)-5-methylfuran C12 epimer | **9b** | 9.94 |

## S2 First nucleophile screening

Reaction of caryophyllene alcohol **2** with 1,2-dimethylpyrrole, 1,2-dimethylindole and 2-methylfuran. Conditions: capsule **I** (10 mol%), HCl (3 mol%), nucleophile (2 equiv.), 30°C, 5 days, $CHCl_3$ as solvent.

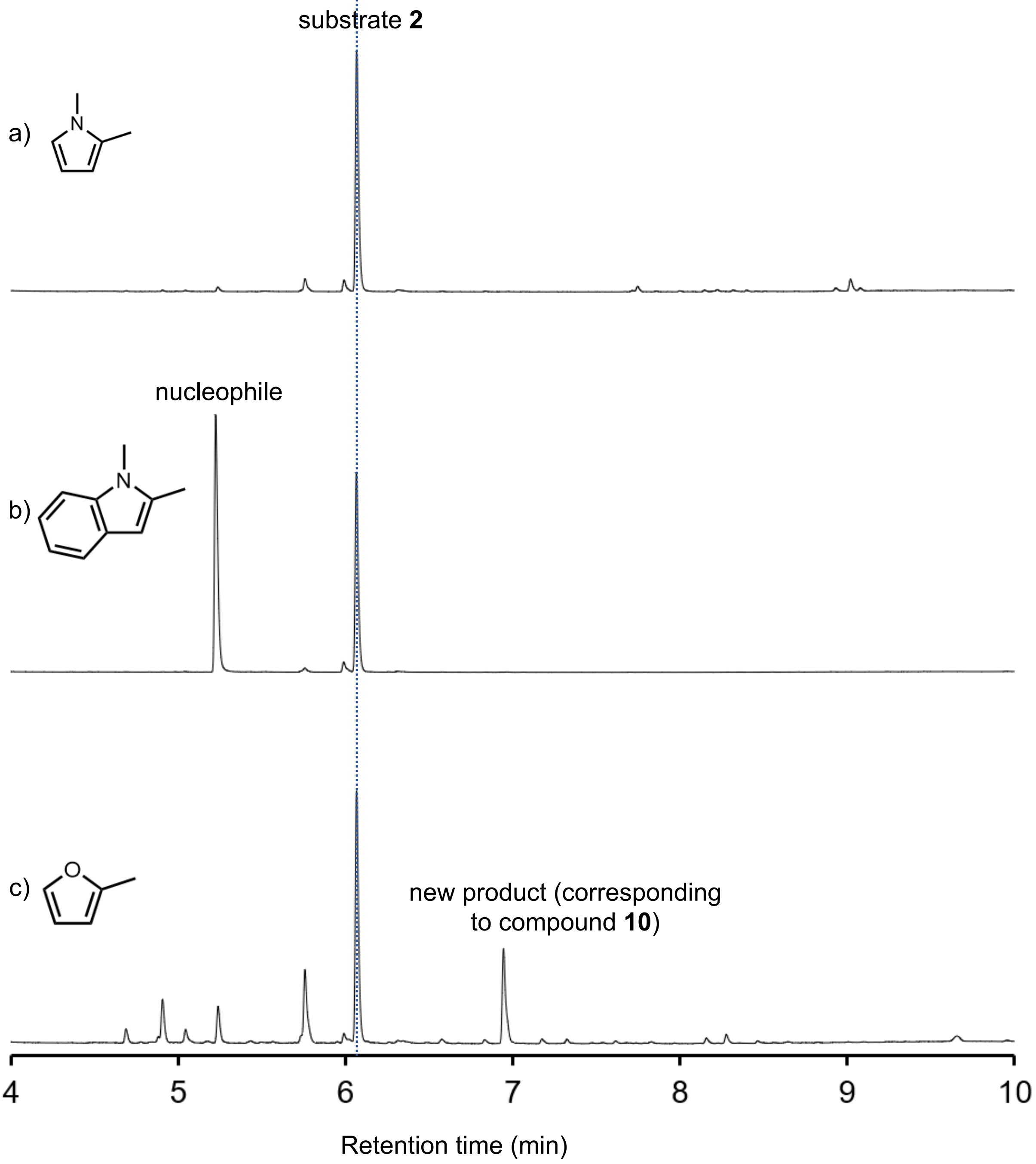

# S3 Optimization of the cyclization reaction conditions

A central composite design of 15 runs was made on the cyclization of caryophyllene alcohol **2** with 2-methylfurane **4**. RStudio (version 2024.09.1) was used for analysis and graphical representation. The following factors and levels were used:

- Temperature: 40, 50 ,60 °C
- Nucleophile: 5, 10 ,15 equiv.
- HCl : 2, 4, 6 mol%

The reaction was sampled after 24 h and after 48 h. Yield only increased slightly between 24 and 48 h thus 24 h was chosen as reaction duration and the design was performed at this sampling time. Another minor product (epimer of compound **10** at C12) was also produced during the reaction. Its production followed the formation of the major product, thus it could not be decreased. The following equation and its 3D representation were obtained:

$$\text{GC Yield} = -149 + 4.32x + 2.28y + 17.4z - 0.0443x^2 - 0.172y^2 - 1.40z^2 + 0.0815xy - 0.0738xz$$

*With* x = temperature, y = 2-methylfuran, z = HCl

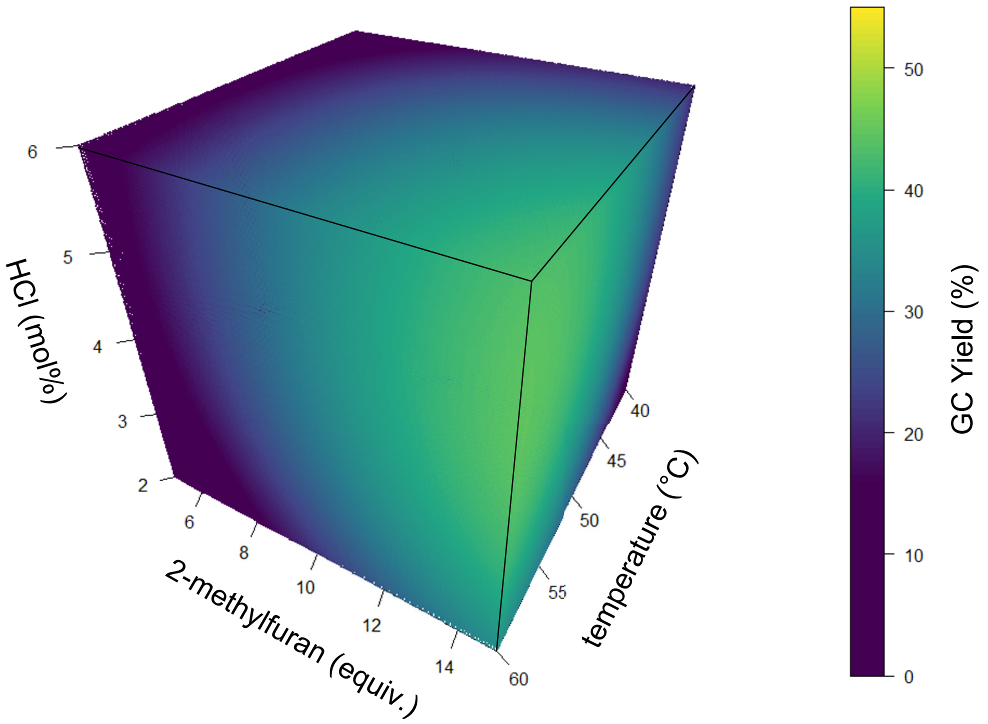


Since some nucleophiles may have stronger polarity or hydrogen bonding capability compared to 2-methylfuran **7** - that would have led to incomplete self-assembly of capsule **I** - the amount of nucleophile was not increased further. Moreover, higher temperatures were not tested due to the boiling point of chloroform. The optimized conditions were chosen as follows:

- Temperature: 60 °C
- Nucleophile: 10 eq.
- HCl : 4 mol%

Reaction time: 24 h

# S4 Synthesis of catalyst and substrates

## S4.1 Resorcin[4]arene 1

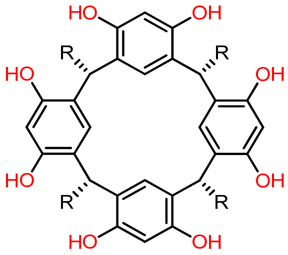


**1** (R= $C_{11}H_{23}$)
C72H112O8
1105.65 g/mol

Resorcin[4]arene was prepared according to the previously reported procedure.[3] Its water content was adjusted following the literature's method and found to be 8 $H_2O$/capsule **I** or 1.33 $H_2O$/resorcin[4]arene **1**.

## S4.2 Presilphiperfolan-1β-ol 2c

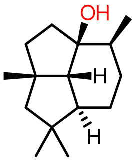


**2c**
$C_{15}H_{26}O$
222.37 g/mol

Presilphiperfolan-1β-ol **2c** was prepared according to the previously reported procedure.[3]

## S4.3 Kobusone s2

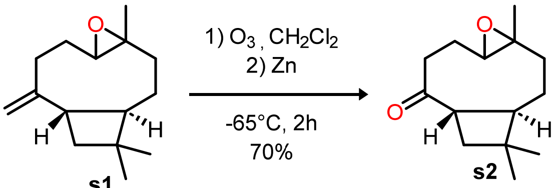


$C_{14}H_{22}O_2$
222.33 g/mol

(-)-Caryophyllene oxide **s1** (39.7 g, 180 mmol, 1 equiv.) and $CH_2Cl_2$ (500 mL) were placed in a three necked 1 L round-bottom flask fitted with a thermometer and a large olive stir bar. The flask was cooled down with an acetone-dry ice bath and oxygen was bubbled through. The exit of the round-bottom flask was fitted was a silicone tube leading directly to the fume hood aspiration. A trap was put between the flask and the ozone generator to protect the later. Oxygen was gently bubbled, and the ozone generator was turned on the maximum setting. The reaction mixture was kept between -55 and -65°C. The mixture gradually turned yellow and after 2 h quickly turned green blue. Ozone bubbling was continued for 5 min and then the ozone generator was switched off and oxygen was passed through the system for 5 min. 20 bar of a 20 L oxygen cylinder was used in the process. Then, still at -60 °C, zinc powder (17.7 g, 270 mmol , 1.5 equiv.) was added at once together with aqueous acetic acid (50%, 100 mL). The bubbling tube was removed and the system put under nitrogen and let come to room temperature overnight. The suspension was filtered over celite (porosity 3, 4 cm height, 10 cm

diameter, wetted with $CH_2Cl_2$) and the cake was rinsed with $CH_2Cl_2$ (100 mL). Water (250 mL) was added and the phases separated. The aqueous phase was extracted with $CH_2Cl_2$ (2 x 100 mL). The combined organic phases were dried with $MgSO_4$ and the solvents evaporated.

**Yield**: 93%, 37.2 g of kobusone **s2** as a clear oil that crystallizes upon storage. Stored at -20°C

**GC**: On GC temperature program A: rt(**s1**)=6.08min, rt(**s2**)=6.56min

**TLC**: 50/50 $Et_2O$/pentane, $KMnO_4$ stain, Rf(**s1**)=0.38, Rf(**s2**)=0

**NMR**: Spectral data were consistent with the literature[4]

## S4.4 Reduced kobusone s3

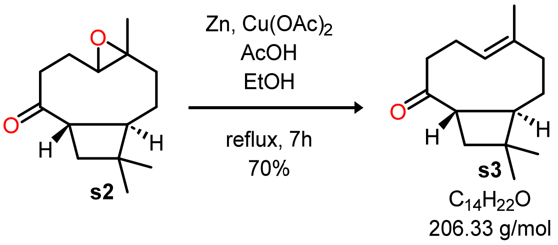


To a 250 mL round bottom flask containing ethanol (150 mL) and equipped with a large stir bar, was added zinc dust (29.4 g, 450 mmol, 20 equiv.), $Cu(OAc)_2{\cdot}H_2O$ (4.49 g, 22.5 mmol, 1 equiv.), acetic acid (4.05 g, 67.5 mmol, 3 equiv.) and kobusone **s2** (5.00 g, 22.5 mmol, 1 equiv.). The mixture was brought to reflux (temperature of the oil bath: 90°C) with good stirring. It was stirred until complete conversion of the starting material (as checked by GC or TLC) (7 h). After cooling, the content of the flask was filtered through celite (porosity 4, 2 cm height, 5 cm diameter, wetted with $CH_2Cl_2$). Sufficient amount of celite was added to the filtrate and the solvent evaporated. The solid deposit was purified using MPLC (silica 80 g, gradient 0 to 30% $Et_2O$ in pentane, detection at 200 and 220 nm). The product elutes around 10% $Et_2O$.

**Yield**: 70%, 3.25 g of a clear oil that crystalizes on cooling. Stored at -20°C

**GC**: On GC temperature program A, rt(**s2**) = 6.56 min, rt(**s3**) = 5.85 min

**TLC**: 50/50 $Et_2O$/pentane, $KMnO_4$ stain, Rf(**s2**)=0, Rf(**s3**)=0.43

**NMR**: Spectral data were consistent with the literature[4]

## S4.5 Caryophyllene alcohol 2

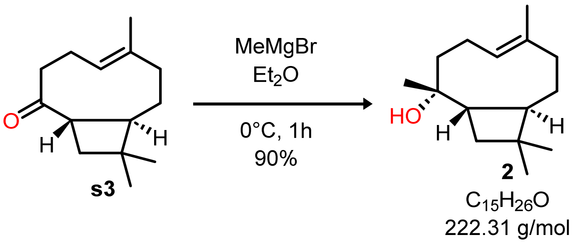


In an oven dried 100 mL three-necked round bottom flask equipped with a thermometer and stir bar, was placed the reduced kobusone **s3** (3.20 g, 15.5 mmol, 1 equiv.). Under nitrogen atmosphere, dry diethyl ether (50 mL) was added by syringe and the content of the flask was stirred until complete dissolution of the substrate. It was subsequently cooled with an ice water bath. Methylmagnesium bromide in diethyl ether (3 mol/L, 7.75 mL, 23.3 mmol, 1.5 equiv.) was added by syringe dropwise between 5 to 10°C over 10 min. Stirring was continued at that

temperature for 1 h. The mixture was then quenched by adding water (2 mL) slowly, then brine (40 mL) was added at once. The phases were separated, and the aqueous phase was extracted with diethyl ether (2 x 50 mL). The combined organic phases were dried with $MgSO_4$ and the solvents evaporated. The residue was purified using MPLC (silica 80 g, liquid injection with pentane, gradient 0 to 20% $Et_2O$ in pentane, detection at 200 and 220 nm) the product elutes around 10% $Et_2O$.

**Yield**: 90%, 3.09 g of caryophyllene alcohol **2** as a clear oil. Stored at -20°C

**GC**: On GC temperature program A, rt(**s3**)=5.85min, rt(**2**)=6.05min

**TLC**: 50/50 $Et_2O$/pentane, $KMnO_4$ stain, Rf(**s3**)=0.43, Rf(**2**)=0.58

**NMR**: Spectral data were consistent with the literature[4]

# S5 General procedures for screening scale reactions

## S5.1 General procedure A: Using resorcin[4]arene as catalyst

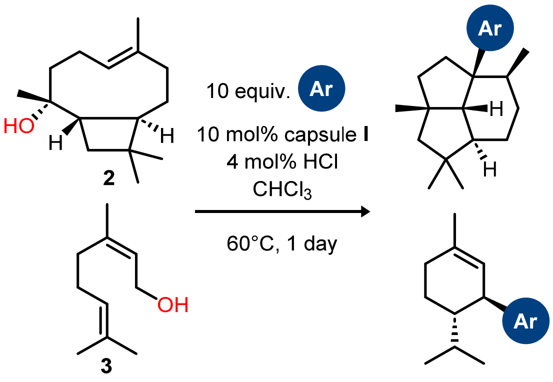


This procedure is a slight modification of the previously reported procedure.[3]

In a 2 mL crimpable vial (Duran Cat. No. 225600) equipped with a small magnetic stirrer was placed resorcin[4]arene **1** (11.1 mg, 10.0 µmol, 0.6 equiv. which corresponds to 10 mol% of capsule **I**), the internal standard *n*-dodecane (0.76 µL, 3.34 µmol, 0.2 equiv.), the substrate (16.7 µmol, 1 equiv.), the nucleophile (167 µmol, 10 equiv.) and chloroform (0.50 mL).

After stirring for a few seconds on a magnetic stirrer, a first sample was taken by dipping a Pasteur pipette tip in the mixture and washing it into an Eppendorf vial with 0.2 mL pentane with 0.08% DMSO. The capped Eppendorf vial was cooled to -20°C in a freezer for at least 30 min and then centrifuged for 2 min. The supernatant was transferred in a 1.5 mL GC vial and 1 mL of pentane was added. Analysis of this vial with GC temperature program A provided the sample for t0.

Hydrochloric acid solution in chloroform (0.668 µmol, 0.04 equiv.) was then added, and the vial was crimped (Fisherbrand Cat. No. 11737577). For the preparation of the hydrochloric acid solution in chloroform and the determination of its concentration, see cited literature.[3] It was then placed in an aluminium block heated to 60°C and stirred at 500 rpm.

After 24 h, the vial was removed from the heated block and let cool down. It was decapped and the same sampling procedure described above was followed to furnish a sample for t1.

## S5.2 General procedure B: Using $BF_3 \cdot Et_2O$ as catalyst

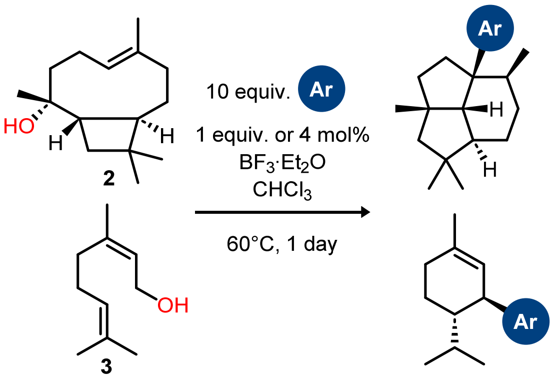


In a 2 mL crimpable vial (Duran Cat. No. 225600) equipped with a small magnetic stirrer was placed the internal standard *n*-dodecane (0.76 µL, 3.34 µmol, 0.2 equiv.), the substrate (16.7 µmol, 1 equiv.), the nucleophile (167 µmol, 10 equiv.) and chloroform (0.50 mL).

After stirring for a few seconds on a magnetic stirrer, a first sample was taken by dipping a Pasteur pipette tip in the mixture and washing it into a 1.5 mL GC vial with 1 mL of pentane. Analysis of this vial with GC temperature program A provided the sample for t0.

The corresponding amount of freshly made stock solution of $BF_3{\cdot}Et_2O$ was added (42.3 µL $BF_3{\cdot}Et_2O$ per mL $CHCl_3$, which corresponds to 2 µL, 0.668 µmol, for 4 mol% and 50 µL, 16.7 µmol, for 1 equiv.) and the vial was crimped (Fisherbrand Cat. No. 11737577). It was then placed in an aluminium block heated to 60°C and stirred at 500 rpm.

After 24 h, the vial was removed from the heated block and let cool down. It was decapped and the same sampling procedure described above was followed to furnish a sample for t1.

# S6 General procedure for large scale reactions

## S6.1 General procedure C: Using resorcin[4]arene as catalyst

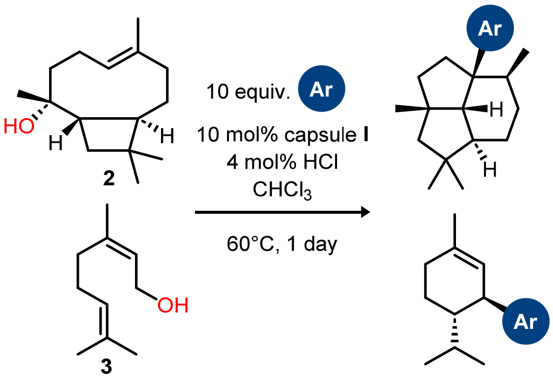


In a 12 mL screw cap vial (Fisherbrand Cat. No. 11576064) equipped with a small stir bar, was placed resorcin[4]arene (0.220 g, 0.199 mmol, 0.6 equiv. which corresponds to 10 mol% of capsule **I**), the substrate (0.332 mmol, 1 equiv.), the nucleophile (3.32 mmol, 10 equiv.) and $CHCl_3$ (10 mL). The vial was capped (Fisherbrand Cat. No. 1804233) and vigorously shaken.

Hydrochloric acid solution in chloroform (13.3 µmol, 0.04 equiv.) was then added, and the vial was capped. For the preparation of the hydrochloric acid solution in chloroform and the determination of its concentration, see cited literature.[3] It was then placed in an aluminium block heated to 60°C and stirred at 500 rpm.

After 24 h, the vial was removed from the heated block and let cool down. It was then worked up as described for each compound below.

## S6.2 General procedure D: Using $BF_3 \cdot Et_2O$ as catalyst

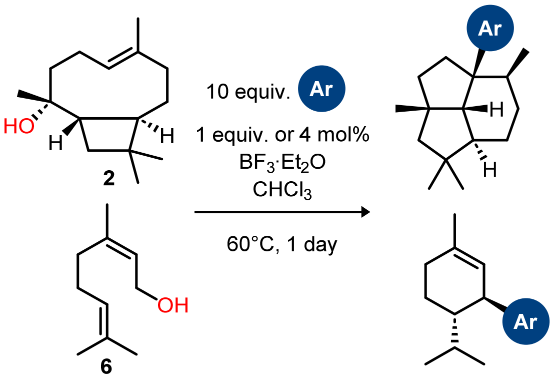


In a 12 mL screw cap vial (Fisherbrand Cat. No. 11576064) equipped with a small stir bar, was placed the substrate (0.332 mmol, 1 equiv.), the nucleophile (3.32 mmol, 10 equiv.) and $CHCl_3$ (10 mL). The vial was capped (Fisherbrand Cat. No. 1804233) and vigorously shaken.

The corresponding amount of $BF_3{\cdot}Et_2O$ (1.68 µL, 13.3 µmol, for 4 mol% and 42.1 µL, 0.332 mmol, for 1 equiv.) was added and the vial was capped. It was then placed in an aluminium block heated to 60°C and stirred at 500 rpm.

After 24 h, the vial was removed from the heated block and let cool down. It was then worked up as described for each compound below.

## S6.3 2-(presilphiperfolanyl)-5-methylfuran 10

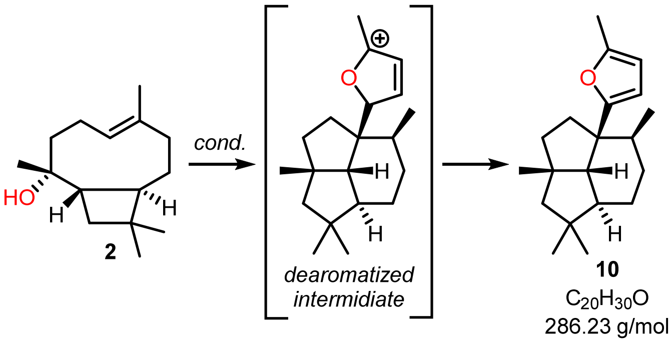


Using general procedure C, caryophyllene alcohol **2** (80.2 µL, 0.332 mmol, 1 equiv.) was used as substrate and 2-methylfuran (300 µL, 3.32 mmol, 10 equiv.) as the nucleophile.

After cooling, the content of the vial was transferred to a 50 mL round bottom flask and celite was added. The solvent was evaporated (down to 5 mbar and 40°C) and the resulting solid residue was manually purified on silica. It was loaded on a chromatography column (300 mm length, 13 mm diameter, 40 mL silica), eluted with pure pentane and collected in 8 ml fractions. The product eluted in the fractions 17 to 23. Each fraction was analyzed with GC temperature program B to determine which one contained a good purity of compound **10**. The fractions containing the minor product, the epimer of **10**, were subjected to column chromatography again. After three separations, most of compound **10** was recovered in pure form.

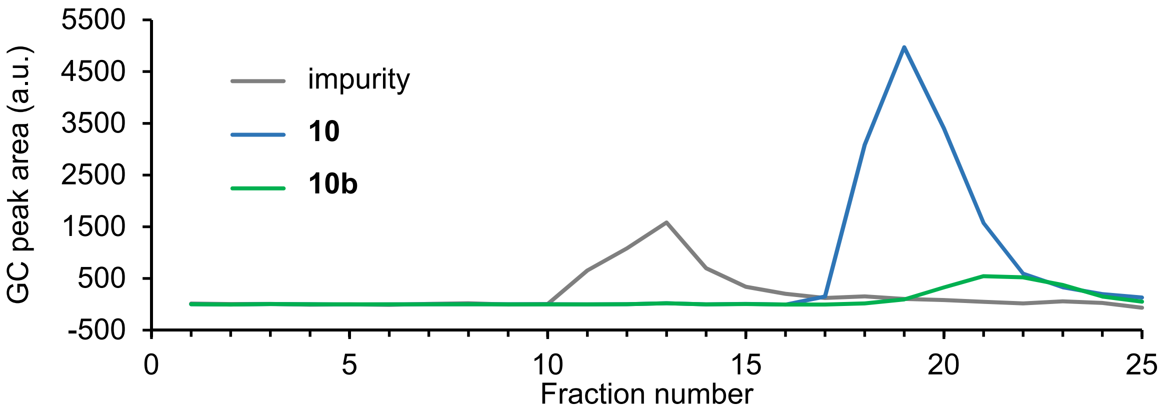


**Yield**: 29%, 27.8 mg of 2-(presilphiperfolanyl)-5-methylfuran **10** as a clear oil. Stored at -20°C.

**GC**: On GC temperature program A, rt(**10**) = 6.93 min

On GC temperature program B, rt(**10**) = 9.70 min

**TLC**: pentane, $KMnO_4$ stain, Rf(**10**)=0.47

**Optical rotation**: $[\alpha]_D^{20}$ = -22 deg·cm$^3$·dm$^{-1}$·g$^{-1}$ (c 0.1 g/100 mL, $CH_2Cl_2$)

**$^1$H NMR**: (600 MHz, $CDCl_3$) δ 5.93 (d, *J* = 3.0 Hz, 1H), 5.83 (d, *J* = 3.1 Hz, 1H), 2.42 – 2.36 (m, 1H), 2.26 (s, 3H), 2.20 (d, *J* = 12.8 Hz, 1H), 1.86 – 1.80 (m, 1H), 1.80 – 1.75 (m, 1H), 1.67 – 1.52 (m, 4H), 1.51 (s, 2H), 1.50 – 1.45 (m, 1H), 1.41 – 1.32 (m, 2H), 0.96 (s, 3H), 0.88 (s, 3H), 0.88 (s, 3H), 0.54 (d, *J* = 7.0 Hz, 3H)

**$^{13}$C NMR**: (151 MHz, $CDCl_3$) δ 157.8, 148.9, 105.0, 104.3, 60.1, 58.6, 50.4, 47.4, 44.5, 41.7, 39.1, 37.6, 35.6, 30.0, 27.9, 27.6, 21.1, 19.8, 16.9, 12.9

**IR**: $\nu_{max}$(ATR)/cm$^{-1}$ 2920s, 2858m, 1553w, 1452m, 1369w, 1220m, 1021m, 774s

**HRMS**: (ESI+) m/z calculated for the presilphiperfolanyl fragment $C_{15}H_{25}^+$ [M$^+$]: 205.1951; found 205.1946

## S6.4 2-(presilphiperfolanyl)-5-methylfuran epimer 10b

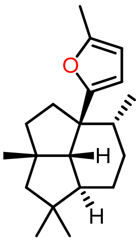


**x**

$C_{20}H_{30}O$
286.23 g/mol

After three flash chromatography as described for compound **10**, the product was not purified further and obtained as mixture in a 1.8/1 ratio of **10**/**10b**.

**Yield**: 28%, 26.7 mg of a mixture with a 1.8/1 ratio of **10**/**10b** as a clear oil. Stored at -20°C

**GC**: On GC temperature program A, rt(**10b**) = 6.93 min

On GC temperature program B, rt(**10b**) = 9.94 min

**$^{1}$H NMR**: (600 MHz, $CDCl_3$) δ 5.93 (d, *J* = 3.0 Hz, 1H), 5.83 (d, *J* = 3.1 Hz, 1H), 2.42 – 2.36 (m, 1H), 2.26 (s, 3H), 2.20 (d, *J* = 12.8 Hz, 1H), 1.86 – 1.80 (m, 1H), 1.80 – 1.75 (m, 1H), 1.67 – 1.52 (m, 4H), 1.51 (s, 2H), 1.50 – 1.45 (m, 1H), 1.41 – 1.32 (m, 2H), 0.96 (s, 3H), 0.88 (s, 3H), 0.88 (s, 3H), 0.54 (d, *J* = 7.0 Hz, 3H)

**$^{13}$C NMR**: (151 MHz, $CDCl_3$) δ 157.8, 148.9, 105.0, 104.3, 60.1, 58.6, 50.4, 47.4, 44.5, 41.7, 39.1, 37.6, 35.6, 30.0, 27.9, 27.6, 21.1, 19.8, 16.9, 12.9

## S6.5 2-(presilphiperfolanyl)-4,5-dimethylfuran 11

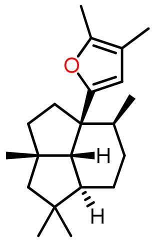


**11**
$C_{21}H_{32}O$
300.49 g/mol

Using general procedure C, caryophyllene alcohol **2** (80.2 µL, 0.332 mmol, 1 equiv.) was used as substrate and 2,3-dimethylfuran **6** (351 µL, 3.32 mmol, 10 equiv.) as the nucleophile.

After cooling, the content of the vial was transferred to a 50 mL round bottom flask and celite was added. The solvent was evaporated (down to 5 mbar and 40°C) and the resulting solid residue was manually purified on silica. It was loaded on a chromatography column (300 mm length, 13 mm diameter, 40 mL silica), eluted with pure pentane and collected in 8 ml fractions. The fractions were analyzed by GC as described for product **9**. The product eluted in the fractions 18 to 25.

**Yield**: 23%, 23.0 mg of 2-(presilphiperfolanyl)-4,5-dimethylfuran **11** as a clear oil. Stored at -20°C

**GC**: On GC temperature program B, rt(**11**) = 7.24 min

**TLC** : pentane, $KMnO_4$ stain, Rf(**11**) = 0.77

**Optical rotation:** $[\alpha]_D^{20}$ = -19 deg·cm$^3$·dm$^{-1}$·g$^{-1}$ (c 0.1 g/100 mL, $CH_2Cl_2$)

**$^{1}$H NMR**: (600 MHz, $CDCl_3$) δ 5.81 (s, 1H), 2.35 (ddd, *J* = 13.1, 7.8, 3.5 Hz, 1H), 2.17 (d, *J* = 11,1 Hz, 1H), 2.15 (s, 3H), 1.89 (s, 3H), 1.81 – 1.72 (m, 2H), 1.63 – 1.44 (m, 5H), 1.49 (s, 2H), 1.37 – 1.32 (m, 2H), 0.95 (s, 3H), 0.89 (s, 3H), 0.86 (s, 3H), 0.53 (d, *J* = 6.9 Hz, 3H)

**$^{13}$C NMR**: (151 MHz, $CDCl_3$) δ 157.3, 144.9, 113.5, 108.7, 61.0, 59.5, 51.2, 48.4, 45.7, 42.6, 40.0, 38.5, 36.5, 31.0, 28.8, 28.6, 22.1, 20.8, 17.9, 11.6, 10.2

**IR**: $\nu_{max}$(ATR)/cm$^{-1}$ 2921s, 2858m, 1706w, 1554m, 1451m, 1369m, 1222m, 1150m, 941m, 765m, 625m

**HRMS**: (ESI+) m/z calculated for the presilphiperfolanyl fragment $C_{15}H_{25}^+$ [M$^+$]: 205.1951; found 205.1952

## S6.6 2-(*trans*-*p*-menth-1-en-3-yl)-5-methylfuran 12

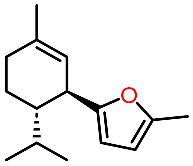

**12**
$C_{15}H_{22}O$
218.34 g/mol

Using general procedure C, nerol **3** (58.5 µL, 0.332 mmol, 1 equiv.) was used as substrate and 2-methylfuran **4** (300 µL, 3.32 mmol, 10 equiv.) as the nucleophile.

After cooling, the reaction mixture was filtered through a plug of basic aluminium oxide, washed with $CHCl_3$ (10 mL), and the volume was reduced to less than 5 mL. This solution was then directly injected in the recycling GPC. After two cycles the main peak (detection at 250 nm), first in order of elution, was collected and separation was continued. The main peak is resorcin[4]arene. After 25 cycles in total, the largest peak remaining is collected and the solvent evaporated.

**Yield**: 55%, 40.0 mg of 2-(*trans*-*p*-menth-1-en-3-yl)-5-methylfuran **12** as a clear oil. Stored at -20°C

**GC**: On GC temperature program A, rt(**12**) = 5.48 min

**TLC**: pentane, UV 254 nm, Rf(**12**) = 0.74

**$^1$H NMR**: (600 MHz, $CDCl_3$) δ 5.84 (s, 2H), 5.32 (h, *J* = 1.5 Hz, 1H), 3.28 (dp, *J* = 8.7, 2.4 Hz, 1H), 2.26 (s, 3H), 2.01 – 1.95 (m, 1H), 1.73 – 1.69 (m, 2H), 1.68 (s, 3H), 1.59 – 1.54 (m, 2H), 1.42 – 1.35 (m, 1H), 0.92 (d, *J* = 6.9 Hz, 3H), 0.81 (d, *J* = 6.9 Hz, 3H)

**$^{13}$C NMR**: (151 MHz, $CDCl_3$) δ 157.3, 150.3, 134.7, 122.6, 105.6, 105.6, 43.5, 39.2, 29.5, 27.6, 23.4, 21.6, 21.3, 17.1, 13.5

**IR**: $\nu_{max}$(ATR)/cm$^{-1}$ 2952m, 2919m, 2867m, 1558m, 1447m, 1382m, 1364m, 1218m, 1152w, 1018s, 962m, 776s

**HRMS**: (ESI+) m/z calculated for $C_{15}H_{22}O$ [M+H$^+$]: 219.1743; found 219.1747

## S6.7 2-(*trans*-*p*-menth-1-en-3-yl)-4-methylfuran 13

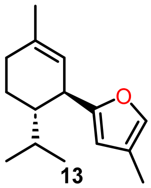

**13**
$C_{15}H_{22}O$
218.34 g/mol

Using general procedure D with 1 equiv. of $BF_3 \cdot Et_2O$, nerol **3** (58.5 µL, 0.332 mmol, 1 equiv.) was used as substrate and 3-methylfuran **5** (296 µL, 3.32 mmol, 10 equiv.) as the nucleophile.

After cooling, the reaction mixture was filtered through a plug of basic aluminium oxide, washed with $CHCl_3$ (10 mL), and the volume was reduced to less than 5 mL. This solution was then directly injected in the recycling GPC. After 40 cycles, everything was collected in 12 fractions of 18 mL each. They were each analyzed using GC with temperature program A to determine the fractions containing pure product **13**.

**Yield**: 15%, 11.1 mg of 2-(*trans*-*p*-menth-1-en-3-yl)-4-methylfuran **13** as a clear oil. Stored at -20°C

**GC**: On GC temperature program A, rt(**13**) = 5.43 min

**TLC**: pentane, UV 254 nm, Rf(**13**) = 0.78

**$^1$H NMR**: (600 MHz, $CDCl_3$) δ 7.22 (d, *J* = 1.8 Hz, 1H), 6.15 (d, *J* = 1.8 Hz, 1H), 5.24 – 5.22 (m, 1H), 3.37 – 3.32 (m, 1H), 2.15 – 2.06 (m, 1H), 2.03 – 1.99 (m, 1H), 1.96 (s, 3H), 1.78 – 1.73 (m, 1H), 1.71 – 1.69 (m, 1H), 1.69 (s, 3H), 1.50 (pd, *J* = 6.9, 3.0 Hz, 1H), 1.36 (qd, *J* = 11.5, 5.5 Hz, 1H), 0.87 (d, *J* = 7.0 Hz, 3H), 0.77 (d, *J* = 6.9 Hz, 3H)

**$^{13}$C NMR**: (151 MHz, $CDCl_3$) δ 153.2, 139.9, 135.2, 123.2, 114.5, 112.8, 43.6, 37.8, 30.5, 28.2, 23.6, 22.5, 21.4, 16.7, 10.0

**IR**: $v_{max}$(ATR)/cm$^{-1}$ 2952m, 2920m, 2865m, 1507m, 1460m, 1375m, 1149m, 1102w, 1073m, 885m, 843m, 730s, 694m

**HRMS**: (ESI+) m/z calculated for the *trans*-*p*-menth-1-en-3-yl fragment $C_{10}H_{17}^+$ [$M^+$]: 135.1168; found 135.1164

## S6.8 2-(*trans*-*p*-menth-1-en-3-yl)-4,5-dimethylfuran 14

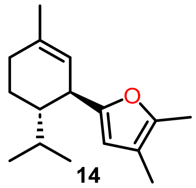

**14**
$C_{16}H_{24}O$
232.37 g/mol

Using general procedure D with 1 equiv. of $BF_3{\cdot}Et_2O$, nerol **3** (58.5 µL, 0.332 mmol, 1 equiv.) was used as substrate and 2,3-dimethylfuran **6** (351 µL, 3.32 mmol, 10 equiv.) as the nucleophile.

After cooling, the reaction mixture was filtered through a plug of basic aluminium oxide, washed with $CHCl_3$ (10 mL), and the volume was reduced to less than 5 mL. This solution was then directly injected in the recycling GPC. After two cycles the first broad peak (detection at 250 nm), was collected and separation was continued. After a total of 40 cycles, everything was collected in 12 fractions of 18 mL each. They were each analyzed using GC with temperature program A to determine the fractions containing pure product **14**.

**Yield**: 69%, 53.1 mg of 2-(*trans*-*p*-menth-1-en-3-yl)-4,5-dimethylfuran **14** as a clear oil. Stored at -20°C

**GC**: On GC temperature program A, rt(**14**) = 5.94 min

**TLC**: pentane, UV 254 nm, Rf(**14**) = 0.75

**$^1$H NMR**: (600 MHz, $CDCl_3$) δ 5.74 (s, 1H), 5.30 (q, *J* = 1.6 Hz, 1H), 3.22 (dp, *J* = 7.7, 2.5 Hz, 1H), 2.16 (s, 3H), 2.04 – 1.93 (m, 2H), 1.89 (s, 3H), 1.74 – 1.68 (m, 2H), 1.67 (s, 3H),1.52 – 1.56 (m, 1H), 1.40 – 1.32 (m, 1H), 0.92 (d, *J* = 6.9 Hz, 3H), 0.80 (d, *J* = 6.9 Hz, 3H)

**$^{13}$C NMR**: (151 MHz, $CDCl_3$) δ 156.2, 145.4, 134.8, 122.9, 114.2, 108.4, 43.6, 39.4, 29.7, 27.8, 23.7, 21.8, 21.6, 17.3, 11.5, 10.1

**IR**: $v_{max}$(ATR)/cm$^{-1}$ 2920w, 1752m, 1674w, 1442m, 1334m, 1193m, 907s, 832m, 728m

**HRMS**: (ESI+) m/z calculated for the *trans*-*p*-menth-1-en-3-yl fragment $C_{10}H_{17}^+$ [$M^+$]: 135.1168; found 135.1170

## S6.9 2-(*trans*-*p*-menth-1-en-3-yl)-1,5-dimethoxybenzene 15

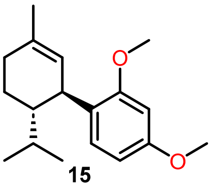

**15**
$C_{18}H_{26}O_2$
274.40 g/mol

Using general procedure C, nerol **3** (58.5 µL, 0.332 mmol, 1 equiv.) was used as substrate and 1,3-dimethoxybenzene **7** (435 µL, 3.32 mmol, 10 equiv.) as the nucleophile.

After cooling, the content of the vial was transferred to a 50 mL round bottom flask and celite was added. The solvent was evaporated (down to 5 mbar and 40°C) and the resulting solid residue was purified using MPLC (40 g, eluting with 0 to 30% $Et_2O$ in pentane, 200 and 220 nm detection) the product elutes around 20% $Et_2O$, just before the large peak of 1,3-dimethoxybenzene.

**Yield**: 51%, 46.8 mg of 2-(*trans*-*p*-menth-1-en-3-yl)-1,5-dimethoxybenzene **15** as a clear oil. Stored at -20°C

**GC**: On GC temperature program A, rt(**15**) = 6.16 min

**TLC**: 5/95 $Et_2O$/pentane, UV 254 nm, Rf(**15**) = 0.34

**$^1$H NMR**: (600 MHz, $CDCl_3$) δ 7.03 (d, *J* = 8.0 Hz, 1H), 6.46 – 6.43 (m, 2H), 5.20 – 5.17 (m, 1H), 3.79 (s, 3H), 3.78 (s, 3H), 3.72 – 3.68 (m, 1H), 2.05 – 1.95 (m, 2H), 1.69 (s, 3H), 1.68 – 1.64 (m, 1H), 1.56 – 1.50 (m, 1H), 1.46 – 1.33 (m, 2H), 0.89 (d, *J* = 6.8 Hz, 3H), 0.82 (d, *J* = 6.8 Hz, 3H)

**$^{13}$C NMR**: (151 MHz, $CDCl_3$) δ 158.9, 158.4, 134.1, 129.3, 127.4, 125.5, 104.2, 98.3, 55.4, 55.4, 46.2, 37.0, 29.8, 27.7, 23.8, 21.9, 21.7, 17.7

**IR**: $\nu_{max}$(ATR)/cm$^{-1}$ 2949m, 2921m, 1607m, 1583m, 1500s, 1460m, 1287m, 1255m, 1204s, 1153s, 1114m, 1036s, 923m, 831m, 792m

**HRMS**: (ESI+) m/z calculated for $C_{18}H_{26}O_2$ [$M+Ag^+$]: 381.0980; found 381.0978

## S6.10 Cyclic ether 16

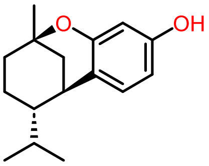


**16**
$C_{16}H_{22}O_2$
246.35 g/mol

Using general procedure C, nerol **3** (58.5 µL, 0.332 mmol, 1 equiv.) was used as substrate and resorcinol **8** (286 mg, 3.32 mmol, 10 equiv.) as the nucleophile.

After cooling, the content of the vial was transferred to a 50 mL round bottom flask and celite was added. The solvent was evaporated (down to 5 mbar and 40°C) and the resulting solid residue was purified using MPLC (40 g, eluting with 0 to 50% $Et_2O$ in pentane, 200 and 220 nm detection) the product elutes around 40% $Et_2O$, just before the large peak of resorcinol.

**Yield**: 69%, 56.1 mg of cyclic ether **16** as a viscous light red oil. Stored at -20°C

**GC**: On GC temperature program A, rt(**16**) = 7.83 min

**TLC**: 50/50 $Et_2O$/pentane, UV 254 nm, Rf(**16**) = 0.42, Rf(resorcinol) = 0.21

**$^1$H NMR**: (600 MHz, $CDCl_3$) δ 6.81 (d, *J* = 7.8 Hz, 1H), 6.33 – 6.28 (m, 2H), 4.94 (s, 1H), 2.97 (q, *J* = 3.1 Hz, 1H), 1.86 (dd, *J* = 13.2, 2.6 Hz, 1H), 1.78 (dp, *J* = 10.5, 6.7 Hz, 1H), 1.74 – 1.70 (m, 1H), 1.62 – 1.57 (m, 1H), 1.55 (dd, *J* = 13.7, 5.5 Hz, 1H), 1.48 – 1.43 (m, 2H), 1.33 (s, 3H), 1.16 – 1.11 (m, 1H), 1.05 (d, *J* = 6.6 Hz, 3H), 0.94 (d, *J* = 6.6 Hz, 3H)

**$^{13}$C NMR**: (151 MHz, $CDCl_3$) δ 157.5, 154.9, 128.8, 120.4, 106.5, 102.0, 75.0, 47.4, 35.0, 33.9, 30.9, 29.4, 26.3, 22.1, 21.3, 19.9

**IR**: $v_{max}$(ATR)/$cm^{-1}$ 3374br, 2924m, 2865w, 1616m, 1592m, 1501m, 1448m, 1142s, 1125s, 1109m, 1003m, 983s, 842m, 735s

**HRMS**: (ESI+) m/z calculated for $C_{16}H_{22}O_2$ [$M+H^+$]: 247.1693; found 247.1692

## S6.11 2-(*trans*-*p*-menth-1-en-3-yl)-1-methylpyrrole 17

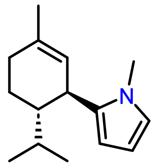

**17**
$C_{15}H_{23}N$
217.36 g/mol

Using general procedure D with 4 mol% of $BF_3{\cdot}Et_2O$, nerol **3** (58.5 µL, 0.332 mmol, 1 equiv.) was used as substrate and N-methylpyrrole **9** (295 µL, 3.32 mmol, 10 equiv.) as the nucleophile.

After cooling, the reaction mixture was filtered through a plug of basic aluminium oxide, washed with $CHCl_3$ (10 mL), and the volume was reduced to less than 5 mL. This solution was then directly injected in the recycling GPC. After 6 cycles the main peak (detection at 250 nm), last in order of elution, was collected and separation was continued. The main peak is N-methylpyrrole. After 14 cycles in total, the largest peak remaining is collected and the solvent evaporated.

**Yield**: 15%, 11.0 mg of 2-(*trans*-*p*-menth-1-en-3-yl)-1-methylpyrrole **17** as a yellow oil. Stored at -20°C

**GC**: On GC temperature program A, rt(**17**) = 6.16 min

**TLC**: Aluminium oxide plate, 20/80 $Et_2O$/pentane, UV 254 nm, Rf(**17**) = 0.69

**$^1$H NMR**: (600 MHz, $CDCl_3$) δ 6.51 (t, *J* = 2.3 Hz, 1H), 6.05 (t, *J* = 3.1 Hz, 1H), 5.84 (dd, *J* = 3.5, 1.9 Hz, 1H), 5.30 – 5.26 (m, 1H), 3.56 (s, 3H), 3.33 (dp, *J* = 7.6, 2.4 Hz, 1H), 2.00 – 1.97 (m, 2H), 1.80 – 1.72 (m, 1H), 1.70 – 1.68 (m, 1H), 1.67 (s, 3H), 1.52 – 1.46 (m, 1H), 1.44 – 1.37 (m, 1H), 0.94 (d, *J* = 6.9 Hz, 3H), 0.82 (d, *J* = 6.9 Hz, 3H)

**$^{13}$C NMR**: (151 MHz, $CDCl_3$) δ 136.4, 134.1, 123.4, 121.4, 106.6, 106.4, 44.4, 37.2, 34.1, 29.4, 27.6, 23.6, 21.8, 21.5, 17.8

**IR**: $\nu_{max}$(ATR)/cm$^{-1}$ 2951s, 2921s, 2866m, 1706w, 1488m, 1460m, 1445m, 1382w, 1364w, 1297m, 1086w, 701s

**HRMS**: (ESI+) m/z calculated for $C_{15}H_{23}N$ [M+Ag$^+$]: 324.0881; found 324.0876

# S7 Synthesis of 2-(presilphiperfolanyl)-5-methylfuran 10 derivatives

## S7.1 1-presilphiperfolanylpentane-1,4-dione 18

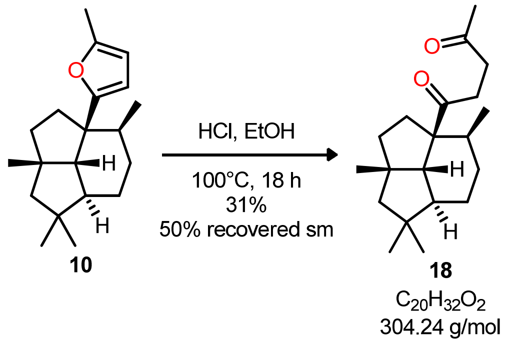


Furan derivative **10** (110 mg, 384 µmol, 1 equiv.), 37% aqueous HCl (4 mL) and ethanol (4 mL) were placed in a 10 mL vial (Fisherbrand Cat. No. 11576064 and 1804233) equipped with a stir bar. The vial was heated in an aluminum block at 100°C overnight (18 h). After cooling, the mixture was transferred to a separation funnel and $CH_2Cl_2$ (20 mL) and brine (20 mL) were added. The phases were separated and the aqueous was extracted with $CH_2Cl_2$ (2 x 10 mL). The collected organic phases were dried with $MgSO_4$ and the solvents evaporated. The residue was purified using MPLC (40 g, eluting with 0 to 20% $Et_2O$ in pentane, 200 and 220 nm detection) the product elutes around 10% $Et_2O$. The first peak coming with the elution front is unreacted furan **10**.

**Yield**: 31%, 30.8 mg of 1-presilphiperfolanylpentane-1,4-dione **18** as a clear oil that crystallizes on cooling. Stored at -20°C

50%, 54.9 mg of recovered starting furan **10**

**GC**: On GC temperature program A, rt(**18**) = 8.23 min

**TLC**: 20/80 $Et_2O$/pentane, $KMnO_4$ stain, Rf(**18**) = 0.26

**Optical rotation:** $[\alpha]_D^{20}$ = -31 deg·cm$^3$·dm$^{-1}$·g$^{-1}$ (c 0.1 g/100 mL, $CH_2Cl_2$)

**$^1$H NMR**: (600 MHz, $CDCl_3$) δ2.85 (qt, $J$ = 18.5, 6.4 Hz, 2H), 2.69 (dt, $J$ = 17.9, 6.4 Hz, 1H), 2.61 (dt, $J$ = 17.9, 6.3 Hz, 1H), 2.55 – 2.50 (m, 1H), 2.29 (d, $J$ = 12.6 Hz, 1H), 2.22 (s, 3H), 1.84 – 1.78 (m, 1H), 1.71 (dt, $J$ = 13.5, 8.9 Hz, 1H), 1.65 – 1.57 (m, 2H), 1.55 – 1.44 (m, 5H), 1.41 – 1.30 (m, 2H), 0.94 (s, 3H), 0.94 (s, 3H), 0.85 (s, 3H), 0.75 (d, $J$ = 7.2 Hz, 3H)

**$^{13}$C NMR**: (151 MHz, $CDCl_3$) δ 214.2, 207.9, 63.2, 59.2, 58.9, 48.8, 45.6, 42.5, 40.3, 37.2, 37.0, 35.4, 34.6, 30.4, 30.2, 29.1, 28.7, 22.1, 20.4, 17.8

**IR**: $\nu_{max}$(ATR)/cm$^{-1}$ 2928s, 2855m, 1709s, 1683s, 1565w, 1451m, 1361m, 1351m, 1193m, 1165s, 1079m, 984m, 477m, 433m, 419m

**HRMS**: (ESI+) m/z calculated for $C_{20}H_{32}O_2$ [M+Na$^+$]: 327.2295; found 327.2300

## S7.2 2-presilphiperfolanyl-1-methylpyrrole 19

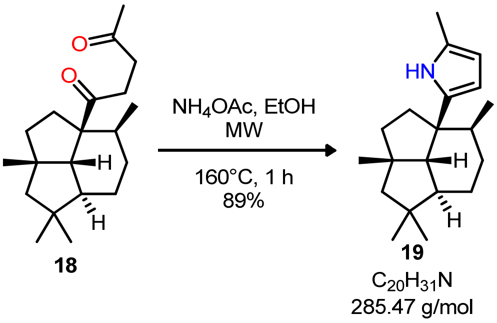


The diketone **18** (25.0 mg, 82.1 µmol, 1 equiv.), ammonium acetate (1.27 g, 16.4 mmol, 200 equiv.) and ethanol (10 mL) were placed in a 20 mL microwave oven vial (AntonPaar). The mixture was heated to 160°C for 1 h in the microwave oven. Another portion of ammonium acetate (1.27 g, 16.4 mmol, 200 equiv.) was added and heating was continued for 30 min at 160°C. After cooling, $CH_2Cl_2$ (50 mL) was added, and the mixture was extracted with NaOH (2 mol/L, 2 x 50 mL). The organic phase was then dried with $MgSO_4$ and the solvents evaporated. The residue was purified on recycling GPC. After 10 cycles, 12 fractions of 18 mL were collected and each analyzed by GC or TLC to determine the fractions containing pure product **19**.

**Yield**: 89%, 21.0 mg of 2-presilphiperfolanyl-1-methylpyrrole **19** as a yellow viscous oil. Stored at -20°C

**GC**: On GC temperature program A, rt(**19**) = 7.56 min

**TLC**: Aluminium oxide plate, 20/80 $Et_2O$/pentane, UV 254 nm, Rf(**19**) = 0.74, Rf(**18**) = 0.50

**Optical rotation:** $[\alpha]_D^{20}$ = -38 deg·cm$^3$·dm$^{-1}$·g$^{-1}$ (c 0.1 g/100 mL, $CH_2Cl_2$)

**$^1$H NMR**: (600 MHz, $CDCl_3$) δ 7.58 (s, 1H), 5.86 (t, *J* = 3.0 Hz, 1H), 5.78 (s, 1H), 2.27 (s, 3H), 2.27 – 2.23 (m, 1H), 2.08 (d, *J* = 12.7 Hz, 1H), 1.89 (ddd, *J* = 13.1, 9.9, 8.0 Hz, 1H), 1.84 – 1.78 (m, 1H), 1.65 – 1.59 (m, 2H), 1.58 (s, 1H), 1.53 (s, 2H), 1.52 – 1.47 (m, 1H), 1.46 – 1.34 (m, 3H), 0.98 (s, 3H), 0.92 (s, 3H), 0.90 (s, 3H), 0.51 (d, *J* = 6.9 Hz, 3H)

**$^{13}$C NMR**: (151 MHz, $CDCl_3$) δ 135.3, 125.5, 106.0, 105.0, 62.5, 59.3, 50.3, 48.4, 45.9, 42.5, 40.1, 38.4, 37.5, 31.0, 29.0, 28.7, 22.1, 20.9, 18.4, 13.5

**IR**: $\nu_{max}$(ATR)/cm$^{-1}$ 2920s, 2857m, 1721w, 1458m, 1370m, 1272m, 1125m, 750s, 475w

**HRMS**: (ESI+) m/z calculated for $C_{20}H_{31}N$ [M+H$^+$]: 286.2529; found 286.2523

## S7.3 (2Z)-1-presilphiperfolanylpent-2-ene-1,4-dione 20

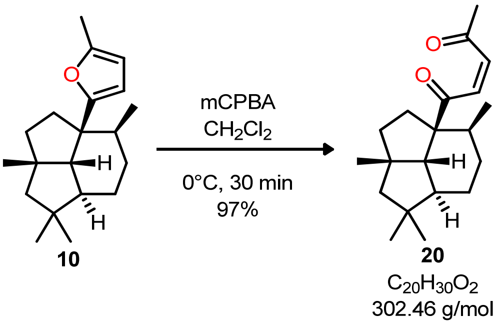

The furan derivative **10** (100 mg, 0.349 mmol, 1 equiv.) and $CH_2Cl_2$ (20 mL) were placed in a two necked round bottomed flask fitted with a thermometer and cooled with an ice-water bath. mCPBA (66.2 mg, 0.384 mmol, 1.1 equiv.) in solution in $CH_2Cl_2$ (20 mL) was added dropwise over 20 min between 0-5°C. At the end of the addition, the presence of the furan was checked by GC or TLC. If some substrate was still present, another portion of mCPBA (30.1 mg, 0.175 mmol, 0.5 equiv.) was added. The mixture was stirred for 15 min and then transferred to a separation funnel. NaOH (2 mol/L, 50 mL) was added and the phase separated. The aqueous phase was extracted with $CH_2Cl_2$ (2 x 30 mL). The combined organic phases were dried with $MgSO_4$ and evaporated. The residue was purified on silica using MPLC (12 g, liquid injection with pentane, eluting with 0 to 30% $Et_2O$ in pentane, 200 and 220 nm detection) the product elutes around 10% $Et_2O$.

**Yield**: 97%, 102 mg of unsaturated diketone **20** as a clear oil

**GC**: On GC temperature program A, rt(**20**) = 8.30 min

**TLC**: 20/80 $Et_2O$/pentane, UV 254 nm, Rf(**20**) = 0.19

**Optical rotation**: $[\alpha]_D^{20}$ = -34 deg·cm$^3$·dm$^{-1}$·g$^{-1}$ (c 0.1 g/100 mL, $CH_2Cl_2$)

**$^1$H NMR**: (600 MHz, $CDCl_3$) δ 6.56 (d, *J* = 11.9 Hz, 1H), 6.31 (d, *J* = 11.9 Hz, 1H), 2.45 (ddd, *J* = 13.6, 8.0, 5.8 Hz, 1H), 2.34 (s, 3H), 2.27 (d, *J* = 12.7 Hz, 1H), 1.95 – 1.89 (m, 1H), 1.77 (dt, *J* = 13.4, 8.0 Hz, 1H), 1.65 (ddd, *J* = 13.9, 8.4, 5.8 Hz, 1H), 1.60 – 1.48 (m, 6H), 1.44 (dt, *J* = 13.1, 7.8 Hz, 1H), 1.37 – 1.29 (m, 1H), 0.97 (s, 3H), 0.95 (s, 3H), 0.87 (s, 3H), 0.85 (d, *J* = 7.3 Hz, 3H)

**$^{13}$C NMR**: (151 MHz, $CDCl_3$) δ 204.3, 203.9, 142.0, 128.9, 62.0, 58.3, 58.3, 48.6, 46.0, 42.0, 40.4, 36.1, 34.3, 29.5, 29.3, 29.1, 28.7, 22.2, 19.9, 18.0

**IR**: $\nu_{max}$(ATR)/cm$^{-1}$ 2924s, 2859m, 1700s, 1675s, 1601m, 1459m, 1386m, 1348m, 1164s, 1076m, 976m, 784m, 492m

**HRMS**: (ESI+) m/z calculated for $C_{20}H_{30}O_2$ [M+Na$^+$]: 325.2144; found 325.2138

## S8 Screening of control reactions

Control reactions using different acids and omitting the capsule **I** with nerol **3** as substrate. Nerol **3** was used as a substrate since control reactions made in previous work[4] showed that caryophyllene alcohol **2** required capsule **I** and HCl for the cyclization to take place.Conditions: 2-methylfuran **4** (10 equiv.), 60°C, 24 h, $CHCl_3$ as solvent. Decane was used as internal standard.

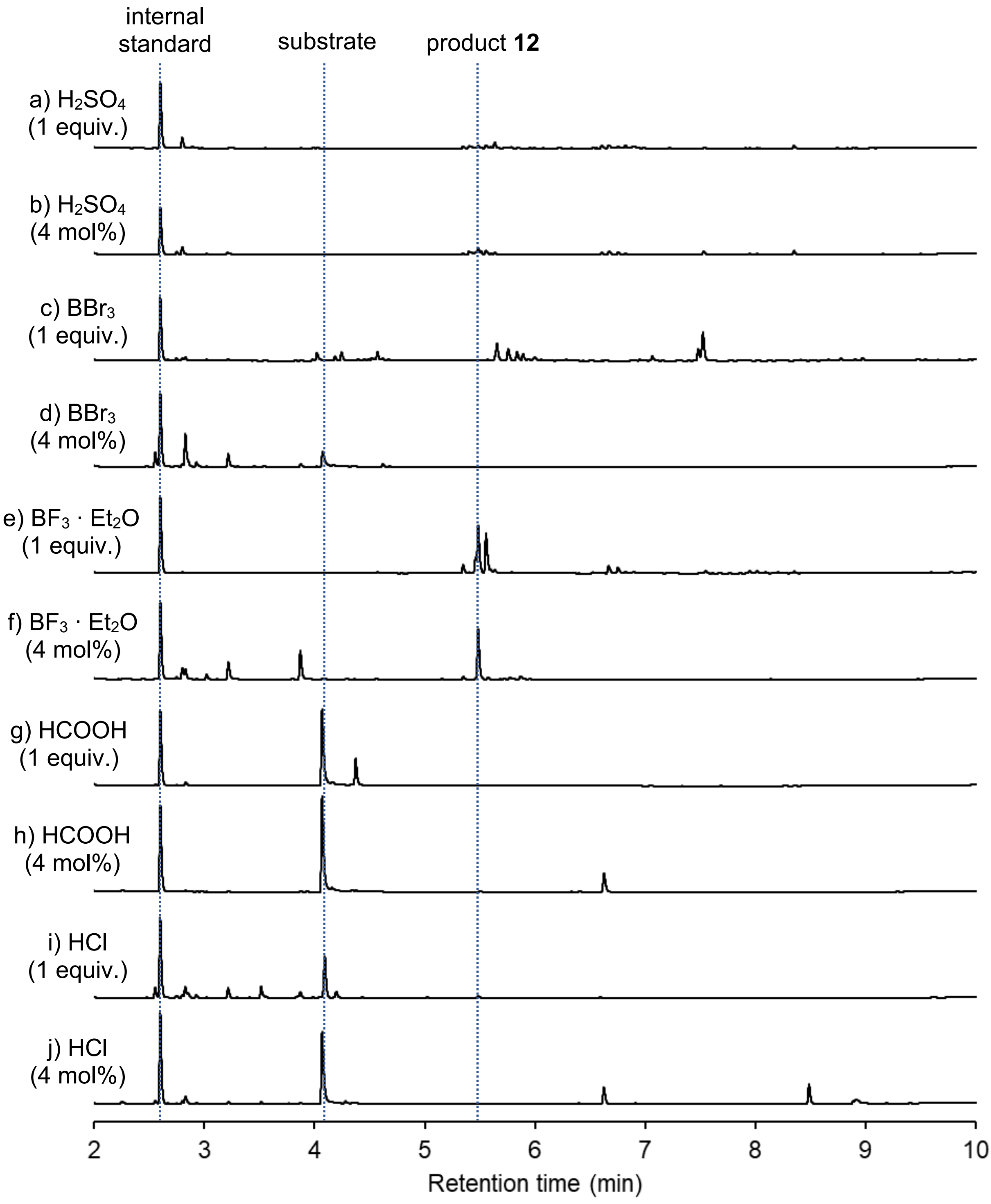

# S9 Cyclization with other nucleophiles

## S9.1 Furan

Reaction of caryophyllene alcohol **2** with furan. Conditions: capsule **I** (10 mol%), HCl (4 mol%), furan (10 equiv.), 60°C, 24 h, $CHCl_3$ as solvent. a: elimination product means that the intermediate carbocation **2b** could not be trapped by a nucleophile and instead eliminated an $H^+$ to give alkenes.

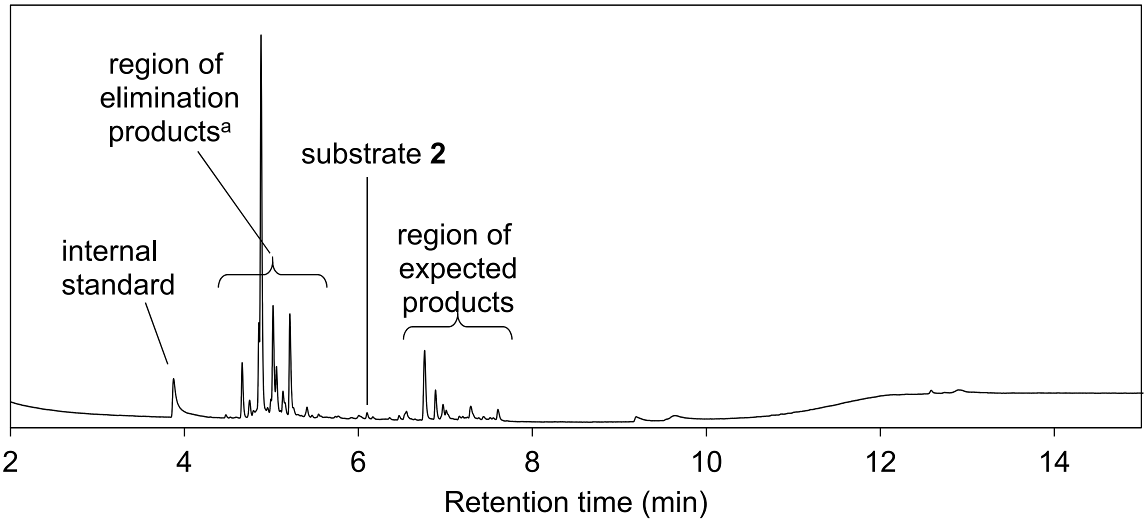


## S9.2 2-methylthiophene

Reaction of caryophyllene alcohol **2** with 2-methylthiophene. Conditions: capsule I (10 mol%), HCl (4 mol%), 2-methylthiophene (10 equiv.), 60°C, 24 h, $CHCl_3$ as solvent. a: elimination product means that the intermediate carbocation **2b** could not be trapped by a nucleophile and instead eliminated an $H^+$ to give alkenes.

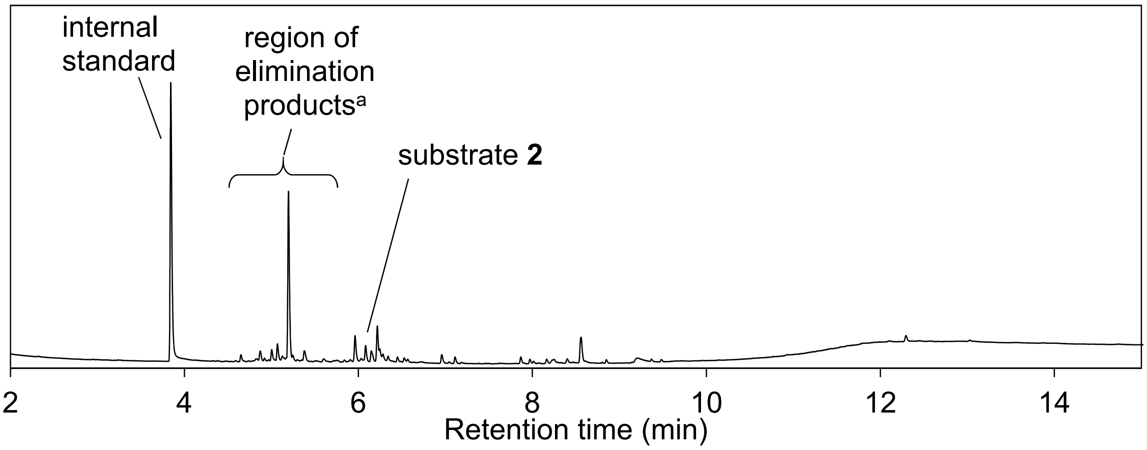

## S9.3 *m*-xylene

Reaction of nerol **3** with *m*-xylene. Conditions: capsule **I** (10 mol%), HCl (4 mol%), 60°C, 24 h, *m*-xylene as solvent.

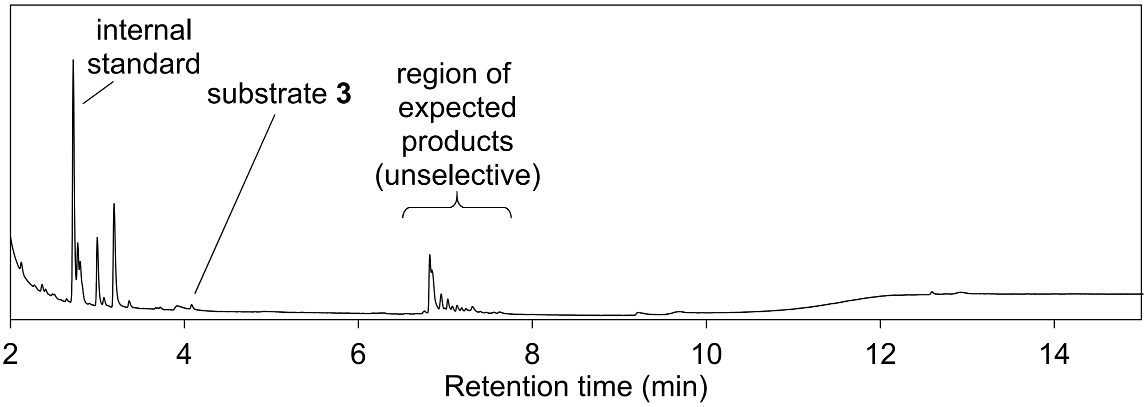


## S9.4 Other unseccessulf nucleophiles

Reaction of caryophyllene alcohol **2** with the following nulceophiles afforded only elimination products (the intermediate carbocation **2b** could not be trapped by a nucleophile and instead eliminated an $H^+$ to give alkenes). Conditions: capsule I (10 mol%), HCl (4 mol%), nucleophile (10 equiv.), 60°C, 24 h, $CHCl_3$ as solvent.

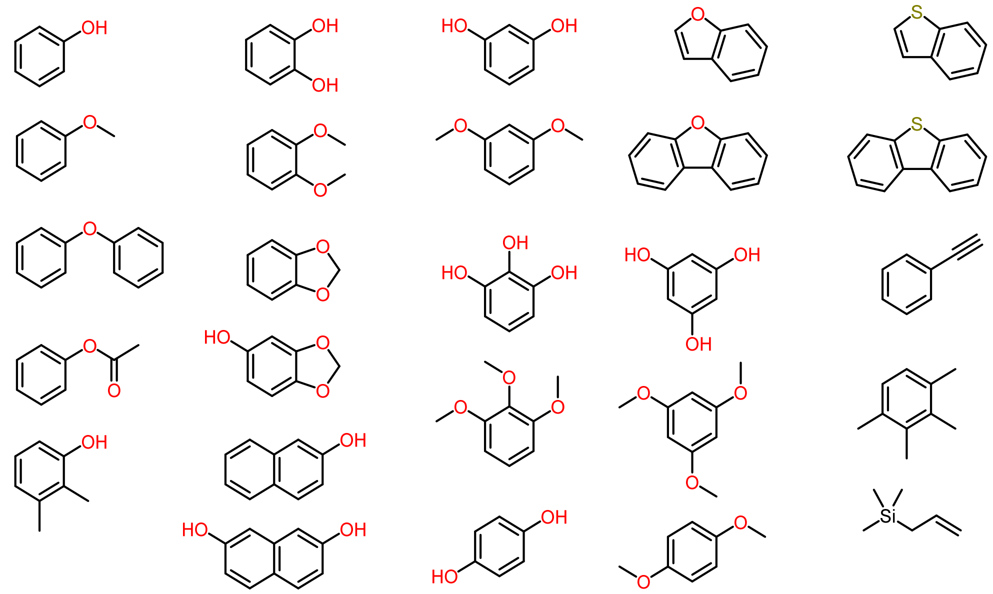

# S10 Cyclization with other electrophiles

The standard reaction conditions were tested with different terpene substrates. Both (*E*,*E*)-farnesol and (*E*,*E*)-farnesyl acetate gave elimination products instead of capture of the 2-methylfuran. Geranyl acetate showed the same product formation as nerol, albeit in lower yield (35% vs 56% GC yield). Conditions: capsule I (10 mol%), HCl (4 mol%), 2-methylfuran (10 equiv.), 60°C, 24 h, $CHCl_3$ as solvent.

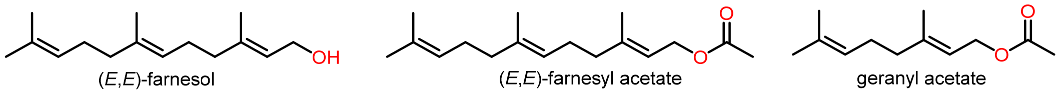

# S11 Nucleophile encapsulation study

$^1$H NMR were recorded for the nucleophiles and caryophyllene alcohol **2**, in the presence of the capsule. Due to the slow interconversion of conformers, leading to very broad signlas, the 1 H NMR spectra had to be measured at 60 °C.

Condition: 333 K, $CDCl_3$, 1 equiv. substrate **2**, 10 mol% capsule **I**, 10 equiv. nucleophile.

Although the signals are generally broad and poorly defined—likely due to the reduced conformational freedom of the entrapped guest molecules—the following conclusion can be drawn: coencapsulation of substrate 2 and the aromatic nucleophiles is observed (approximately 0.5 to –2 ppm). The signals differ from those of the individually encapsulated guests, indicating true coencapsulation. An exception is resorcinol, which showed poor solubility under these conditions.

In conclusion, encapsulation and coencapsulation do not limit the observed reactivity.

Zoom on the region of interest

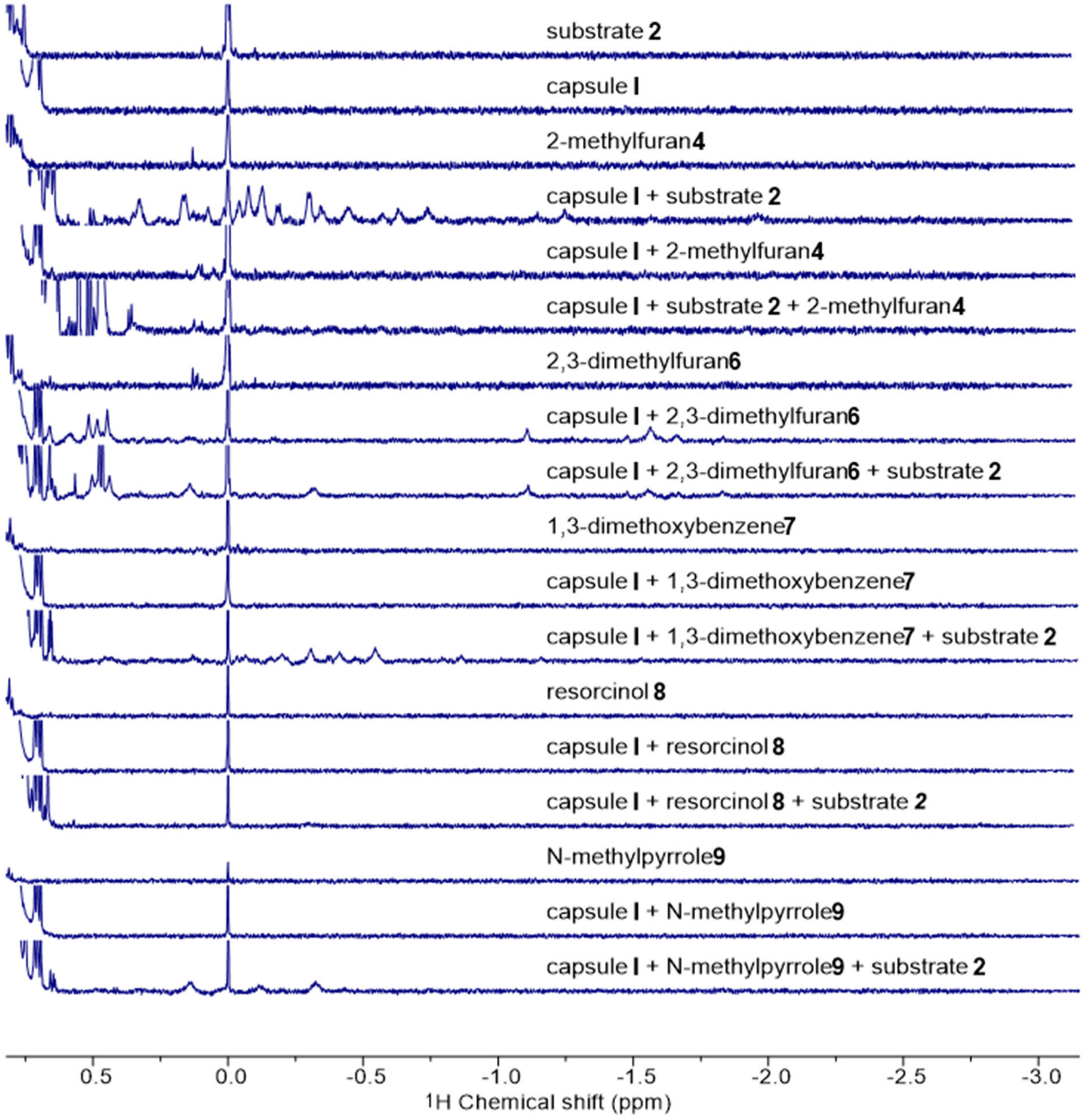

## S12 References

(1) Armarego, W. L. F. *Purification of Laboratory Chemicals*; Butterworth-Heinemann: Amsterdam, 2017.
(2) Scanlon, J. T.; Willis, D. E. Calculation of Flame Ionization Detector Relative Response Factors Using the Effective Carbon Number Concept. *J. Chromatogr. Sci.* **1985**, *23* (8), 333–340. https://doi.org/10.1093/chromsci/23.8.333.
(3) Cornu, I.; Syntrivanis, L.-D.; Tiefenbacher, K. Biomimetic Tail-to-Head Terpene Cyclizations Using the Resorcin[4]Arene Capsule Catalyst. *Nat. Protoc.* **2024**, *19* (2), 313–339. https://doi.org/10.1038/s41596-023-00919-3.
(4) Syntrivanis, L.-D.; Némethová, I.; Schmid, D.; Levi, S.; Prescimone, A.; Bissegger, F.; Major, D. T.; Tiefenbacher, K. Four-Step Access to the Sesquiterpene Natural Product Presilphiperfolan-1β-Ol and Unnatural Derivatives via Supramolecular Catalysis. *J. Am. Chem. Soc.* **2020**, *142* (12), 5894–5900. https://doi.org/10.1021/jacs.0c01464.

# S13 Crystallographic details for compound 18

## COI478_150K

$R_1$=2.93%

### Crystal Data and Experimental

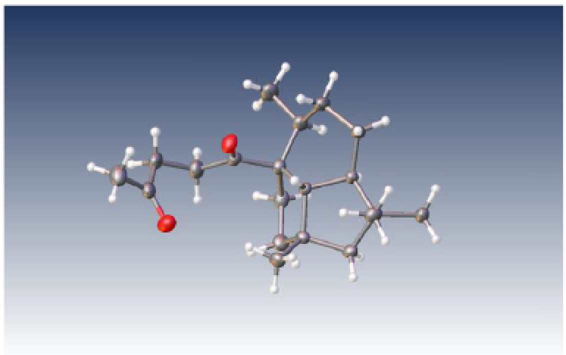

**Experimental**. Single colourless block-shaped crystals of COI478_150K were used as supplied. A suitable crystal with dimensions 0.16 × 0.15 × 0.15 mm³ was selected and mounted on a STOE STADIVARI Cu diffractometer. The crystal was kept at a steady $T$ = 150 K during data collection. The structure was solved with the ShelXT 2018/2 (Sheldrick, 2018) solution program using dual methods and by using Olex2 1.5 (Dolomanov et al., 2009) as the graphical interface. The model was refined with ShelXL 2018/3 (Sheldrick, 2015) using full matrix least squares minimisation on $F^2$.

**Crystal Data**. $C_{20}H_{32}O_2$, $M_r$ = 304.45, orthorhombic, $P2_12_12_1$ (No. 19), a = 6.16230(10) Å, b = 9.2561(2) Å, c = 31.3315(7) Å, $\alpha = \beta = \gamma = 90°$, $V$ = 1787.11(6) Å³, $T$ = 150 K, $Z$ = 4, $Z'$ = 1, $\mu$(Cu K$_\alpha$) = 0.543, 44832 reflections measured, 3337 unique ($R_{int}$ = 0.0236) which were used in all calculations. The final $wR_2$ was 0.0757 (all data) and $R_1$ was 0.0293 (I≥2 $\sigma$(I)).

| Compound | |
|---|---|
| Formula | $C_{20}H_{32}O_2$ |
| $D_{calc.}$/ g cm$^{-3}$ | 1.132 |
| $\mu$/mm$^{-1}$ | 0.543 |
| Formula Weight | 304.45 |
| Colour | colourless |
| Shape | block-shaped |
| Size/mm³ | 0.16×0.15×0.15 |
| $T$/K | 150 |
| Crystal System | orthorhombic |
| Flack Parameter | 0.07(7) |
| Space Group | $P2_12_12_1$ |
| $a$/Å | 6.16230(10) |
| $b$/Å | 9.2561(2) |
| $c$/Å | 31.3315(7) |
| $\alpha$/° | 90 |
| $\beta$/° | 90 |
| $\gamma$/° | 90 |
| V/Å³ | 1787.11(6) |
| $Z$ | 4 |
| $Z'$ | 1 |
| Wavelength/Å | 1.54186 |
| Radiation type | Cu K$_\alpha$ |
| $\Theta_{min}$/° | 4.982 |
| $\Theta_{max}$/° | 69.837 |
| Measured Refl's. | 44832 |
| Indep't Refl's | 3337 |
| Refl's I≥2 $\sigma$(I) | 3082 |
| $R_{int}$ | 0.0236 |
| Parameters | 205 |
| Restraints | 0 |
| Largest Peak | 0.181 |
| Deepest Hole | -0.115 |
| GooF | 1.029 |
| $wR_2$ (all data) | 0.0757 |
| $wR_2$ | 0.0731 |
| $R_1$ (all data) | 0.0338 |
| $R_1$ | 0.0293 |

## Structure Quality Indicators

**Reflections:**

| | | | |
|---|---|---|---|
| d min (CuKα) 2Θ=139.7° 0.82 | I/σ(I) 81.7 | Rint m=13.43 2.36% | Full 135.4° 99% to 139.7° 99.6 |

**Refinement:**

| | | | |
|---|---|---|---|
| Shift n/a | Max Peak n/a | Min Peak n/a | GooF n/a |

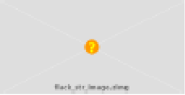

A colourless block-shaped-shaped crystal with dimensions 0.16 × 0.15 × 0.15 $mm^3$ was mounted. Data were collected using a STOE STADIVARI Cu diffractometer operating at $T$ = 150 K.

Data were measured using rotation method, $\omega$ scans with Cu $K_\alpha$ radiation. The diffraction pattern was indexed and the total number of runs and images was based on the strategy calculation from the program X-Area Pilatus3_SV 1.31.192.0 (STOE, 2023). The maximum resolution that was achieved was $\Theta$ = 69.837° (0.82 Å).

The unit cell was refined using X-Area Integrate 2.5.15.0 (STOE, 2023)X-Area LANA 2.7.12 (STOE, 2023) on 62270 reflections, 139% of the observed reflections.

Data reduction, scaling and absorption corrections were performed using X-Area Integrate 2.5.15.0 (STOE, 2023)X-Area LANA 2.7.12 (STOE, 2023). The final completeness is 99.40 % out to 69.837° in $\Theta$. A multi-scan absorption correction was performed using STOE X-Red32, absorption correction by Gaussian integration, analogous to P. Coppens in: F. R. Ahmed (Editor), "Crystallographic Computing", Munksgaard, Copenhagen (1970), 255 - 270. Afterwards scaling of reflection intensities was performed within STOE LANA. J. Koziskova, F. Hahn, J. Richter, J. Kozisek, Acta Chimica Slovaca, vol. 9, no. 2, 2016, pp. 136 - 140. Finally a spherical absorption correction was done within STOE LANA.. The absorption coefficient $\mu$ of this material is 0.543 $mm^{-1}$ at this wavelength ($\lambda$ = 1.54186Å) and the minimum and maximum transmissions are 0.497 and 0.754.

The structure was solved and the space group $P2_12_12_1$ (# 19) determined by the ShelXT 2018/2 (Sheldrick, 2018) structure solution program using using dual methods and refined by full matrix least squares minimisation on $F^2$ using version 2018/3 of ShelXL 2018/3 (Sheldrick, 2015). All non-hydrogen atoms were refined anisotropically. Hydrogen atom positions were calculated geometrically and refined using the riding model. Hydrogen atom positions were calculated geometrically and refined using the riding model.

*_exptl_absorpt_process_details*: STOE X-Red32, absorption correction by Gaussian integration, analogous toP. Coppens in: F. R. Ahmed (Editor), "Crystallographic Computing", Munksgaard,Copenhagen (1970), 255 - 270. Afterwards scaling of reflection intensities wasperformed within STOE LANA. J. Koziskova, F. Hahn, J. Richter, J. Kozisek, ActaChimica Slovaca, vol. 9, no. 2, 2016, pp. 136 - 140.Finally a spherical absorption correction was done within STOE LANA.

There is a single formula unit in the asymmetric unit, which is represented by the reported sum formula. In other words: Z is 4 and Z' is 1. The moiety formula is C20 H32 O2.

The Flack parameter was refined to 0.07(7). Determination of absolute structure using Bayesian statistics on Bijvoet differences using the Olex2 results in None. This structure is in chiral space group, but there are no chiral atoms. Note: The Flack parameter is used to determine chirality of the crystal studied, the value should be near 0, a value of 1 means that the stereochemistry is wrong and the model should be inverted. A value of 0.5 means that the crystal consists of a racemic mixture of the two enantiomers.

## Data Plots: Diffraction Data

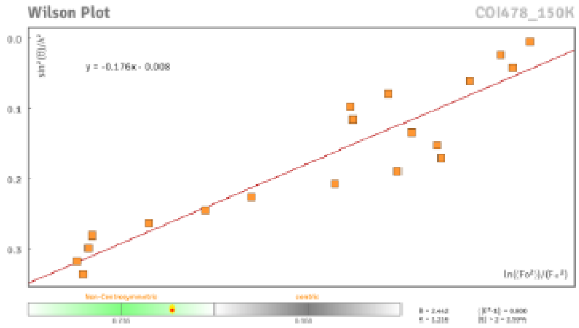


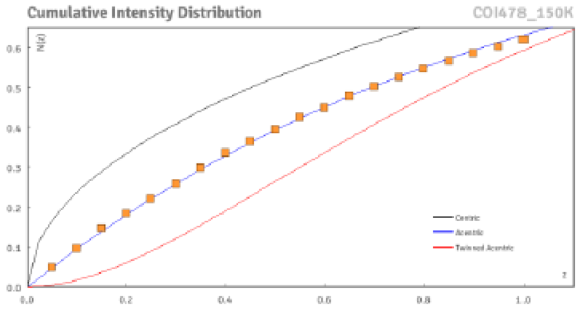


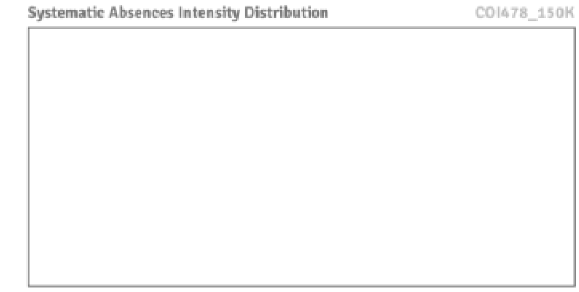


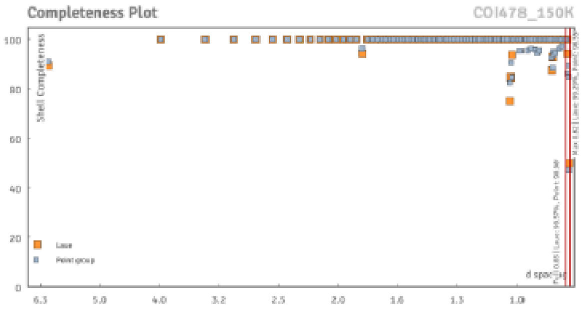


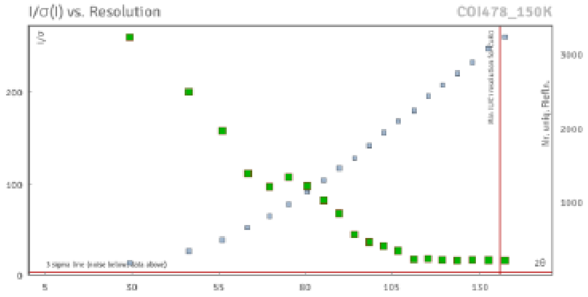


## Data Plots: Refinement and Data

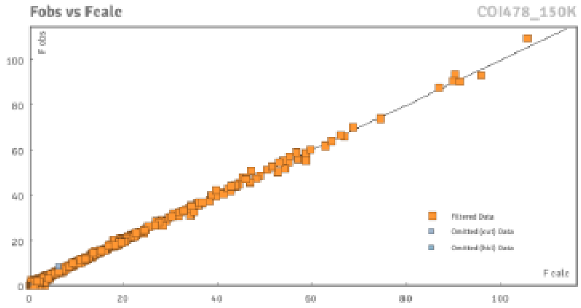


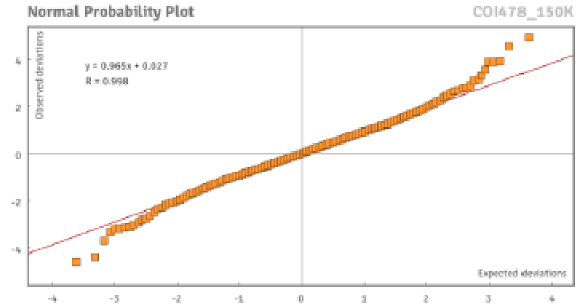


## Reflection Statistics

| | | | |
|---|---|---|---|
| Total reflections (after filtering) | 44832 | Unique reflections | 3337 |
| Completeness | 0.986 | Mean I/$\sigma$ | 72.3 |
| $hkl_{max}$ collected | (4, 11, 35) | $hkl_{min}$ collected | (-7, -11, -38) |
| $hkl_{max}$ used | (7, 11, 38) | $hkl_{min}$ used | (-7, 0, 0) |
| Lim $d_{max}$ collected | 100.0 | Lim $d_{min}$ collected | 0.77 |
| $d_{max}$ used | 8.88 | $d_{min}$ used | 0.82 |
| Friedel pairs | 1799 | Friedel pairs merged | 0 |
| Inconsistent equivalents | 17 | $R_{int}$ | 0.0236 |
| $R_{sigma}$ | 0.0122 | Intensity transformed | 0 |

| | | | |
|---|---|---|---|
| Omitted reflections | 14 | Omitted by user (OMIT hkl) | 0 |
| Multiplicity | (1566, 1090, 713, 594, 558, 525, 460, 476, 451, 366, 258, 213, 199, 161, 115, 93, 55, 40, 26, 14, 1) | Maximum multiplicity | 45 |
| Removed systematic absences | 0 | Filtered off (Shel/OMIT) | 0 |

**Table 1**: Fractional Atomic Coordinates (×10[4]) and Equivalent Isotropic Displacement Parameters (Å$^2$×10$^3$) for COI478_150K. $U_{eq}$ is defined as 1/3 of the trace of the orthogonalised $U_{ij}$.

| Atom | x | y | z | $U_{eq}$ |
|---|---|---|---|---|
| O2 | 8065(2) | 7107.9(14) | 6755.2(4) | 33.9(3) |
| O1 | 7814(3) | 4405.4(14) | 7409.8(4) | 46.7(4) |
| C9 | 6913(3) | 4631.9(16) | 5299.5(5) | 22.5(3) |
| C7 | 6615(3) | 5609.8(16) | 6013.6(4) | 19.6(3) |
| C8 | 6256(3) | 6002.4(16) | 5545.7(5) | 21.5(3) |
| C5 | 6180(3) | 6738.9(17) | 6735.0(5) | 25.0(4) |
| C11 | 5986(3) | 3991.9(16) | 6054.2(5) | 21.5(3) |
| C10 | 5761(3) | 3487.6(17) | 5581.6(5) | 23.7(3) |
| C6 | 5094(3) | 6517.4(18) | 6298.8(5) | 22.6(3) |
| C1 | 7992(3) | 5568.8(19) | 7583.6(5) | 33.3(4) |
| C17 | 7292(3) | 7467.7(17) | 5459.4(5) | 29.2(4) |
| C13 | 3026(3) | 5583.5(19) | 6309.7(5) | 28.5(4) |
| C14 | 7681(3) | 3083.7(18) | 6291.9(5) | 30.5(4) |
| C16 | 9365(3) | 4406.7(19) | 5287.3(5) | 28.3(4) |
| C18 | 6709(3) | 8516.7(18) | 5830.4(5) | 32.1(4) |
| C19 | 4715(3) | 8035.9(19) | 6086.3(5) | 29.0(4) |
| C4 | 4885(3) | 6519(2) | 7141.3(5) | 33.0(4) |
| C15 | 6043(3) | 4648.2(18) | 4840.8(5) | 30.7(4) |
| C3 | 6283(4) | 6719(2) | 7536.7(5) | 36.4(5) |
| C12 | 3837(3) | 4015.0(18) | 6311.7(5) | 29.4(4) |
| C20 | 3960(4) | 9203(2) | 6398.1(6) | 41.8(5) |
| C2 | 9888(4) | 5927(3) | 7863.3(6) | 45.6(5) |

**Table 2**: Anisotropic Displacement Parameters (×10[4]) for COI478_150K. The anisotropic displacement factor exponent takes the form: $-2\pi^2[h^2a^{*2} \times U_{11} + ... + 2hka^* \times b^* \times U_{12}]$

| Atom | $U_{11}$ | $U_{22}$ | $U_{33}$ | $U_{23}$ | $U_{13}$ | $U_{12}$ |
|---|---|---|---|---|---|---|
| O2 | 32.6(8) | 41.4(7) | 27.8(6) | -7.3(5) | -2.4(5) | -6.5(6) |
| O1 | 69.6(10) | 31.1(7) | 39.3(7) | -3.9(5) | -14.5(7) | 1.8(7) |
| C9 | 28.3(9) | 18.7(7) | 20.4(7) | -2.2(6) | -1.8(7) | 0.7(7) |
| C7 | 19.7(8) | 19.0(7) | 20.1(7) | -1.1(6) | -1.2(6) | -0.1(6) |
| C8 | 25.7(9) | 18.4(7) | 20.3(7) | -0.4(6) | -0.5(6) | 1.6(7) |
| C5 | 30.0(10) | 21.8(8) | 23.4(7) | -2.6(6) | -0.1(7) | 1.8(7) |
| C11 | 22.8(9) | 19.3(7) | 22.5(7) | 2.6(6) | -1.2(6) | -2.5(7) |
| C10 | 25.4(9) | 19.2(7) | 26.5(8) | -1.8(6) | -4.4(7) | -1.8(7) |
| C6 | 23.2(9) | 23.9(7) | 20.7(7) | -1.4(6) | 0.8(6) | 1.0(7) |
| C1 | 46.7(12) | 34.0(9) | 19.2(7) | 3.2(7) | 2.0(7) | -6.8(9) |
| C17 | 44.4(12) | 18.8(7) | 24.3(8) | 0.3(6) | 4.7(7) | -0.8(7) |
| C13 | 22.2(9) | 37.0(9) | 26.3(7) | -2.5(7) | 0.2(7) | 0.7(8) |
| C14 | 37.3(11) | 24.2(8) | 30.1(8) | 4.6(7) | -8.4(7) | 2.3(8) |
| C16 | 29.4(10) | 26.5(8) | 29.1(8) | -4.3(7) | 4.0(7) | 1.0(7) |
| C18 | 49.8(12) | 18.5(7) | 28.1(8) | -1.9(6) | 4.7(8) | 0.3(8) |
| C19 | 36.6(10) | 24.2(8) | 26.1(8) | -1.3(6) | -0.1(7) | 8.1(7) |
| C4 | 38.3(11) | 37.9(9) | 22.7(8) | -0.9(7) | 4.4(7) | 2.8(9) |
| C15 | 45.2(12) | 24.8(8) | 22.1(8) | -1.9(6) | -3.9(7) | 2.8(8) |
| C3 | 53.1(13) | 35.7(10) | 20.2(7) | -4.6(7) | 3.3(8) | -2.3(9) |
| C12 | 27.9(10) | 30.9(9) | 29.4(8) | 1.8(7) | 4.2(7) | -6.5(8) |
| C20 | 59.6(14) | 30.3(9) | 35.4(9) | -3.6(8) | 7.2(9) | 15.2(10) |
| C2 | 46.4(13) | 55.1(12) | 35.3(9) | 1.2(9) | -2.7(9) | -13.5(11) |

**Table 3**: Bond Lengths in Å for COI478_150K.

| Atom | Atom | Length/Å | Atom | Atom | Length/Å |
|---|---|---|---|---|---|
| O2 | C5 | 1.213(2) | C11 | C10 | 1.559(2) |
| O1 | C1 | 1.212(2) | C11 | C14 | 1.534(2) |
| C9 | C8 | 1.539(2) | C11 | C12 | 1.551(2) |
| C9 | C10 | 1.551(2) | C6 | C13 | 1.540(2) |
| C9 | C16 | 1.525(3) | C6 | C19 | 1.573(2) |
| C9 | C15 | 1.534(2) | C1 | C3 | 1.505(3) |
| C7 | C8 | 1.5263(19) | C1 | C2 | 1.497(3) |
| C7 | C11 | 1.552(2) | C17 | C18 | 1.557(2) |
| C7 | C6 | 1.544(2) | C13 | C12 | 1.535(2) |
| C8 | C17 | 1.523(2) | C18 | C19 | 1.533(3) |
| C5 | C6 | 1.535(2) | C19 | C20 | 1.529(2) |
| C5 | C4 | 1.516(2) | C4 | C3 | 1.520(3) |

**Table 4**: Bond Angles in ° for COI478_150K.

| Atom | Atom | Atom | Angle/° | Atom | Atom | Atom | Angle/° |
|---|---|---|---|---|---|---|---|
| C8 | C9 | C10 | 99.02(12) | C12 | C11 | C10 | 114.99(13) |
| C16 | C9 | C8 | 112.71(14) | C9 | C10 | C11 | 107.23(12) |
| C16 | C9 | C10 | 111.97(14) | C7 | C6 | C19 | 109.35(12) |
| C16 | C9 | C15 | 108.90(14) | C5 | C6 | C7 | 108.88(13) |
| C15 | C9 | C8 | 111.68(13) | C5 | C6 | C13 | 114.57(13) |
| C15 | C9 | C10 | 112.37(14) | C5 | C6 | C19 | 108.80(13) |
| C8 | C7 | C11 | 105.81(11) | C13 | C6 | C7 | 102.10(13) |
| C8 | C7 | C6 | 109.79(12) | C13 | C6 | C19 | 112.83(14) |
| C6 | C7 | C11 | 109.03(13) | O1 | C1 | C3 | 121.41(18) |
| C7 | C8 | C9 | 104.30(12) | O1 | C1 | C2 | 122.04(19) |
| C17 | C8 | C9 | 122.31(14) | C2 | C1 | C3 | 116.53(16) |
| C17 | C8 | C7 | 108.77(12) | C8 | C17 | C18 | 109.03(13) |
| O2 | C5 | C6 | 120.11(15) | C12 | C13 | C6 | 105.15(14) |
| O2 | C5 | C4 | 119.89(15) | C19 | C18 | C17 | 113.23(15) |
| C4 | C5 | C6 | 120.00(15) | C18 | C19 | C6 | 111.22(14) |
| C7 | C11 | C10 | 103.48(11) | C20 | C19 | C6 | 113.95(14) |
| C14 | C11 | C7 | 113.50(14) | C20 | C19 | C18 | 111.91(16) |
| C14 | C11 | C10 | 110.96(13) | C5 | C4 | C3 | 111.67(16) |
| C14 | C11 | C12 | 109.66(13) | C1 | C3 | C4 | 112.95(15) |
| C12 | C11 | C7 | 104.03(13) | C13 | C12 | C11 | 106.80(13) |

**Table 5**: Torsion Angles in ° for COI478_150K.

| Atom | Atom | Atom | Atom | Angle/° |
|---|---|---|---|---|
| O2 | C5 | C6 | C7 | 46.8(2) |
| O2 | C5 | C6 | C13 | 160.37(15) |
| O2 | C5 | C6 | C19 | -72.3(2) |
| O2 | C5 | C4 | C3 | -3.7(2) |
| O1 | C1 | C3 | C4 | -21.8(2) |
| C9 | C8 | C17 | C18 | -165.92(15) |
| C7 | C8 | C17 | C18 | -44.35(19) |
| C7 | C11 | C10 | C9 | 16.90(17) |
| C7 | C11 | C12 | C13 | 13.93(16) |
| C7 | C6 | C13 | C12 | 35.89(15) |
| C7 | C6 | C19 | C18 | -31.34(18) |
| C7 | C6 | C19 | C20 | -158.96(16) |
| C8 | C9 | C10 | C11 | -37.74(16) |
| C8 | C7 | C11 | C10 | 11.35(17) |
| C8 | C7 | C11 | C14 | 131.70(14) |
| C8 | C7 | C11 | C12 | -109.17(14) |
| C8 | C7 | C6 | C5 | -150.73(13) |
| C8 | C7 | C6 | C13 | 87.75(15) |

| Atom | Atom | Atom | Atom | Angle/˚ |
|---|---|---|---|---|
| C8 | C7 | C6 | C19 | -31.97(18) |
| C8 | C17 | C18 | C19 | -20.1(2) |
| C5 | C6 | C13 | C12 | -81.62(16) |
| C5 | C6 | C19 | C18 | 87.46(16) |
| C5 | C6 | C19 | C20 | -40.1(2) |
| C5 | C4 | C3 | C1 | -66.9(2) |
| C11 | C7 | C8 | C9 | -35.86(17) |
| C11 | C7 | C8 | C17 | -167.86(14) |
| C11 | C7 | C6 | C5 | 93.78(15) |
| C11 | C7 | C6 | C13 | -27.73(15) |
| C11 | C7 | C6 | C19 | -147.46(14) |
| C10 | C9 | C8 | C7 | 44.55(15) |
| C10 | C9 | C8 | C17 | 168.20(15) |
| C10 | C11 | C12 | C13 | -98.50(15) |
| C6 | C7 | C8 | C9 | -153.37(13) |
| C6 | C7 | C8 | C17 | 74.62(17) |
| C6 | C7 | C11 | C10 | 129.37(13) |
| C6 | C7 | C11 | C14 | -110.28(15) |
| C6 | C7 | C11 | C12 | 8.86(15) |
| C6 | C5 | C4 | C3 | 177.22(15) |
| C6 | C13 | C12 | C11 | -31.75(16) |
| C17 | C18 | C19 | C6 | 60.78(19) |
| C17 | C18 | C19 | C20 | -170.51(16) |
| C13 | C6 | C19 | C18 | -144.23(14) |
| C13 | C6 | C19 | C20 | 88.16(19) |
| C14 | C11 | C10 | C9 | -105.16(16) |
| C14 | C11 | C12 | C13 | 135.65(14) |
| C16 | C9 | C8 | C7 | -73.94(16) |
| C16 | C9 | C8 | C17 | 49.7(2) |
| C16 | C9 | C10 | C11 | 81.31(16) |
| C19 | C6 | C13 | C12 | 153.14(13) |
| C4 | C5 | C6 | C7 | -134.14(15) |
| C4 | C5 | C6 | C13 | -20.6(2) |
| C4 | C5 | C6 | C19 | 106.76(17) |
| C15 | C9 | C8 | C7 | 163.09(14) |
| C15 | C9 | C8 | C17 | -73.3(2) |
| C15 | C9 | C10 | C11 | -155.76(14) |
| C12 | C11 | C10 | C9 | 129.66(15) |
| C2 | C1 | C3 | C4 | 159.53(16) |

**Table 6**: Hydrogen Fractional Atomic Coordinates (×10⁴) and Equivalent Isotropic Displacement Parameters (Å$^2$×10$^3$) for COI478_150K. $U_{eq}$ is defined as 1/3 of the trace of the orthogonalised $U_{ij}$.

| Atom | x | y | z | $U_{eq}$ |
|---|---|---|---|---|
| H7 | 8165.34 | 5762.22 | 6096.14 | 24 |
| H8 | 4656.08 | 6124.67 | 5505.76 | 26 |
| H10A | 6447.86 | 2529.49 | 5542.95 | 28 |
| H10B | 4211 | 3411.62 | 5501.93 | 28 |
| H17A | 6749.97 | 7861 | 5185.56 | 35 |
| H17B | 8886.78 | 7362.12 | 5438.56 | 35 |
| H13A | 2164.08 | 5789.84 | 6569.31 | 34 |
| H13B | 2113.72 | 5770.8 | 6055.56 | 34 |
| H14A | 9014.33 | 3033.35 | 6122.08 | 46 |
| H14B | 7114.26 | 2106.32 | 6337.09 | 46 |
| H14C | 7997.65 | 3531.05 | 6568.49 | 46 |
| H16A | 10036.07 | 5170.79 | 5115.67 | 42 |
| H16B | 9688.07 | 3463.99 | 5159.9 | 42 |
| H16C | 9944.6 | 4440.74 | 5578.32 | 42 |
| H18A | 7966.47 | 8591.28 | 6025.72 | 39 |
| H18B | 6432.91 | 9489.53 | 5710.83 | 39 |
| H19 | 3513.69 | 7905.8 | 5875.2 | 35 |
| H4A | 4261.56 | 5532.77 | 7142.15 | 40 |

| Atom | x | y | z | $U_{eq}$ |
|---|---|---|---|---|
| H4B | 3668.34 | 7216.95 | 7148.71 | 40 |
| H15A | 4455.58 | 4706.05 | 4846.22 | 46 |
| H15B | 6488.53 | 3761.38 | 4694.12 | 46 |
| H15C | 6629.94 | 5487.68 | 4689.11 | 46 |
| H3A | 6998.17 | 7675.84 | 7522.72 | 44 |
| H3B | 5339.52 | 6709.53 | 7792.45 | 44 |
| H12A | 2751.48 | 3370.49 | 6177.59 | 35 |
| H12B | 4092.19 | 3684.15 | 6607.97 | 35 |
| H20A | 2669.12 | 8863.93 | 6551.01 | 63 |
| H20B | 3605.95 | 10085.23 | 6239.94 | 63 |
| H20C | 5120.28 | 9407 | 6603.31 | 63 |
| H2A | 10763.19 | 5056.39 | 7909.08 | 68 |
| H2B | 9363.09 | 6287.92 | 8138.63 | 68 |
| H2C | 10777.24 | 6669.55 | 7725.32 | 68 |

**Citations**

O.V. Dolomanov and L.J. Bourhis and R.J. Gildea and J.A.K. Howard and H. Puschmann, Olex2: A complete structure solution, refinement and analysis program, *J. Appl. Cryst.*, (2009), **42**, 339-341.

Sheldrick, G.M., Crystal structure refinement with ShelXL, *Acta Cryst.*, (2015), **C71**, 3-8.

Sheldrick, G.M., ShelXT-Integrated space-group and crystal-structure determination, *Acta Cryst.*, (2015), **A71**, 3-8.

X-Area Integrate 2.5.15.0 (STOE, 2023) X-Area LANA 2.7.12 (STOE, 2023)

X-Area Pilatus3_SV 1.31.192.0 (STOE, 2023)

X-Area Recipe 2.0.1 (STOE, 2023)

# S14 $^{13}$C chemical shift calculations

NMR-predictions were calculated with Spartan ’20 (Wavefunction, Inc.) using DFT-ωB97X-D with basis set 6-31G* (ωB97X-V/6-311+G(2DF,2P)[6-311G*] Weighted Average) with ωB97X-D/6-31G* geometry.

| 2-(presilphiperfolanyl)-5-methylfuran **10** | | |
|---|---|---|
| calculated | measured | abs. difference |
| 157.4 | 157.8 | 0.4 |
| 147.9 | 148.9 | 1.0 |
| 107.9 | 105.0 | 2.9 |
| 106.9 | 104.3 | 2.6 |
| 61.2 | 60.1 | 1.1 |
| 58.1 | 58.6 | 0.5 |
| 50.9 | 50.4 | 0.5 |
| 47.5 | 47.4 | 0.1 |
| 44.2 | 44.5 | 0.3 |
| 42.1 | 41.7 | 0.4 |
| 39.5 | 39.1 | 0.4 |
| 39.2 | 37.6 | 1.6 |
| 37.1 | 35.6 | 1.5 |
| 31.4 | 30.0 | 1.4 |
| 29.2 | 27.9 | 1.3 |
| 28.4 | 27.6 | 0.8 |
| 22.3 | 21.1 | 1.2 |
| 22.2 | 19.8 | 2.4 |
| 18.3 | 16.9 | 1.4 |
| 13.7 | 12.9 | 0.8 |

| 2-(presilphiperfolanyl)-5-methylfuran **10b** | | |
|---|---|---|
| calculated | measured | abs. difference |
| 157.3 | 157.8 | 0.5 |
| 147.8 | 148.9 | 1.1 |
| 108.1 | 105.0 | 3.1 |
| 107.6 | 104.3 | 3.3 |
| 61.6 | 60.1 | 1.5 |
| 58.6 | 58.6 | 0.0 |
| 51.7 | 50.4 | 1.3 |
| 51.3 | 47.4 | 3.9 |
| 44.8 | 44.5 | 0.3 |
| 41.9 | 41.7 | 0.2 |
| 39.5 | 39.1 | 0.4 |
| 36.6 | 37.6 | 1.0 |
| 32.9 | 35.6 | 2.7 |
| 30.7 | 30.0 | 0.7 |
| 29.0 | 27.9 | 1.1 |
| 28.4 | 27.6 | 0.8 |
| 24.9 | 21.1 | 3.8 |
| 22.1 | 19.8 | 2.3 |
| 17.4 | 16.9 | 0.5 |
| 13.6 | 12.9 | 0.7 |

| 2-(presilphiperfolanyl)-4,5-dimethylfuran **11** | | |
|---|---|---|
| calculated | measured | abs. difference |
| 154.6 | 157.3 | 2.7 |
| 144.1 | 144.9 | 0.8 |
| 116.6 | 113.5 | 3.1 |
| 110.1 | 108.7 | 1.4 |
| 60.4 | 61.0 | 0.6 |
| 57.8 | 59.5 | 1.7 |
| 51.4 | 51.2 | 0.2 |
| 47.3 | 48.4 | 1.1 |
| 44.2 | 45.7 | 1.5 |
| 41.8 | 42.6 | 0.8 |
| 39.3 | 40.0 | 0.7 |
| 38.9 | 38.5 | 0.4 |
| 36.9 | 36.5 | 0.4 |
| 31.9 | 31.0 | 0.9 |
| 28.4 | 28.8 | 0.4 |
| 28.3 | 28.6 | 0.3 |
| 22.4 | 22.1 | 0.3 |
| 22.4 | 20.8 | 1.6 |
| 18.3 | 17.9 | 0.4 |
| 11.2 | 11.6 | 0.4 |
| 10.7 | 10.2 | 0.5 |

| 2-(trans-piperityl)-5-methylfuran **12** | | |
|---|---|---|
| calculated | measured | abs. difference |
| 153.9 | 157.3 | 3.4 |
| 148.2 | 150.3 | 2.1 |
| 133.8 | 134.7 | 0.9 |
| 126.6 | 122.6 | 4.0 |
| 108.9 | 105.6 | 3.3 |
| 107.7 | 105.6 | 2.1 |
| 42.5 | 43.5 | 1.0 |
| 40.9 | 39.2 | 1.7 |
| 30.2 | 29.5 | 0.7 |
| 26.7 | 27.6 | 0.9 |
| 23.7 | 23.4 | 0.3 |
| 22.5 | 21.6 | 0.9 |
| 21.3 | 21.3 | 0.0 |
| 15.8 | 17.1 | 1.3 |
| 13.6 | 13.5 | 0.1 |

| 2-(trans-piperityl)-4-methylfuran **13** | | |
|---|---|---|
| calculated | measured | abs. difference |
| 156.7 | 153.2 | 3.5 |
| 136.7 | 139.9 | 3.2 |
| 136.6 | 135.2 | 1.4 |
| 122.0 | 123.2 | 1.2 |
| 120.4 | 114.5 | 5.9 |
| 110.5 | 112.8 | 2.3 |
| 42.3 | 43.6 | 1.3 |
| 38.1 | 37.8 | 0.3 |
| 26.6 | 30.5 | 3.9 |
| 25.7 | 28.2 | 2.5 |
| 23.8 | 23.6 | 0.2 |
| 21.7 | 22.5 | 0.8 |
| 21.1 | 21.4 | 0.3 |
| 20.5 | 16.7 | 3.8 |
| 10.7 | 10.0 | 0.7 |

| 2-(trans-piperityl)-4,5-dimethylfuran **14** | | |
|---|---|---|
| calculated | measured | abs. difference |
| 154.5 | 156.2 | 1.7 |
| 144.3 | 145.4 | 1.1 |
| 136.2 | 134.8 | 1.4 |
| 122.2 | 122.9 | 0.7 |
| 116.5 | 114.2 | 2.3 |
| 110.9 | 108.4 | 2.5 |
| 42.6 | 43.6 | 1.0 |
| 38.0 | 39.4 | 1.4 |
| 26.6 | 29.7 | 3.1 |
| 25.6 | 27.8 | 2.2 |
| 23.8 | 23.7 | 0.1 |
| 21.9 | 21.8 | 0.1 |
| 21.1 | 21.6 | 0.5 |
| 20.4 | 17.3 | 3.1 |
| 11.1 | 11.5 | 0.4 |
| 10.8 | 10.1 | 0.7 |

| 2-(trans-piperityl)-1,5-dimethoxybenzene **15** | | |
|---|---|---|
| calculated | measured | abs. difference |
| 157.5 | 158.9 | 1.4 |
| 157.4 | 158.4 | 1.0 |
| 133.6 | 134.1 | 0.5 |
| 130.5 | 129.3 | 1.2 |
| 129.9 | 127.4 | 2.5 |
| 126.8 | 125.5 | 1.3 |
| 101.6 | 104.2 | 2.6 |
| 98.7 | 98.3 | 0.4 |
| 53.9 | 55.4 | 1.5 |
| 53.9 | 55.4 | 1.5 |
| 48.9 | 46.2 | 2.7 |
| 37.4 | 37.0 | 0.4 |
| 30.7 | 29.8 | 0.9 |
| 26.6 | 27.7 | 1.1 |
| 23.8 | 23.8 | 0.0 |
| 23.1 | 21.9 | 1.2 |
| 21.7 | 21.7 | 0.0 |
| 16.7 | 17.7 | 1.0 |

| cyclic ether **16** | | |
|---|---|---|
| calculated | measured | abs. difference |
| 157.3 | 157.5 | 0.2 |
| 155.2 | 154.9 | 0.3 |
| 130.2 | 128.8 | 1.4 |
| 117.1 | 120.4 | 3.3 |
| 106.2 | 106.5 | 0.3 |
| 100.3 | 102.0 | 1.7 |
| 74.4 | 75.0 | 0.6 |
| 46.9 | 47.4 | 0.5 |
| 34.6 | 35.0 | 0.4 |
| 34.4 | 33.9 | 0.5 |
| 30.1 | 30.9 | 0.8 |
| 29.4 | 29.4 | 0.0 |
| 26.3 | 26.3 | 0.0 |
| 22.3 | 22.1 | 0.2 |
| 20.8 | 21.3 | 0.5 |
| 20.0 | 19.9 | 0.1 |

| 2-(trans-piperityl)-1-methylpyrrole **17** | | |
|---|---|---|
| calculated | measured | abs. difference |
| 134.7 | 136.4 | 1.7 |
| 132.3 | 134.1 | 1.8 |
| 123.7 | 123.4 | 0.3 |
| 120.7 | 121.4 | 0.7 |
| 110.2 | 106.6 | 3.6 |
| 106.6 | 106.4 | 0.2 |
| 43.4 | 44.4 | 1.0 |
| 36.6 | 37.2 | 0.6 |
| 35.0 | 34.1 | 0.9 |
| 27.1 | 29.4 | 2.3 |
| 25.5 | 27.6 | 2.1 |
| 23.8 | 23.6 | 0.2 |
| 22.8 | 21.8 | 1.0 |
| 21.0 | 21.5 | 0.5 |
| 19.9 | 17.8 | 2.1 |

| 1-presilphiperfolanylpentane-1,4-dione **18** | | |
|---|---|---|
| calculated | measured | abs. difference |
| 217.8 | 214.2 | 3.6 |
| 208.1 | 207.9 | 0.2 |
| 62.3 | 63.2 | 0.9 |
| 60.1 | 59.2 | 0.9 |
| 57.8 | 58.9 | 1.1 |
| 47.4 | 48.8 | 1.4 |
| 43.7 | 45.6 | 1.9 |
| 42.1 | 42.5 | 0.4 |
| 39.7 | 40.3 | 0.6 |
| 38.6 | 37.2 | 1.4 |
| 38.4 | 37.0 | 1.4 |
| 35.7 | 35.4 | 0.3 |
| 35.6 | 34.6 | 1.0 |
| 30.3 | 30.4 | 0.1 |
| 30.0 | 30.2 | 0.2 |
| 28.5 | 29.1 | 0.6 |
| 28.0 | 28.7 | 0.7 |
| 22.2 | 22.1 | 0.1 |
| 22.2 | 20.4 | 1.8 |
| 18.0 | 17.8 | 0.2 |

| 2-presilphiperfolanyl-1-methylpyrrole **19** | | |
|---|---|---|
| calculated | measured | abs. difference |
| 135.4 | 135.3 | 0.1 |
| 123.0 | 125.5 | 2.5 |
| 107.4 | 106.0 | 1.4 |
| 106.2 | 105.0 | 1.2 |
| 62.2 | 62.5 | 0.3 |
| 53.6 | 59.3 | 5.7 |
| 48.2 | 50.3 | 2.1 |
| 47.7 | 48.4 | 0.7 |
| 46.2 | 45.9 | 0.3 |
| 41.6 | 42.5 | 0.9 |
| 40.7 | 40.1 | 0.6 |
| 38.6 | 38.4 | 0.2 |
| 36.9 | 37.5 | 0.6 |
| 29.2 | 31.0 | 1.8 |
| 26.8 | 29.0 | 2.2 |
| 25.5 | 28.7 | 3.2 |
| 23.6 | 22.1 | 1.5 |
| 20.2 | 20.9 | 0.7 |
| 18.7 | 18.4 | 0.3 |
| 12.6 | 13.5 | 0.9 |

| (2Z)-1-presilphiperfolanylpent-2-ene-1,4-dione **20** | | |
|---|---|---|
| calculated | measured | abs. difference |
| 206.3 | 204.3 | 2.0 |
| 204.5 | 203.9 | 0.6 |
| 146.2 | 142.0 | 4.2 |
| 130.0 | 128.9 | 1.1 |
| 62.0 | 62.0 | 0.0 |
| 59.2 | 58.3 | 0.9 |
| 58.3 | 58.3 | 0.0 |
| 47.8 | 48.6 | 0.8 |
| 44.5 | 46.0 | 1.5 |
| 41.3 | 42.0 | 0.7 |
| 39.4 | 40.4 | 1.0 |
| 36.0 | 36.1 | 0.1 |
| 35.8 | 34.3 | 1.5 |
| 30.0 | 29.5 | 0.5 |
| 29.9 | 29.3 | 0.6 |
| 29.3 | 29.1 | 0.2 |
| 28.3 | 28.7 | 0.4 |
| 22.0 | 22.2 | 0.2 |
| 22.0 | 19.9 | 2.1 |
| 18.0 | 18.0 | 0.0 |

# S15 NMR spectra

## S15.1 2-presilphiperfolanyl-5-methylfuran 10

$^{1}$H NMR (600 MHz, $CDCl_3$, 298K)

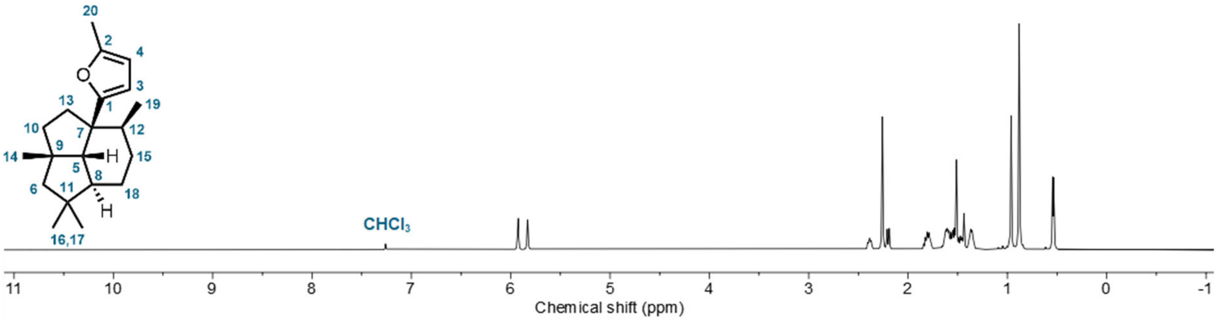


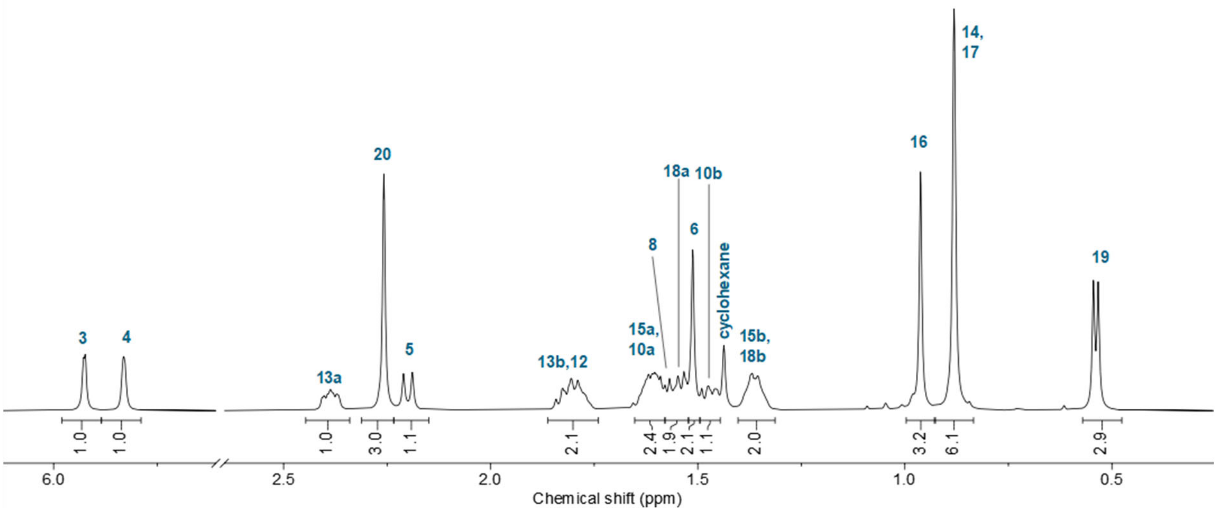


$^{13}$C NMR (151 MHz, $CDCl_3$, 298K)

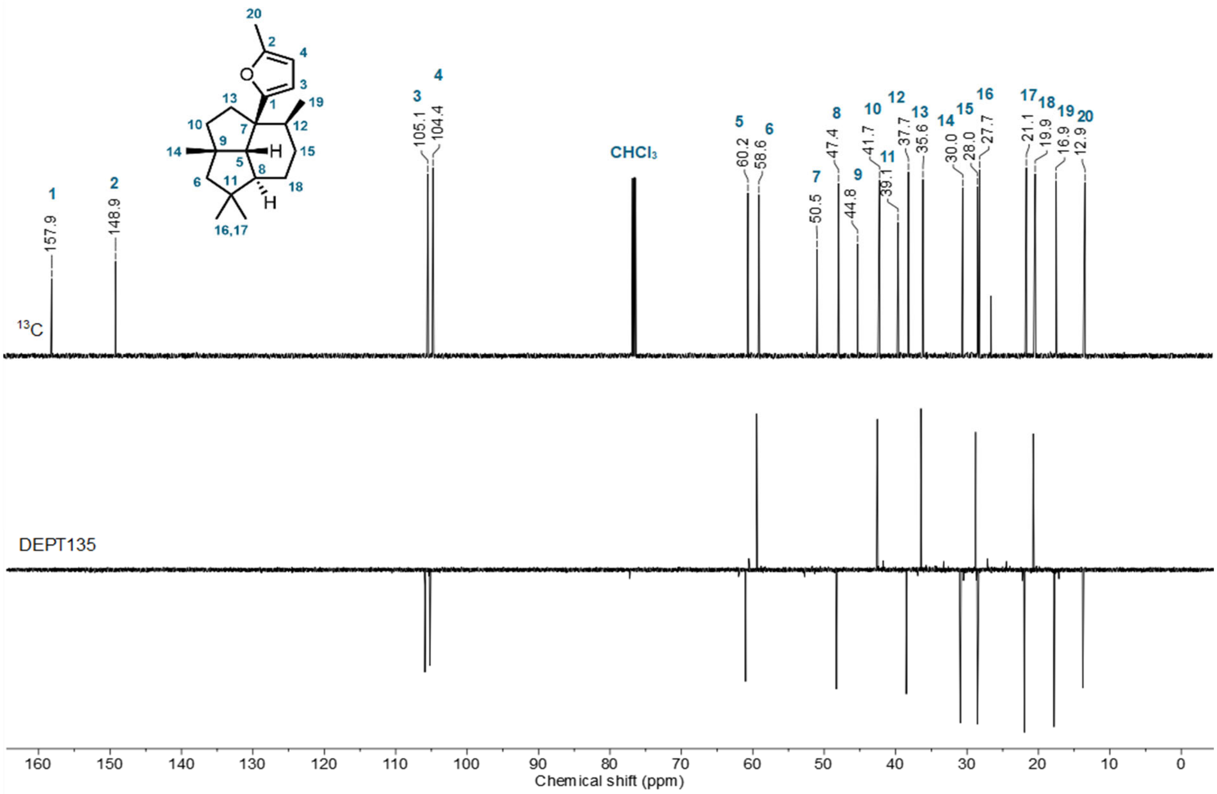

COSY (600 MHz, $CDCl_3$, 298K)

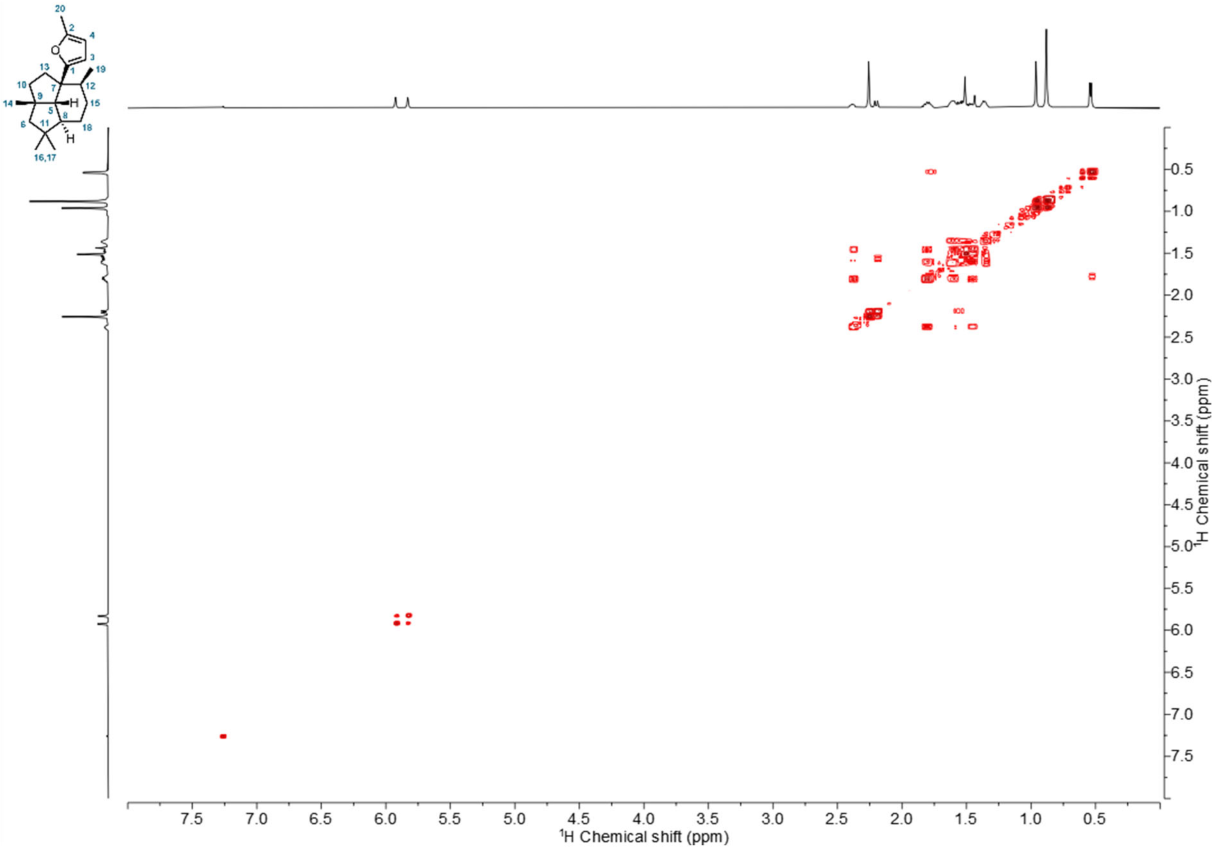


NOESY (600 MHz, $CDCl_3$, 298K)

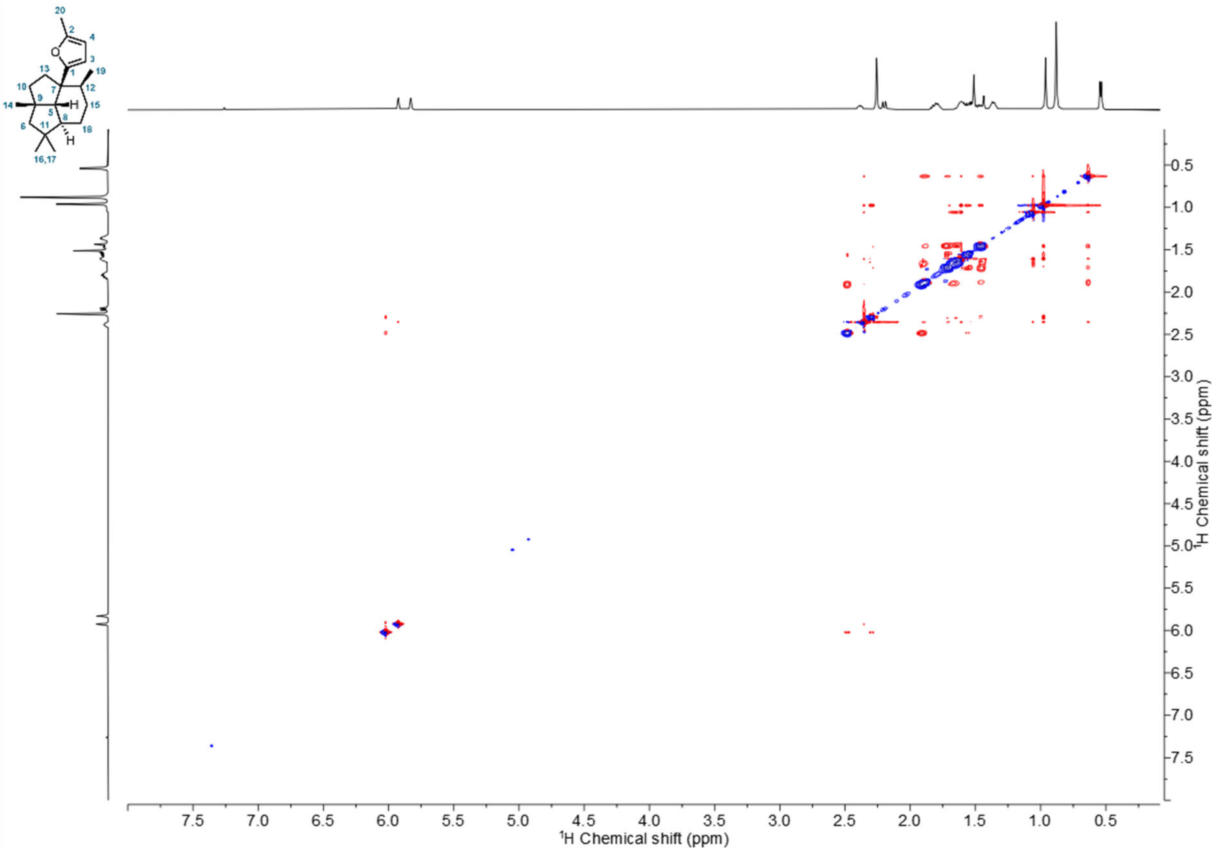

HSQC (600 MHz, $CDCl_3$, 298K)

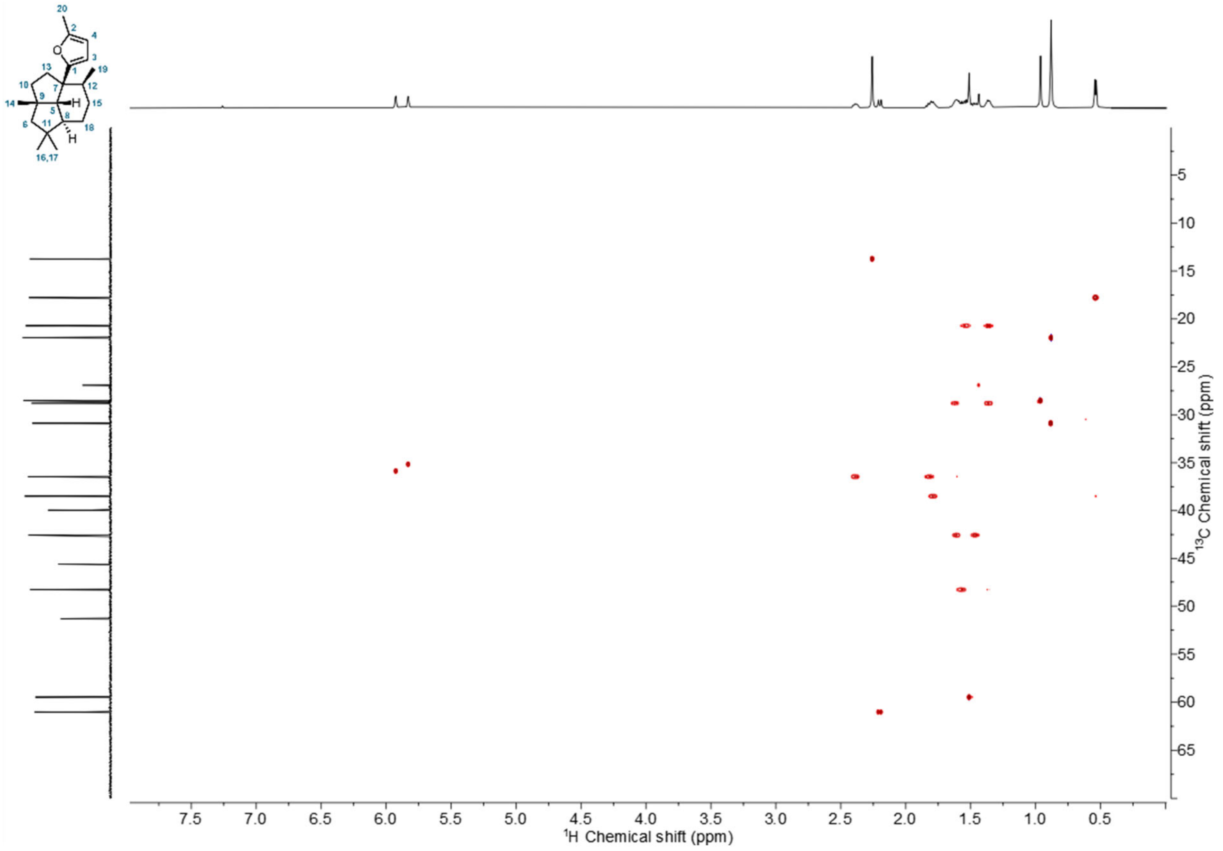


HMBC (600 MHz, $CDCl_3$, 298K)

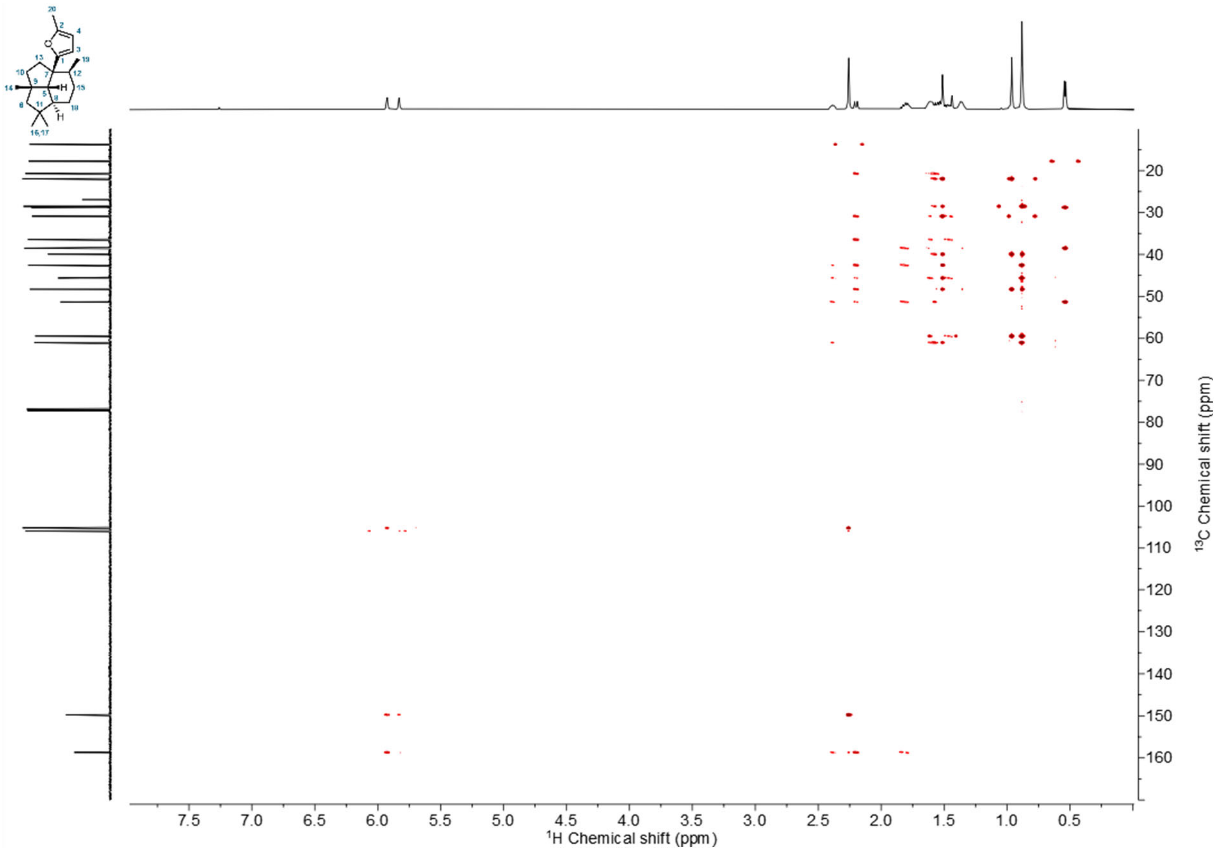

1,1 ADEQUATE (600 MHz, $CDCl_3$, 298K)

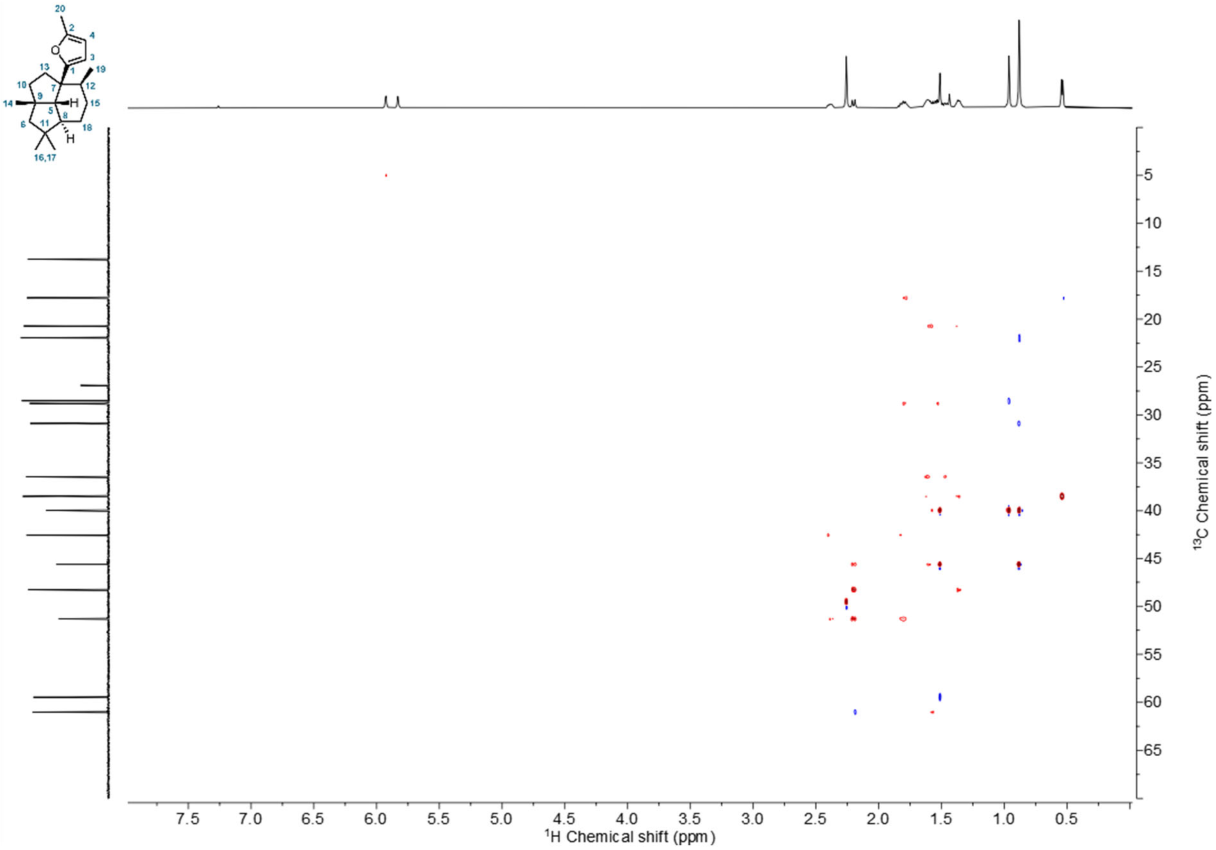

## S15.2 2-presilphiperfolanyl-5-methylfuran epimer 10b

As a 1:1 mixture with the epimer at C12.

$^1H$ NMR (600 MHz, $CDCl_3$, 298K)

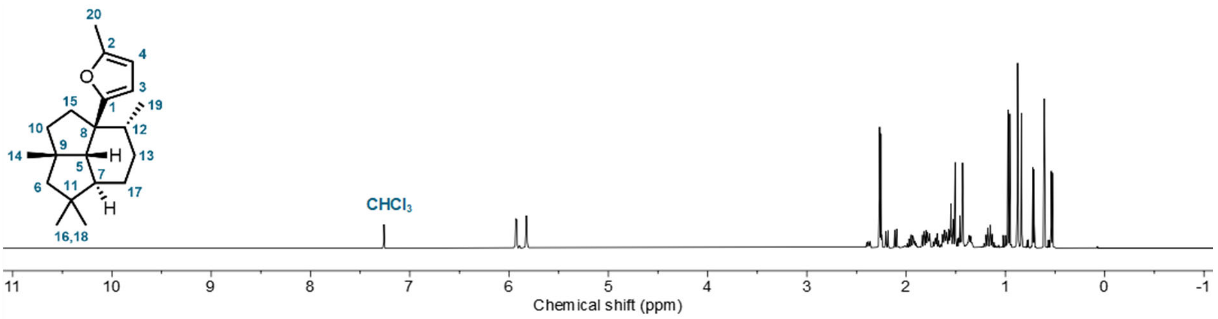

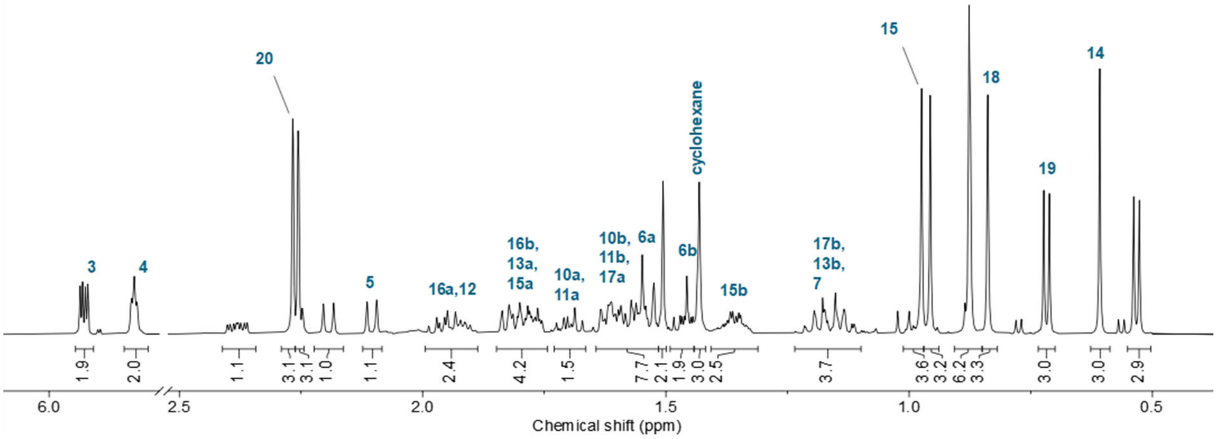

$^{13}C$ NMR (151 MHz, $CDCl_3$, 298K)

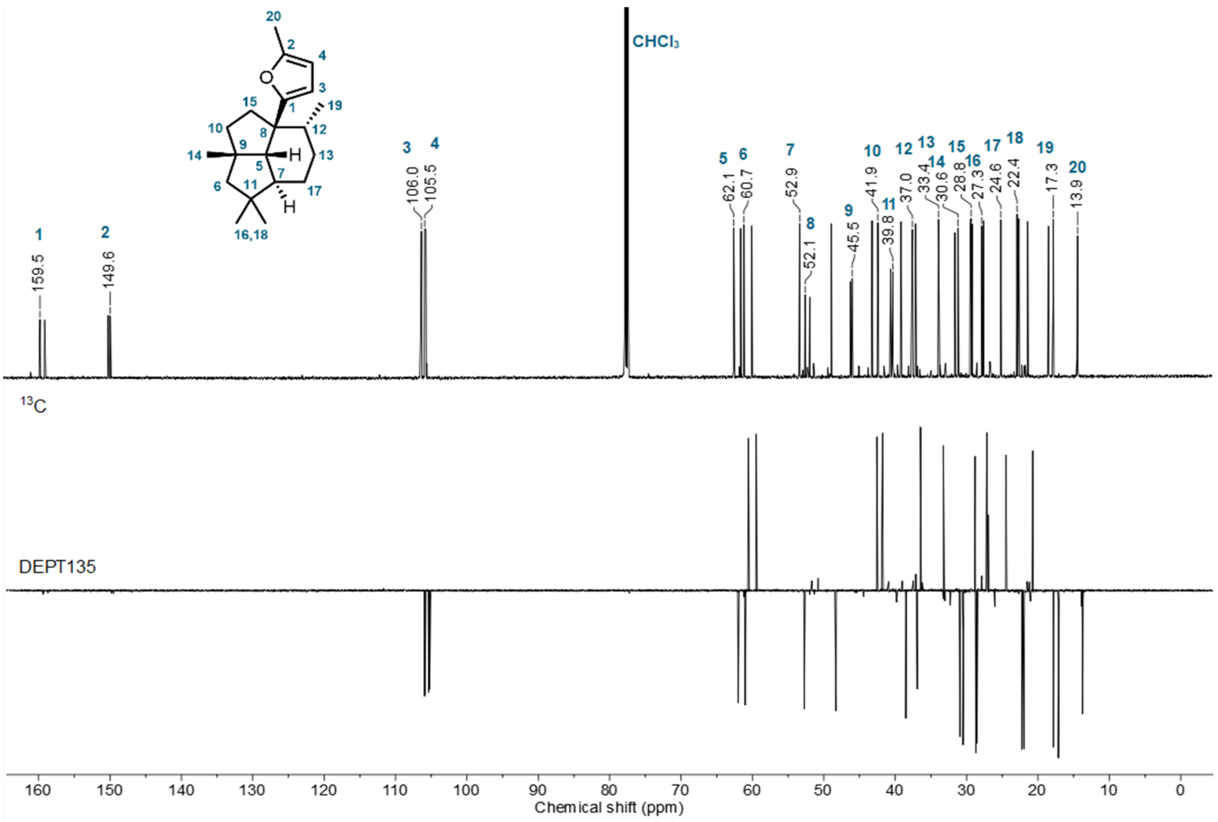

COSY (600 MHz, $CDCl_3$, 298K)

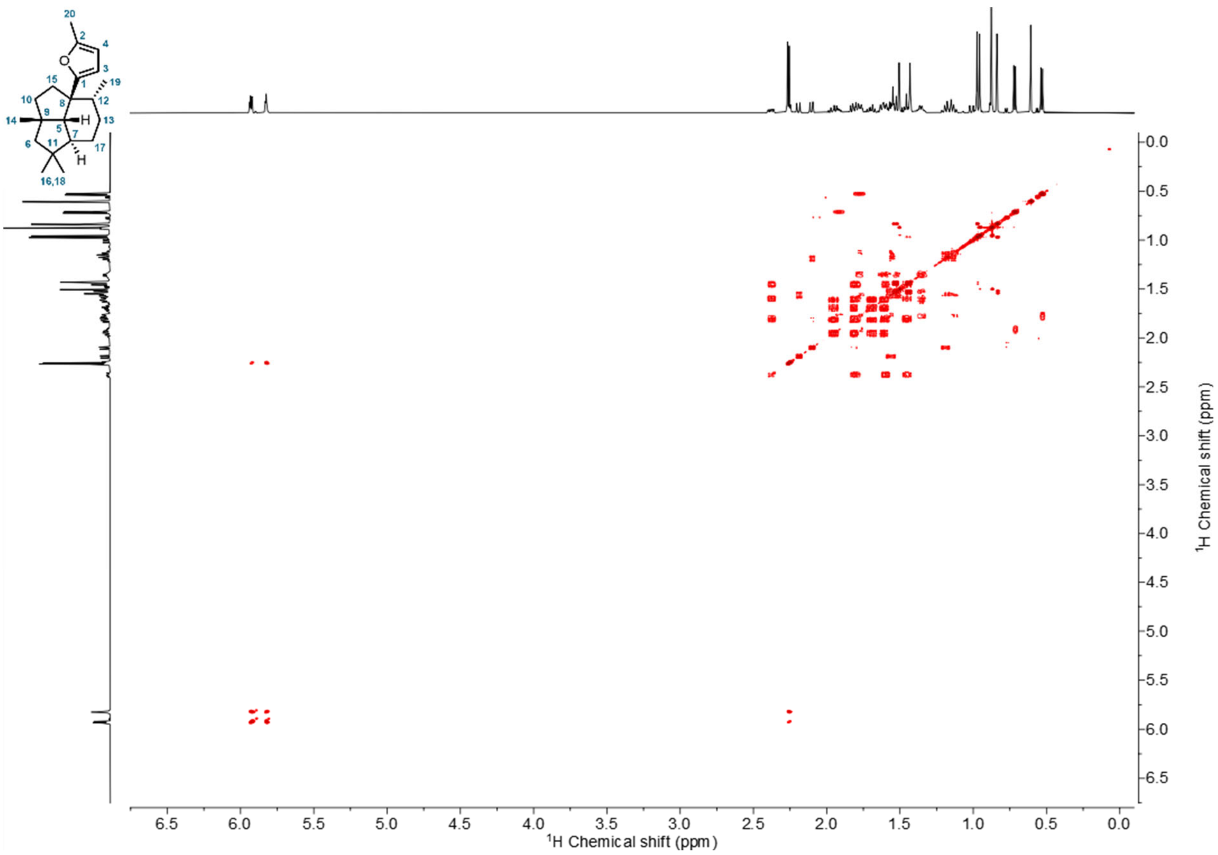


NOESY (600 MHz, $CDCl_3$, 298K)

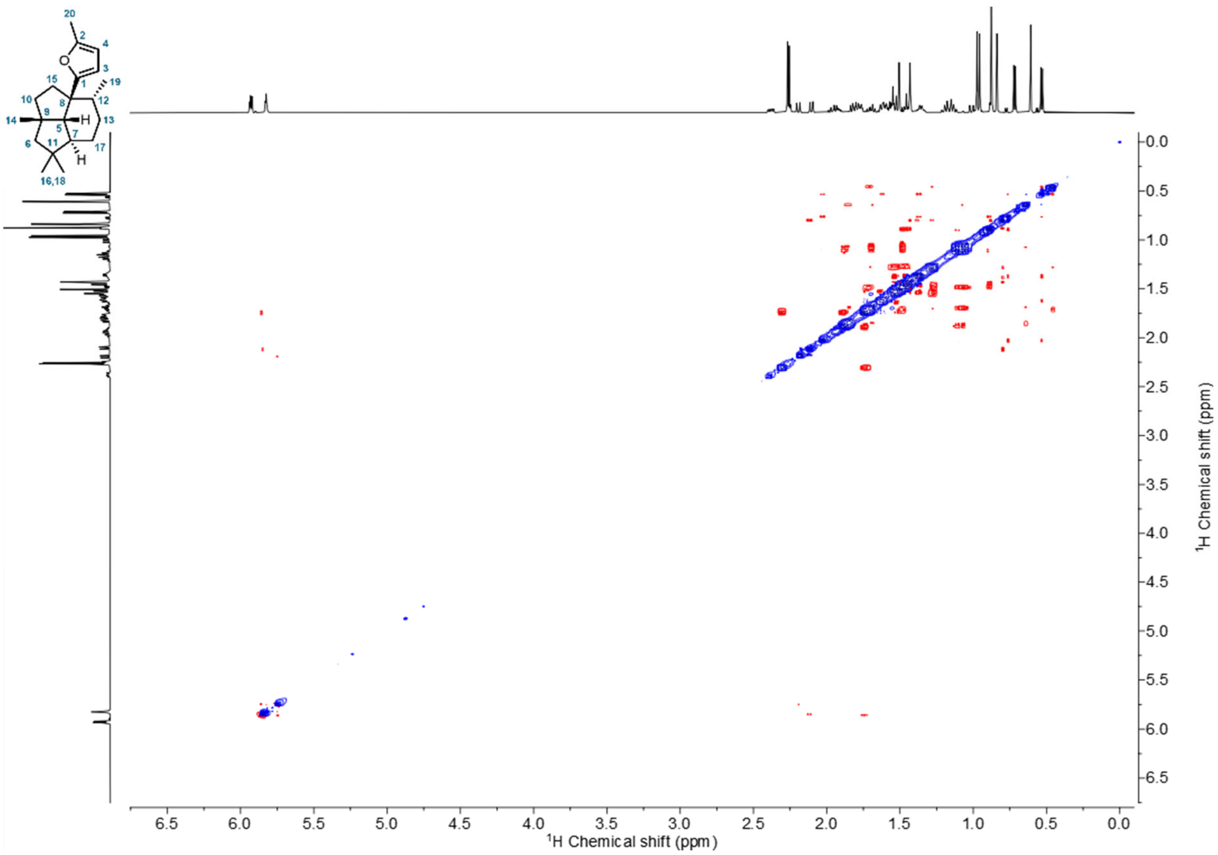

HSQC (600 MHz, $CDCl_3$, 298K)

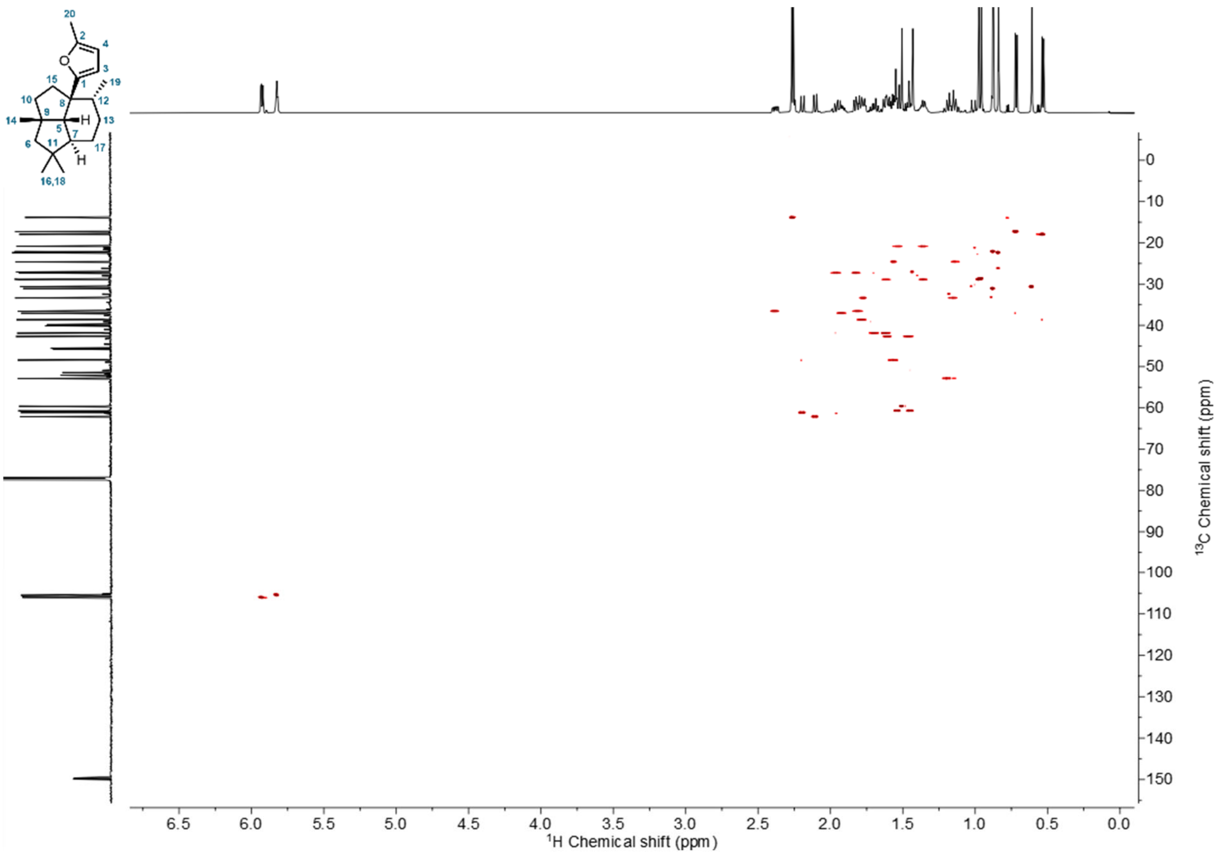


HMBC (600 MHz, $CDCl_3$, 298K)

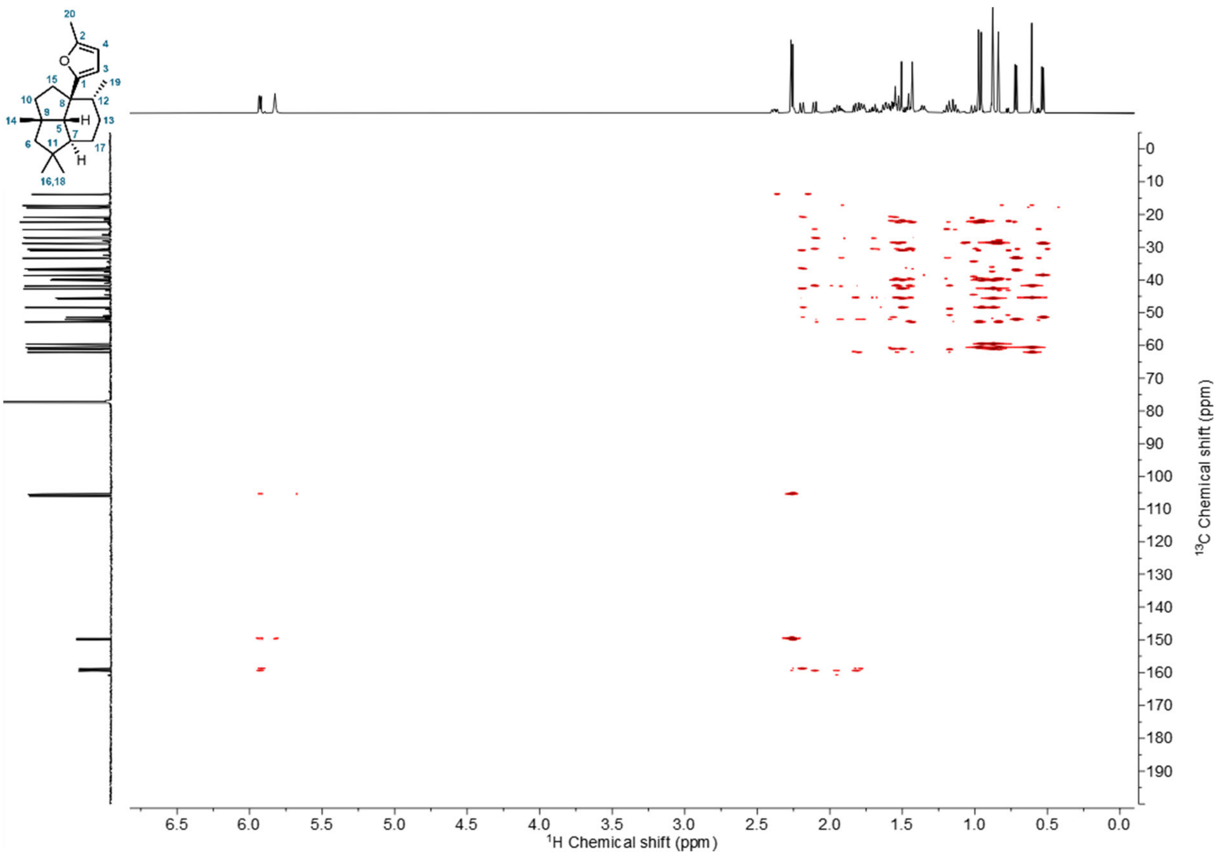

## S15.3 2-presilphiperfolanyl-4,5-dimethylfuran 11

$^{1}H$ NMR (600 MHz, $CDCl_3$, 298K)

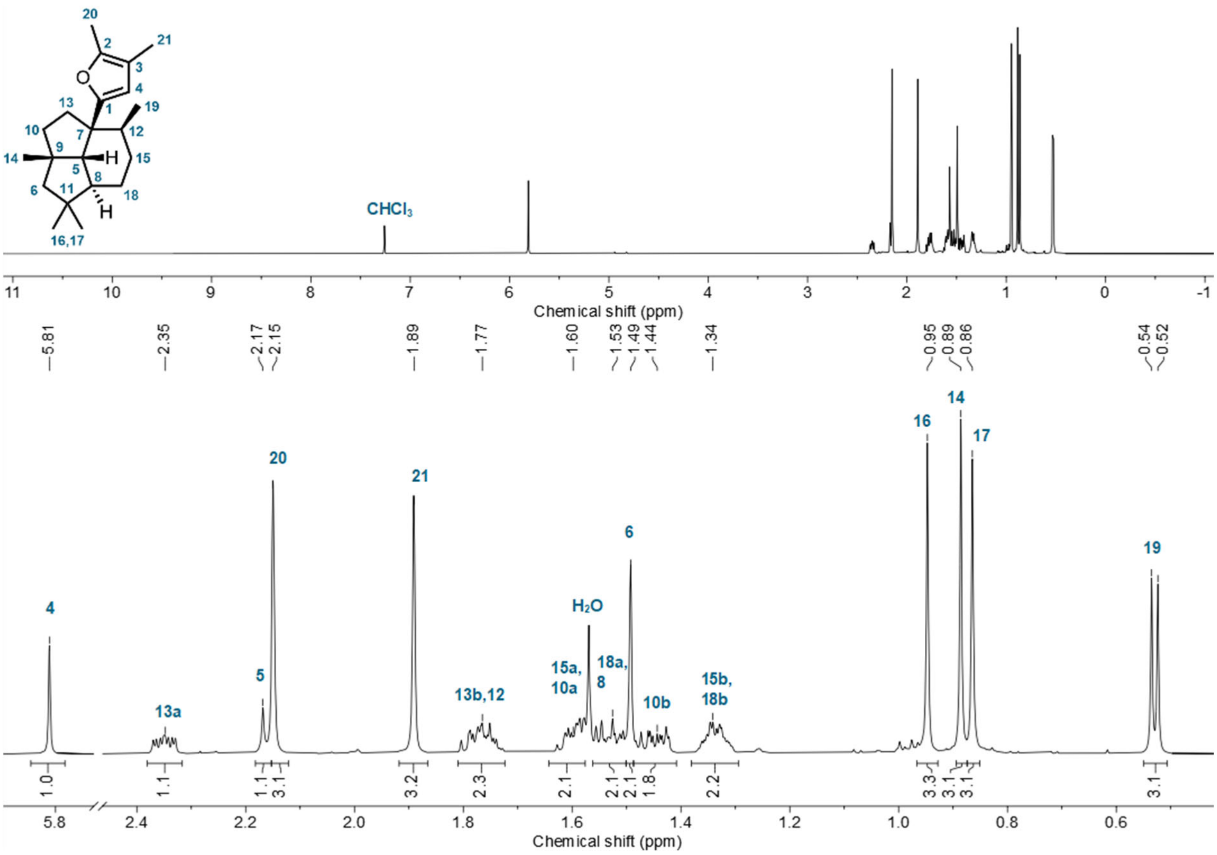


$^{13}C$ NMR (151 MHz, $CDCl_3$, 298K)

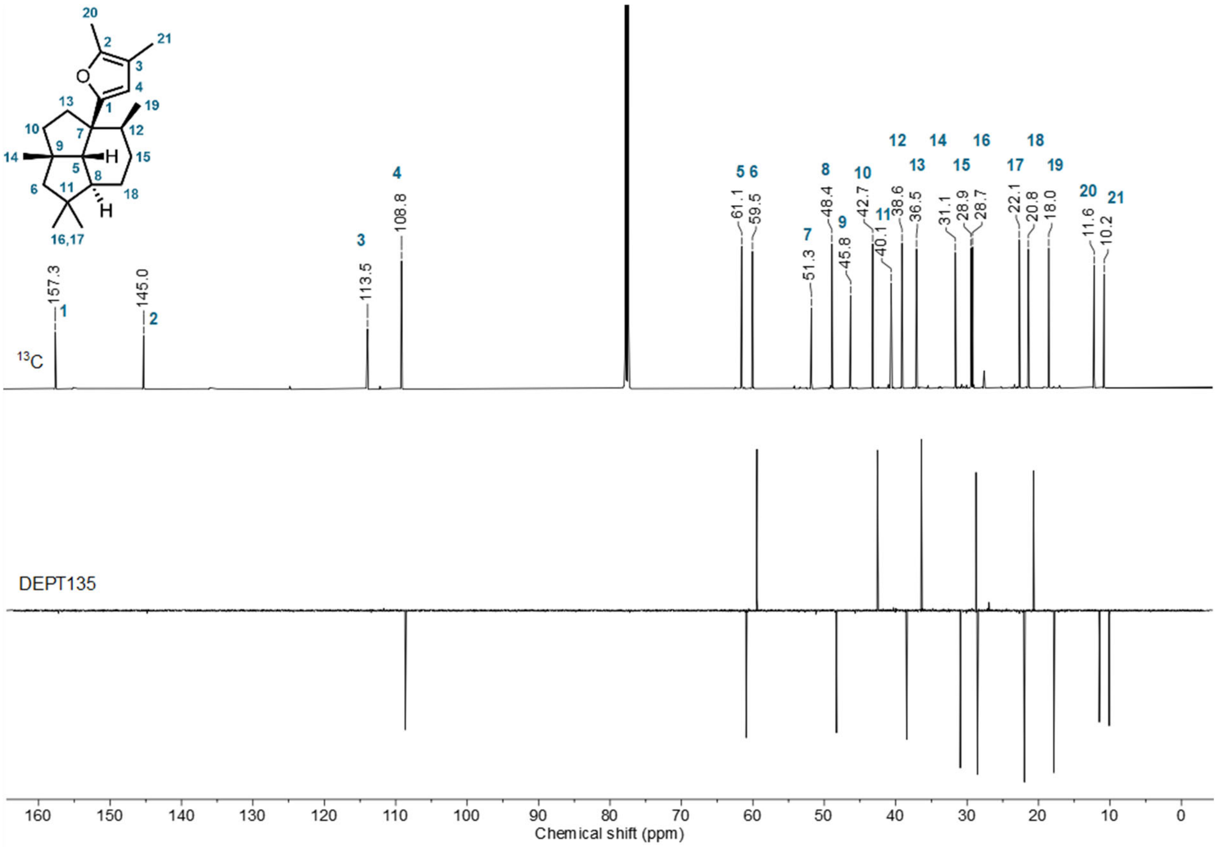

COSY (600 MHz, $CDCl_3$, 298K)

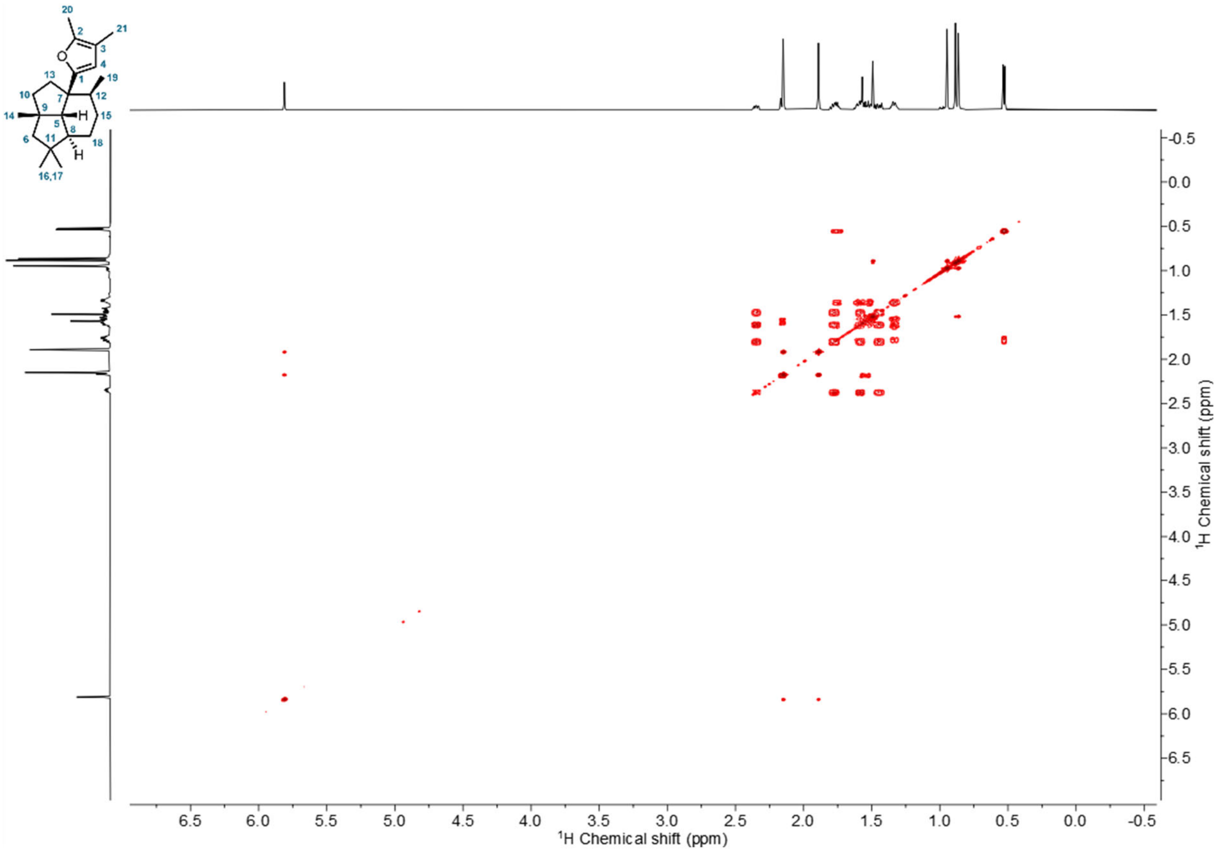


NOESY (600 MHz, $CDCl_3$, 298K)

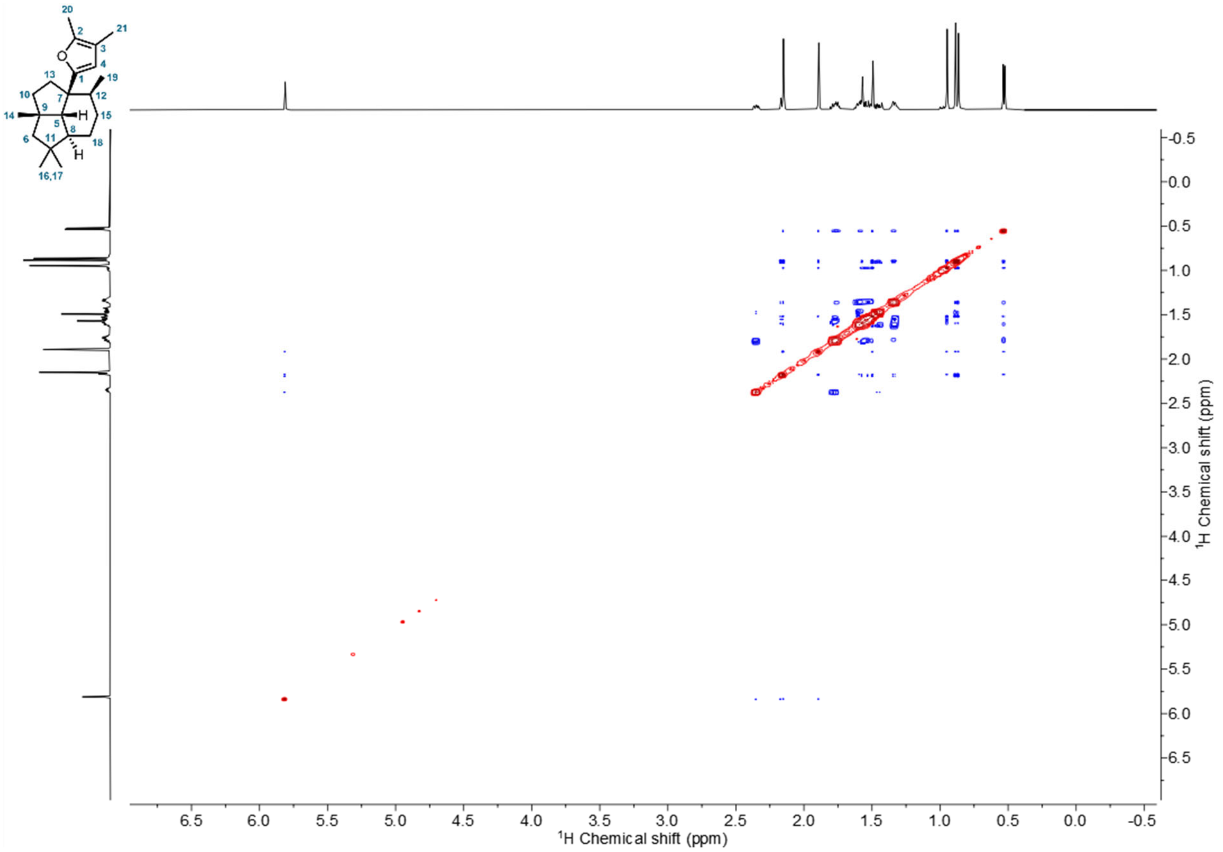

HSQC (600 MHz, $CDCl_3$, 298K)

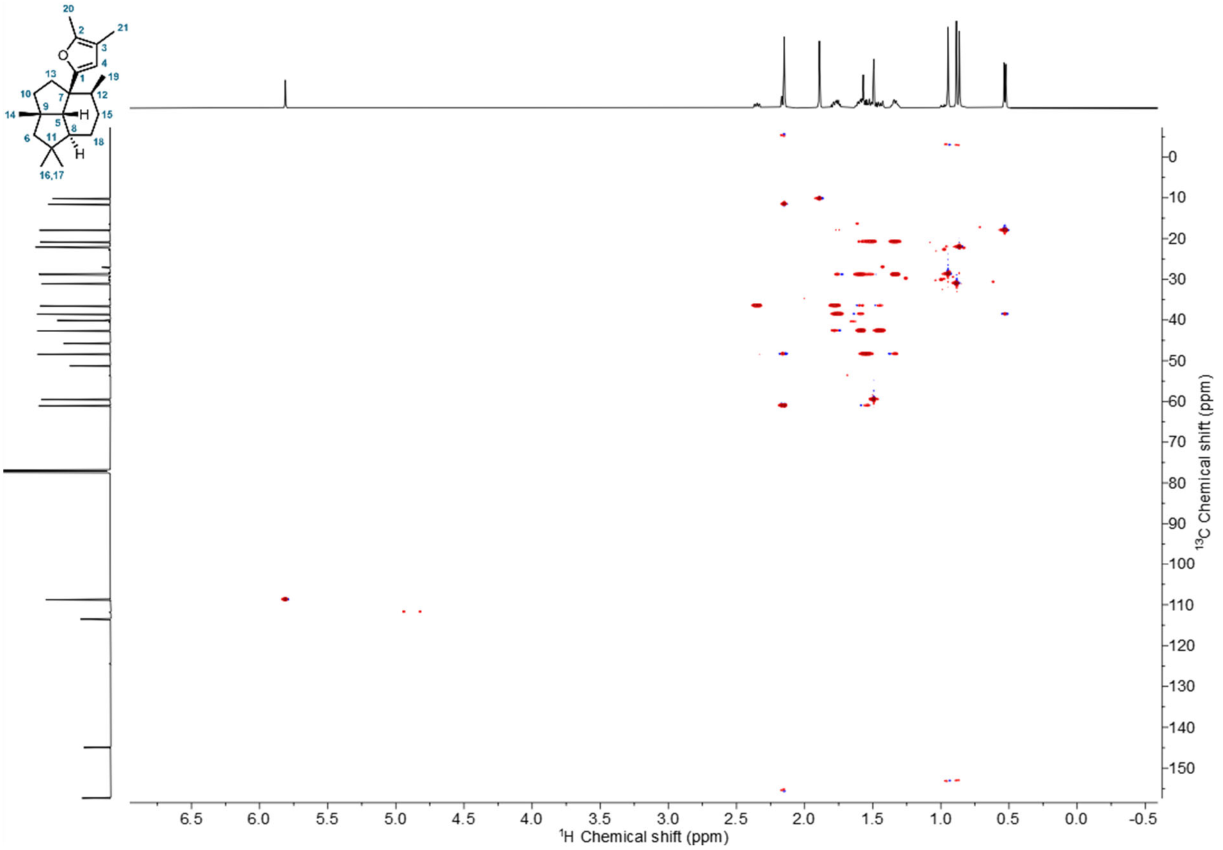


HMBC (600 MHz, $CDCl_3$, 298K)

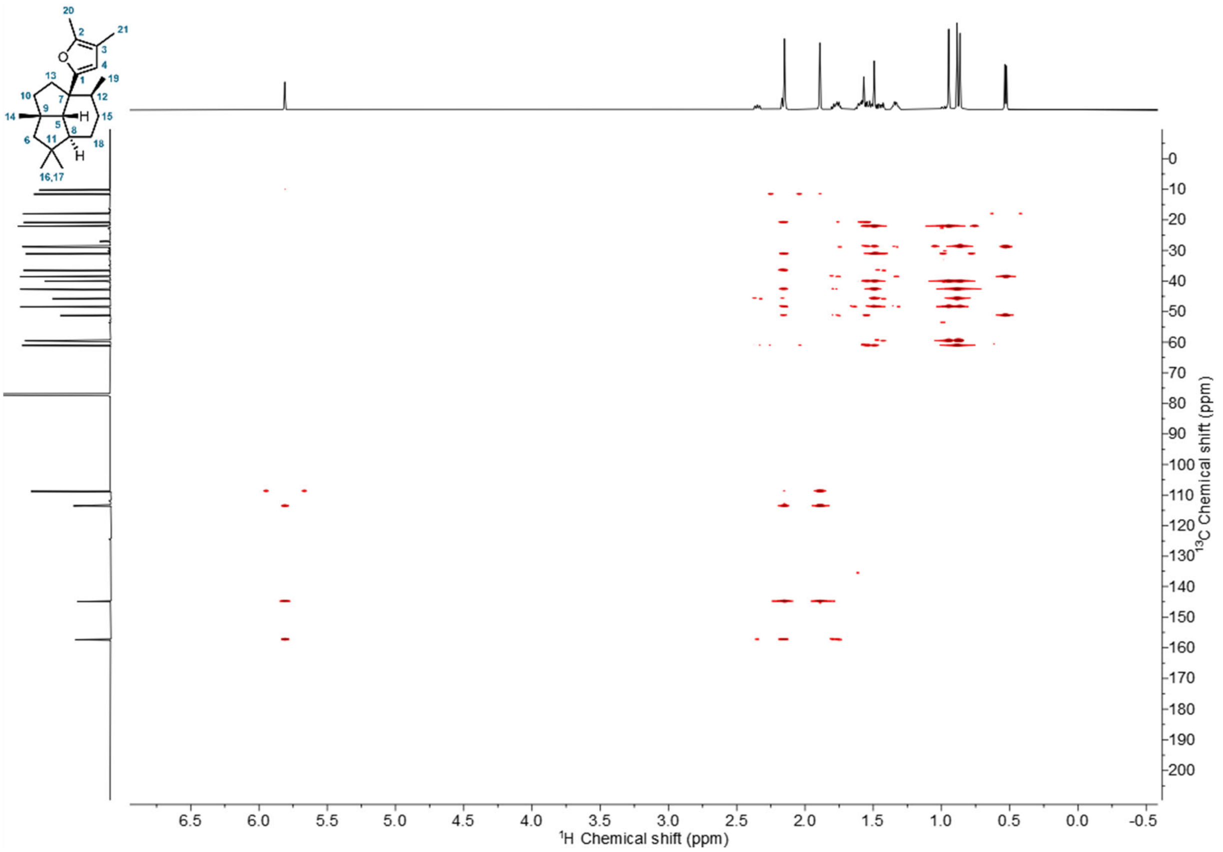

## S15.4 2-(*trans*-*p*-menth-1-en-3-yl)-5-methylfuran 12

$^1$H NMR (600 MHz, $CDCl_3$, 298K)

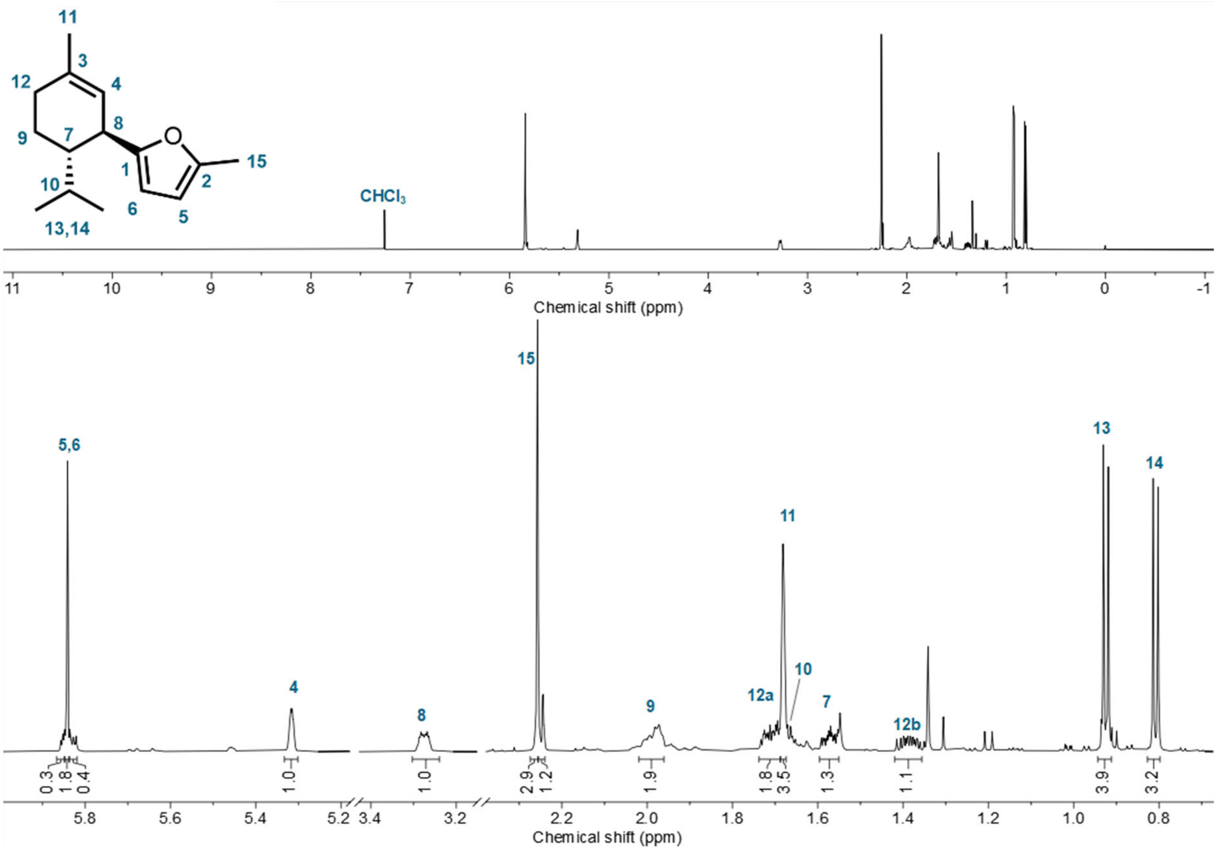


$^{13}$C NMR (151 MHz, $CDCl_3$, 298K)

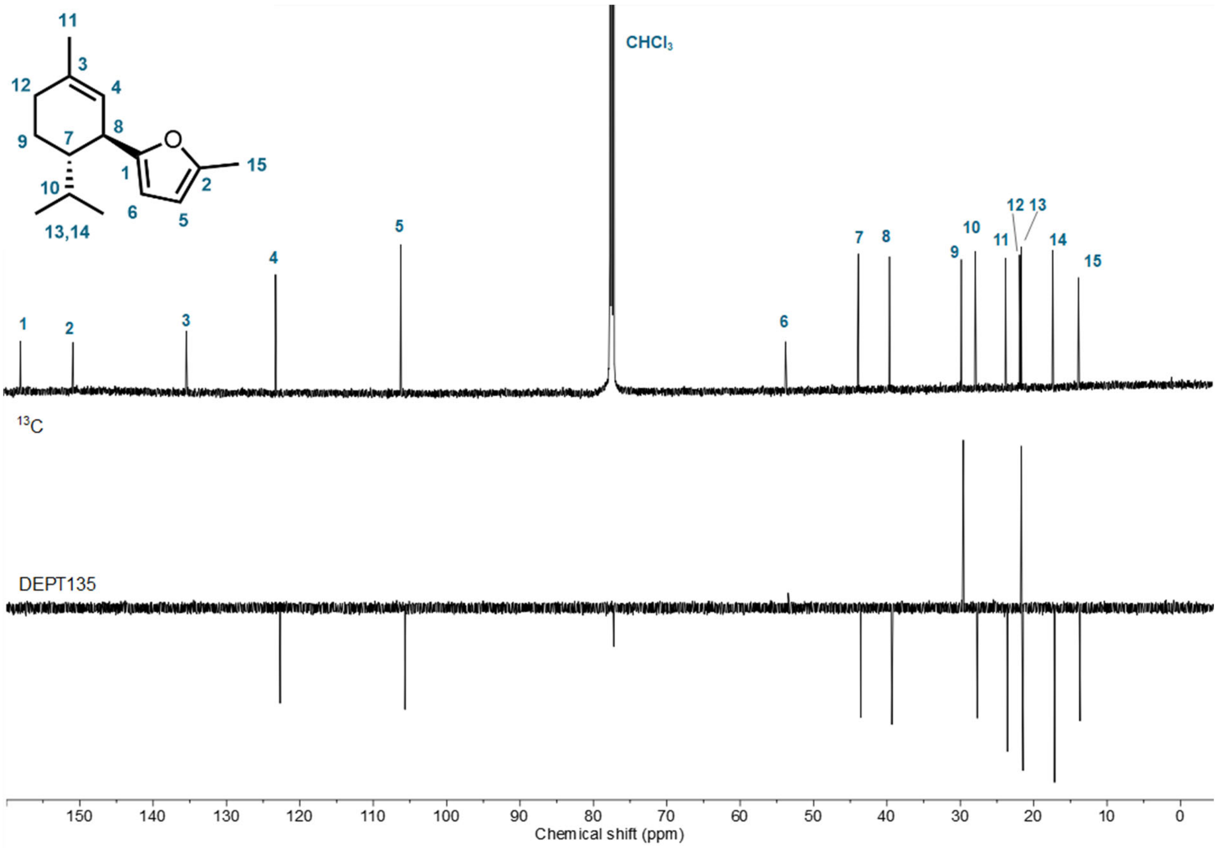

COSY (600 MHz, $CDCl_3$, 298K)

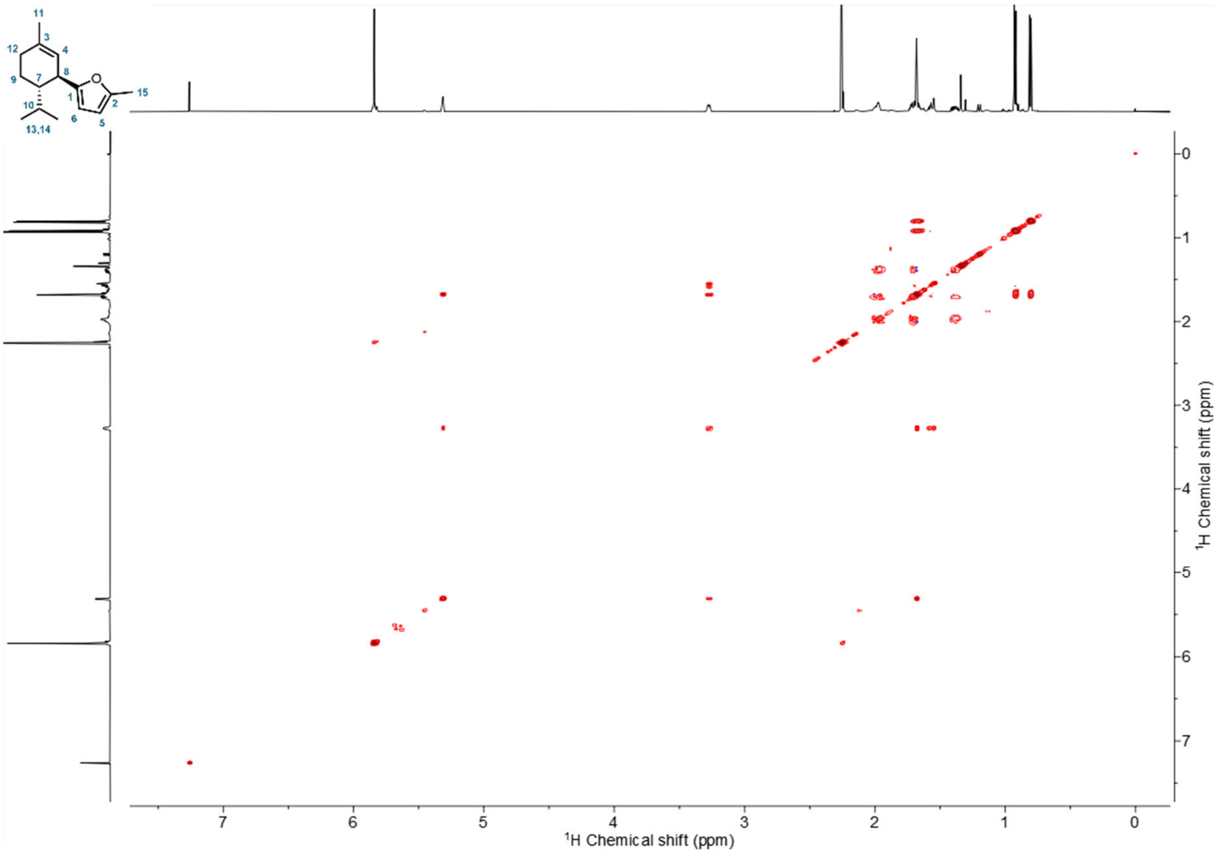


NOESY (600 MHz, $CDCl_3$, 298K)

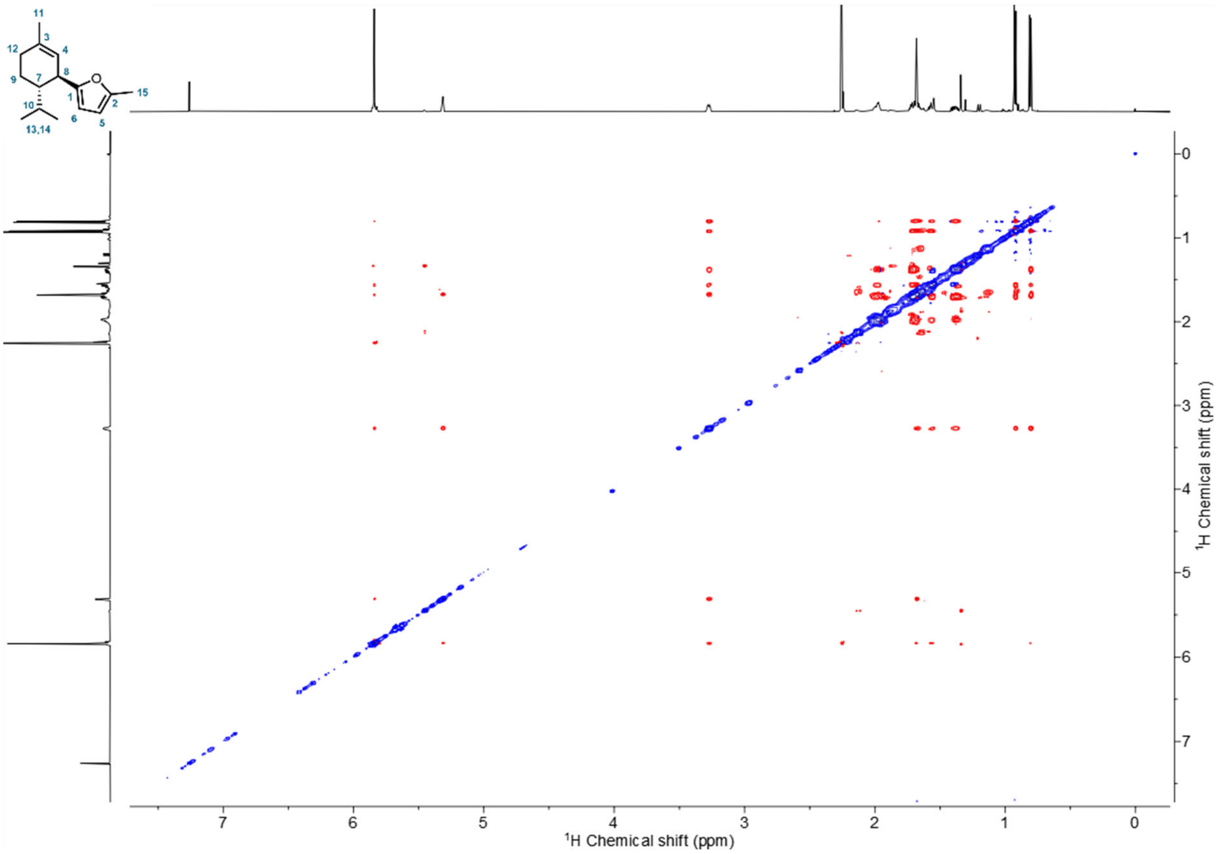

HSQC (600 MHz, $CDCl_3$, 298K)

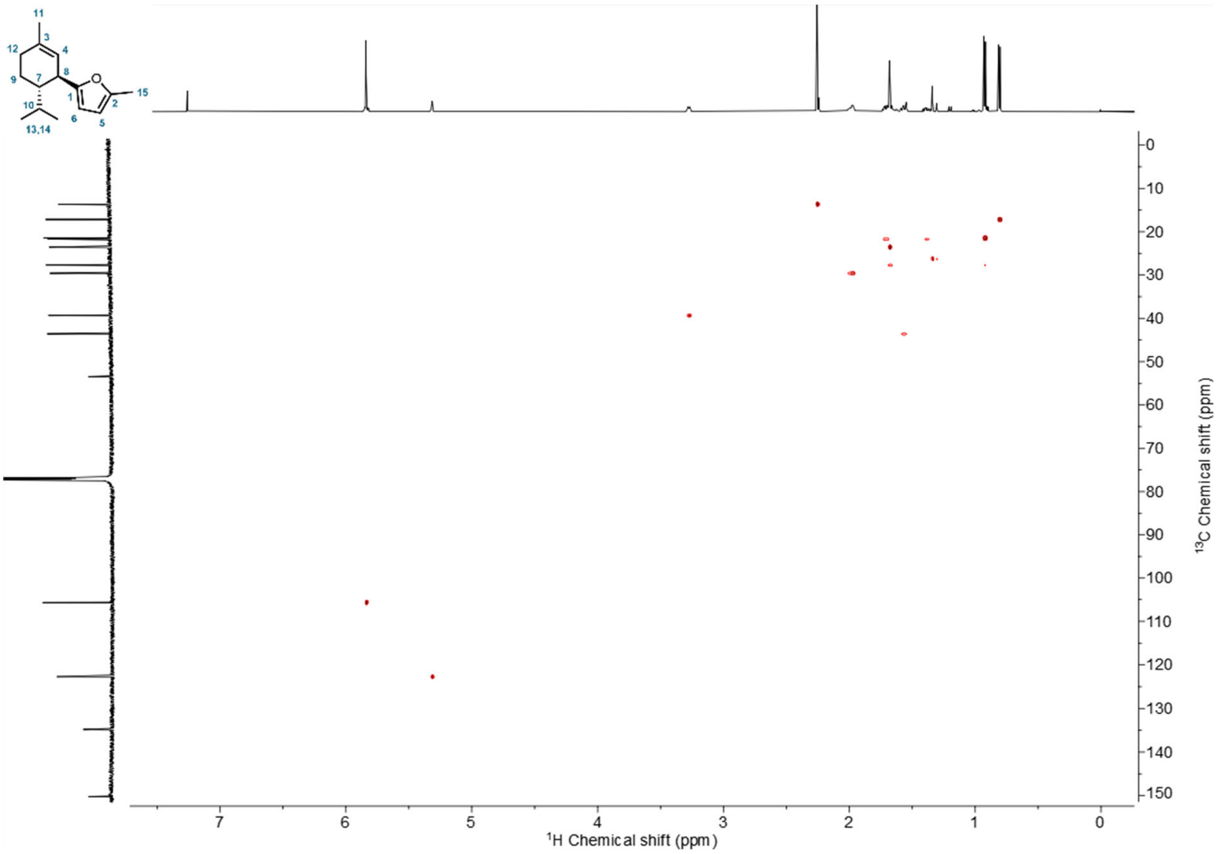


HMBC (600 MHz, $CDCl_3$, 298K)

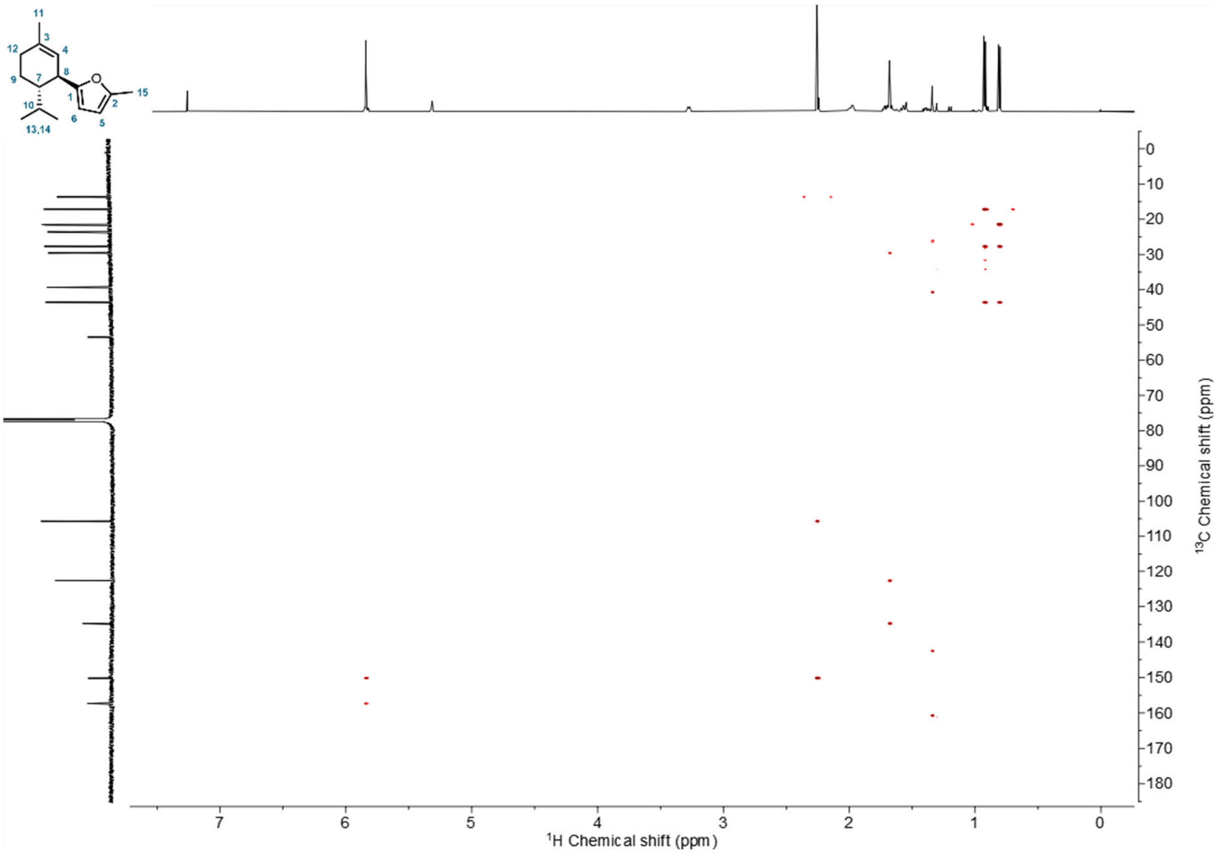

## S15.5 2-(*trans*-*p*-menth-1-en-3-yl)-4-methylfuran 13

$^{1}H$ NMR (600 MHz, $CDCl_3$, 298K)

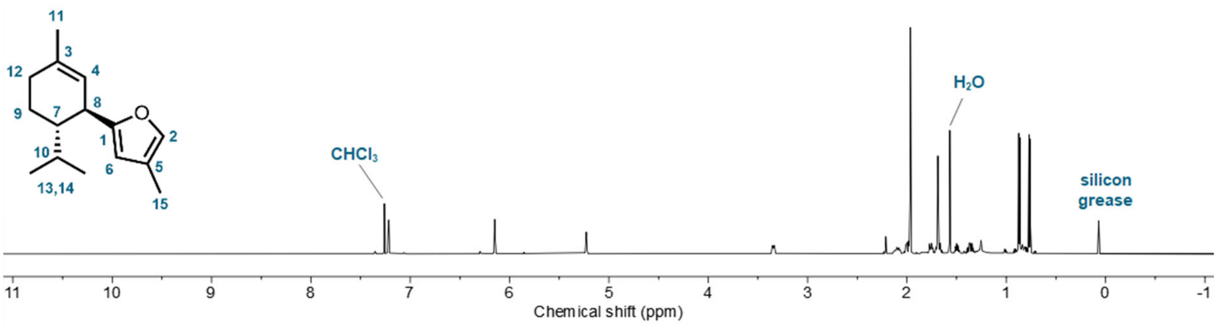


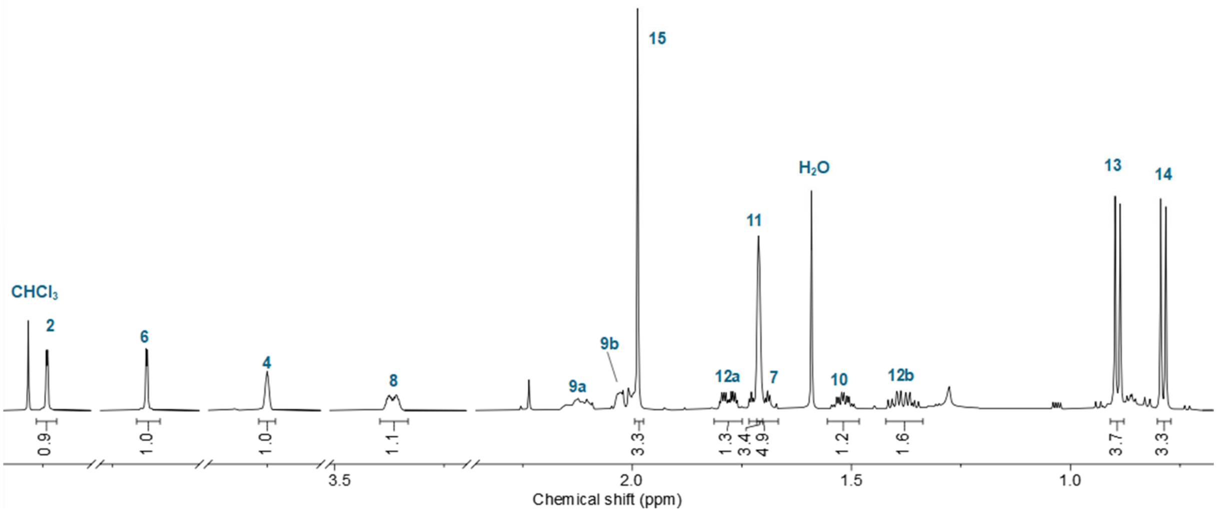


$^{13}C$ NMR (151 MHz, $CDCl_3$, 298K)

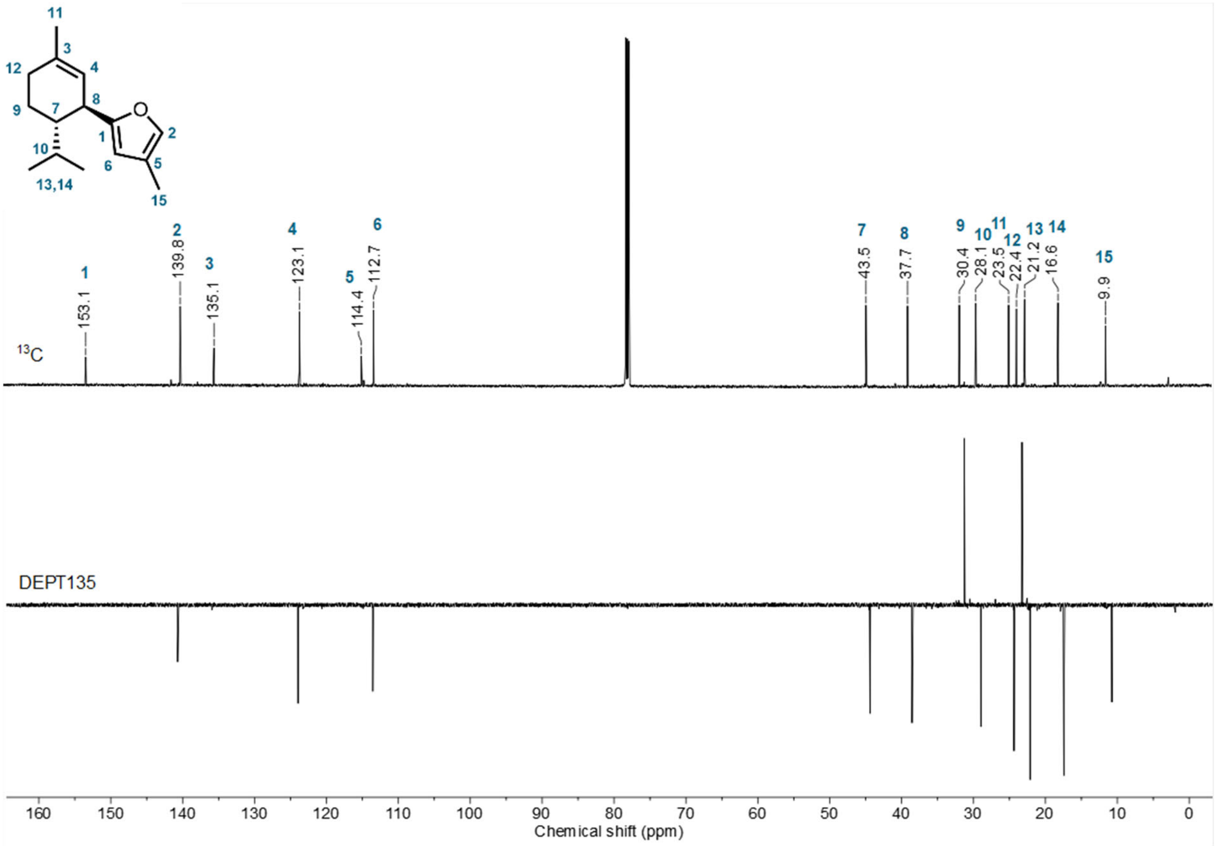

COSY (600 MHz, $CDCl_3$, 298K)

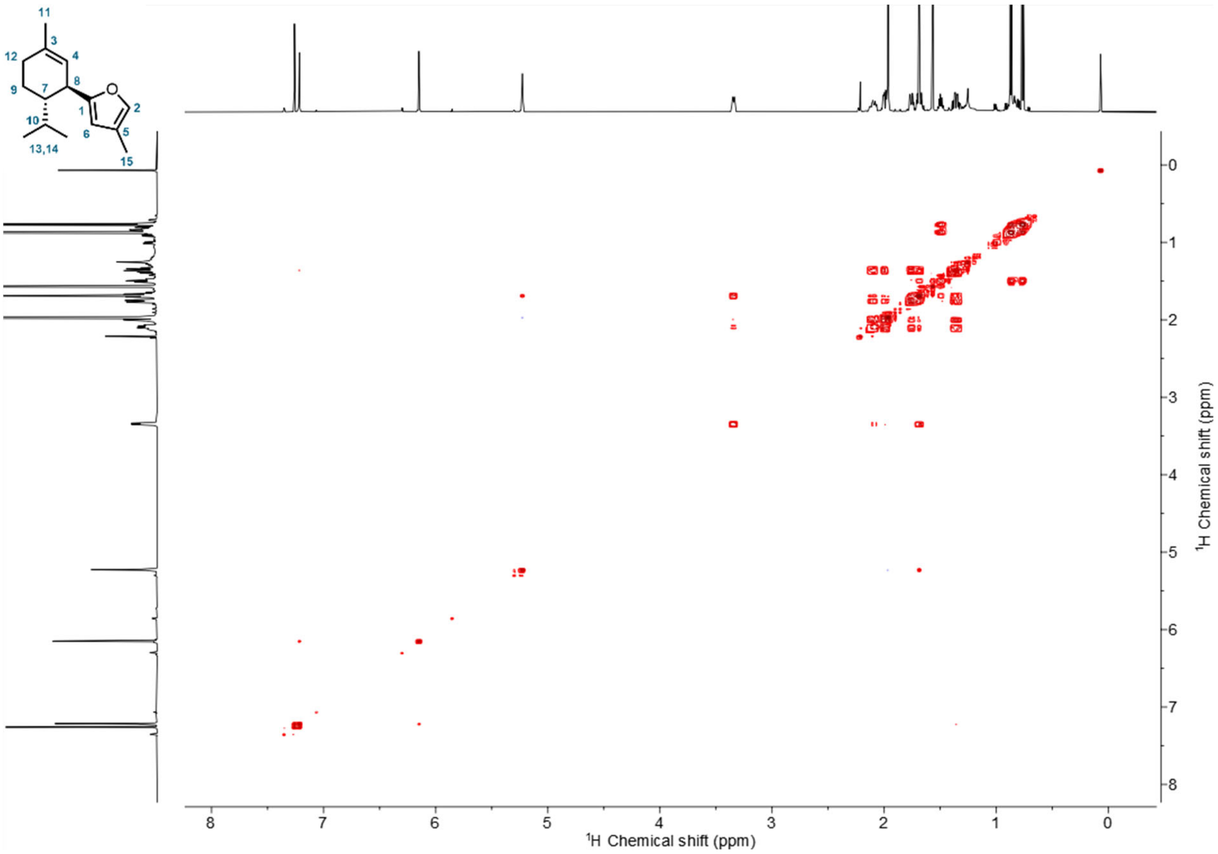


NOESY (600 MHz, $CDCl_3$, 298K)

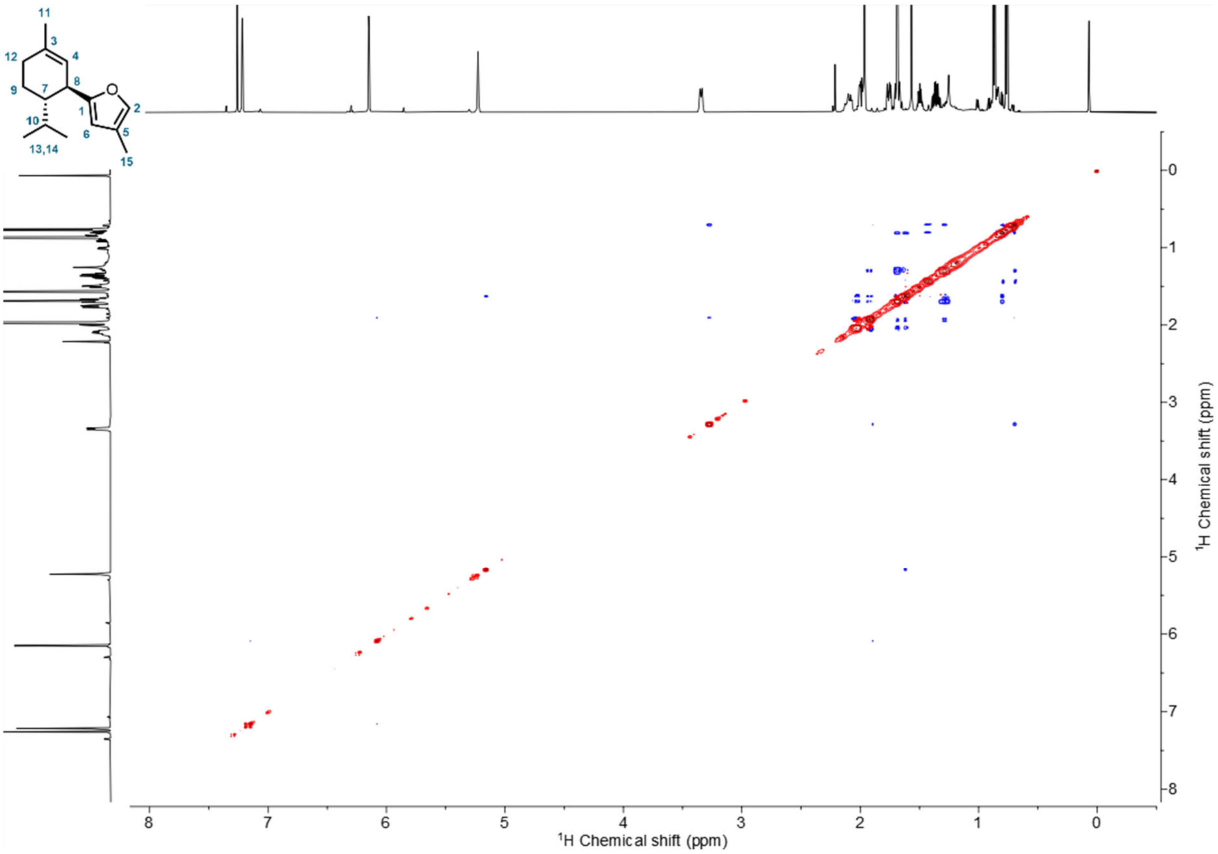

HSQC (600 MHz, $CDCl_3$, 298K)

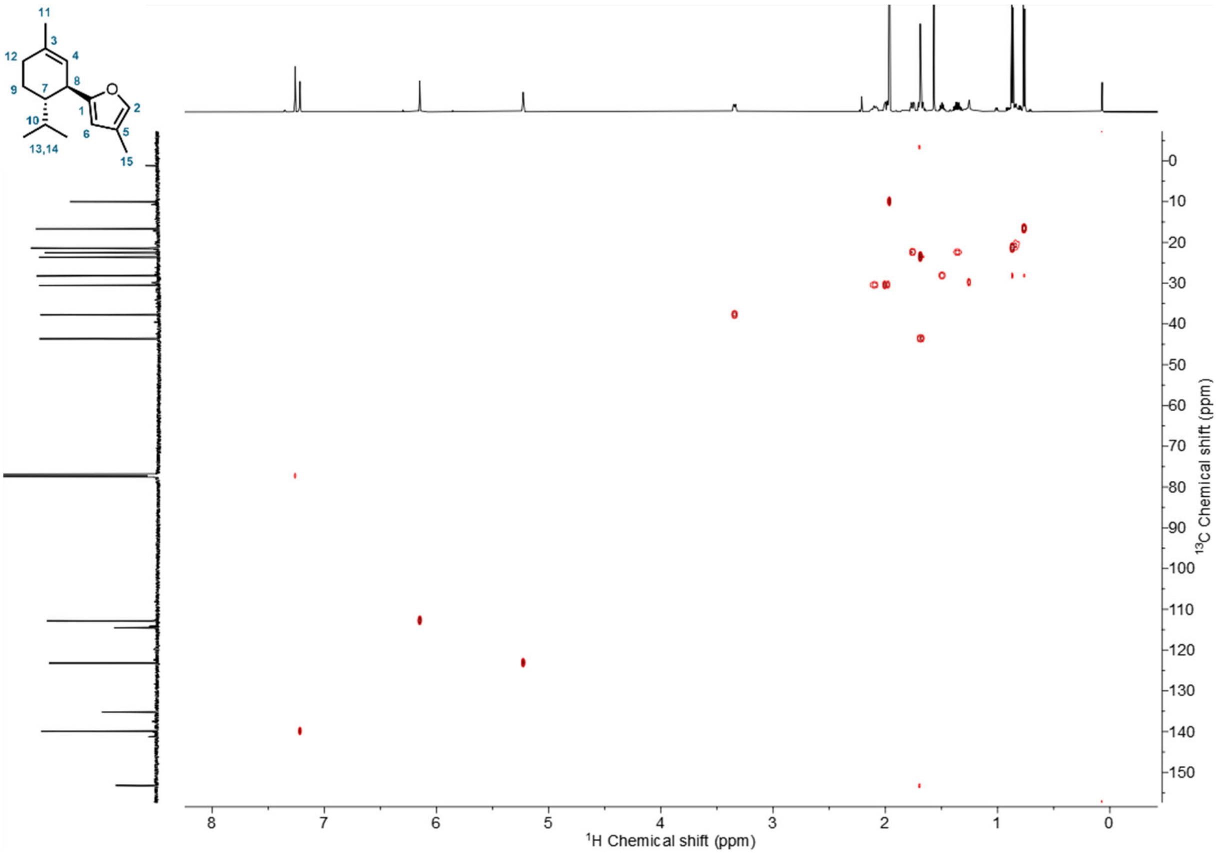


HMBC (600 MHz, $CDCl_3$, 298K)

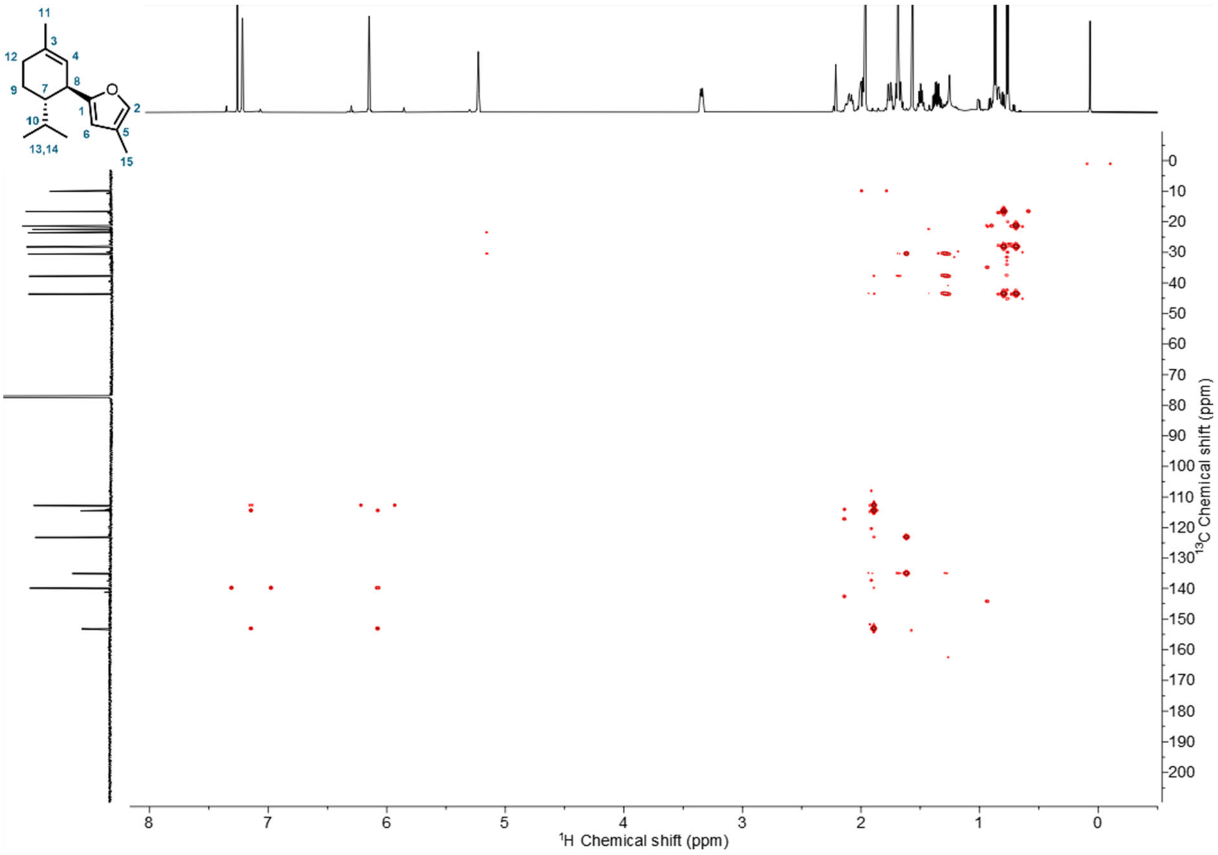

## S15.6 2-(*trans*-*p*-menth-1-en-3-yl)-4,5-dimethylfuran 14

$^1$H NMR (600 MHz, $CDCl_3$, 298K)

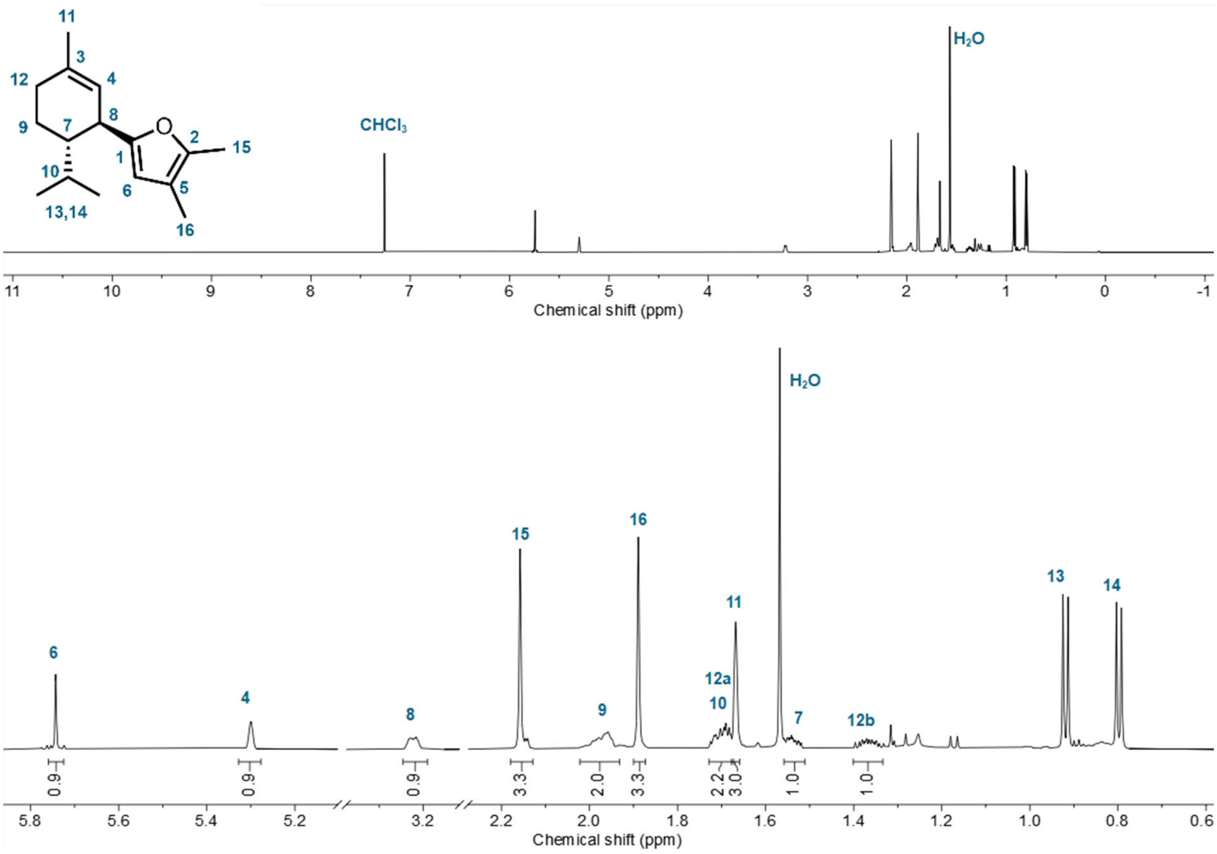


$^{13}$C NMR (151 MHz, $CDCl_3$, 298K)

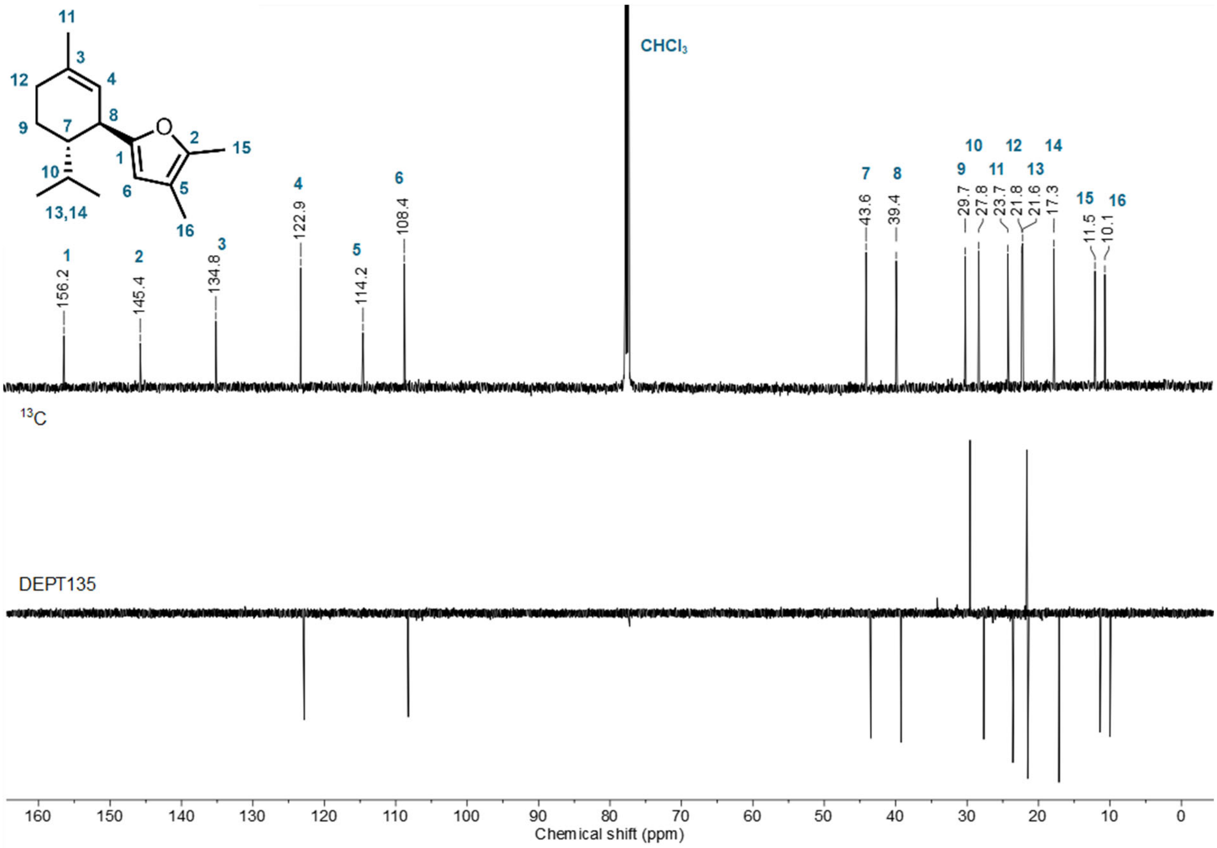

COSY (600 MHz, $CDCl_3$, 298K)

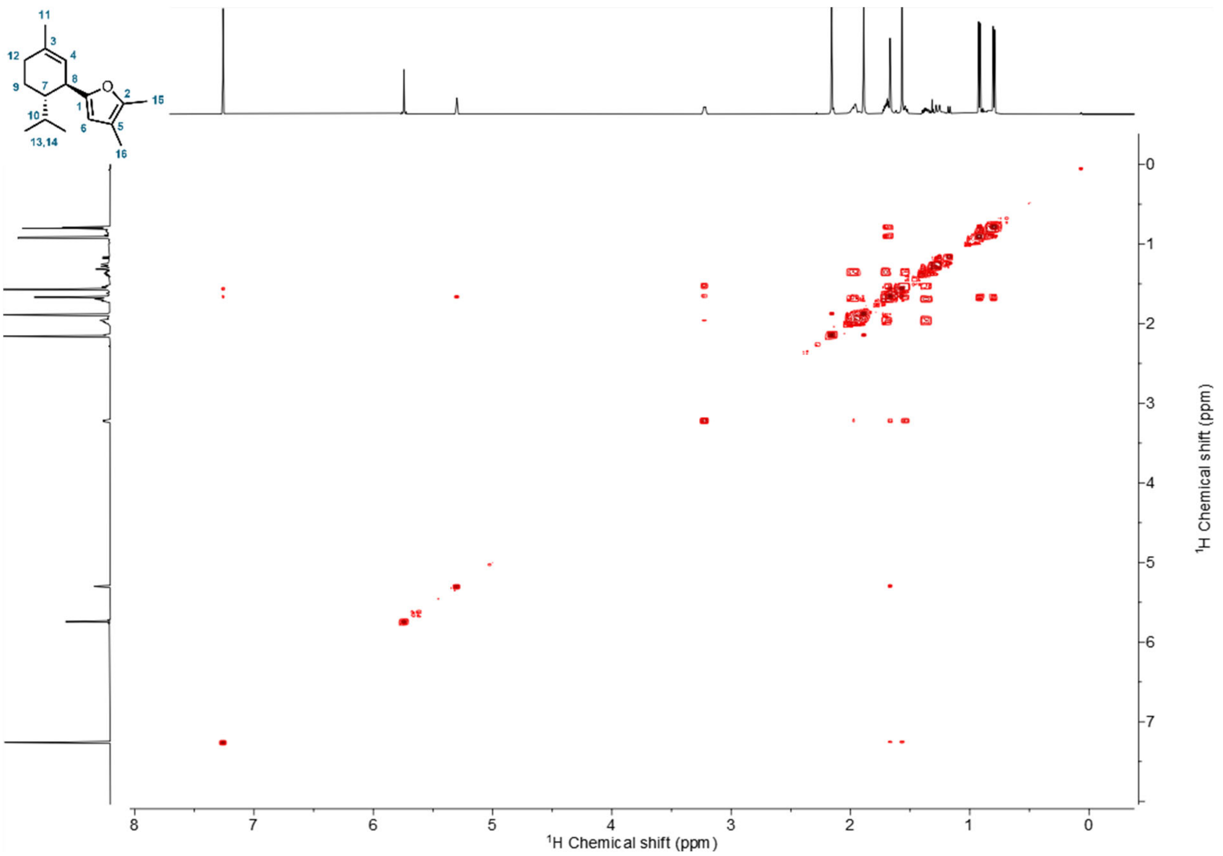


NOESY (600 MHz, $CDCl_3$, 298K)

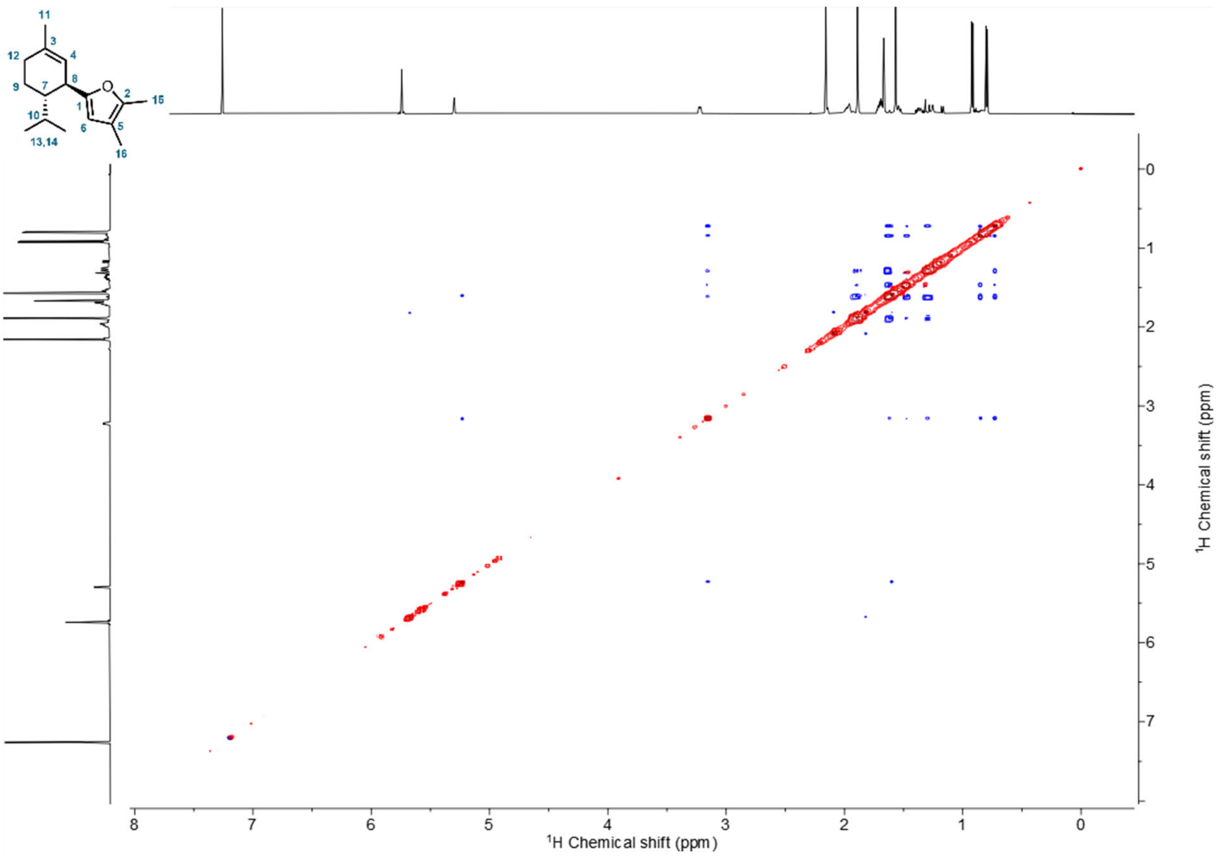

HSQC (600 MHz, $CDCl_3$, 298K)

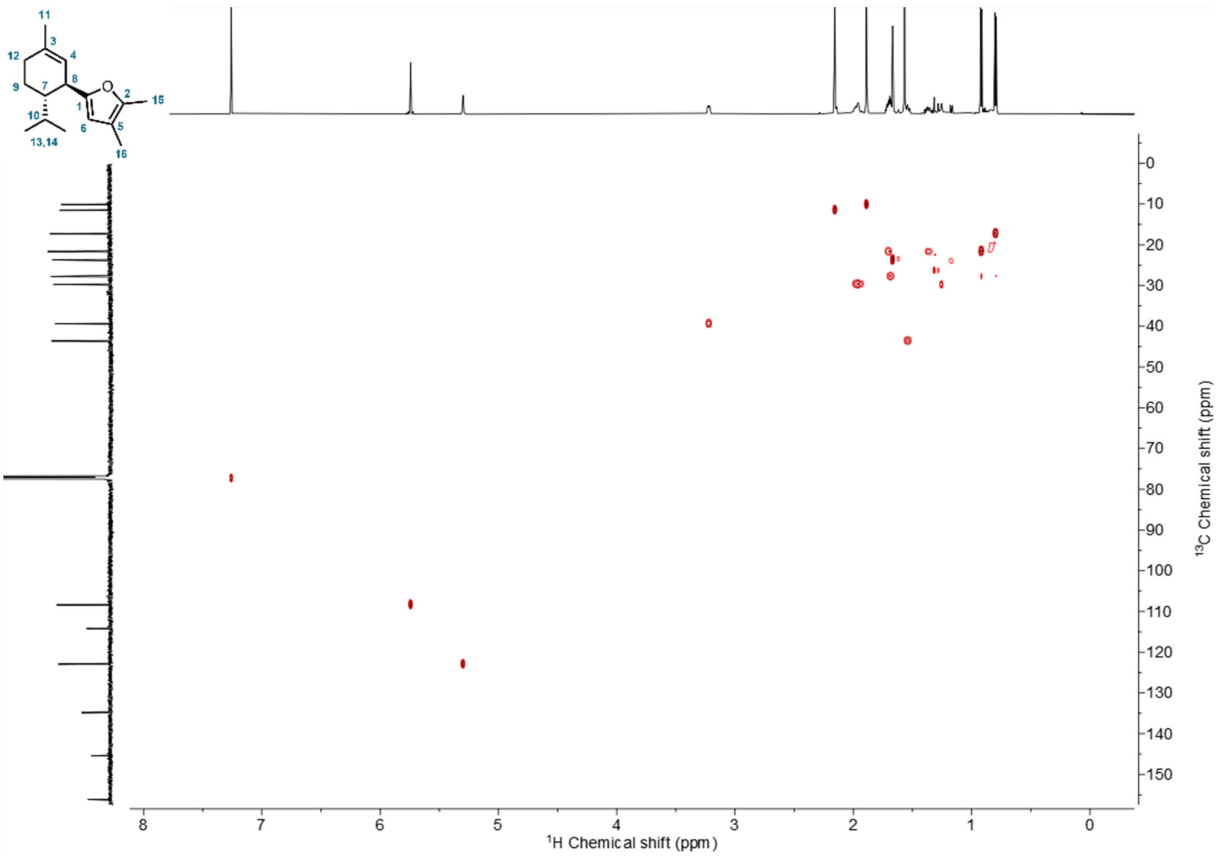


HMBC (600 MHz, $CDCl_3$, 298K)

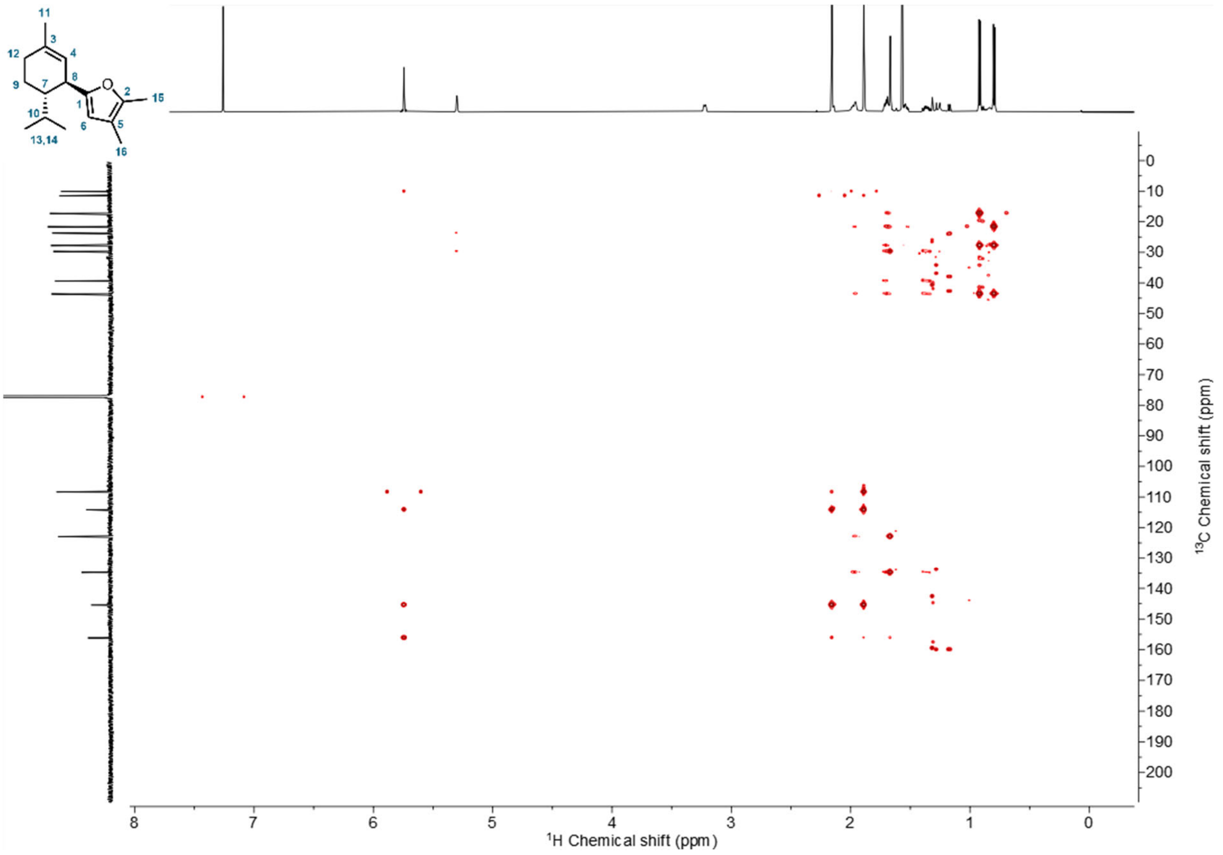

## S15.7 2-(*trans*-*p*-menth-1-en-3-yl)-1,5-dimethoxybenzene 15

$^{1}H$ NMR (600 MHz, $CDCl_3$, 298K)

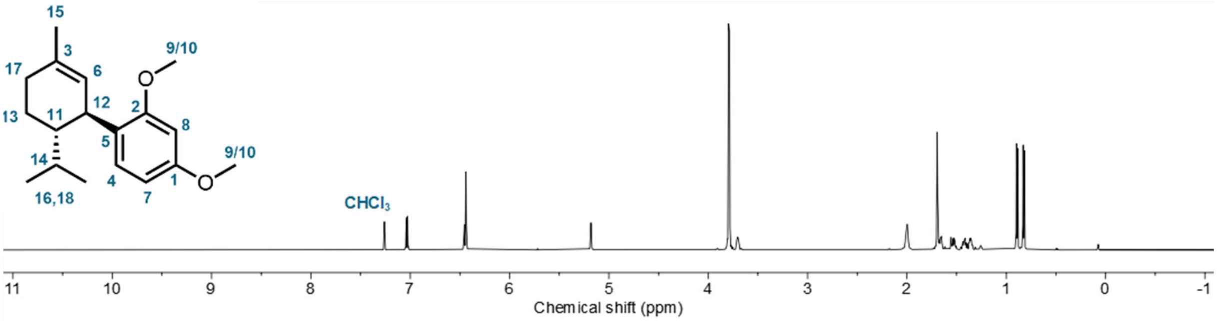


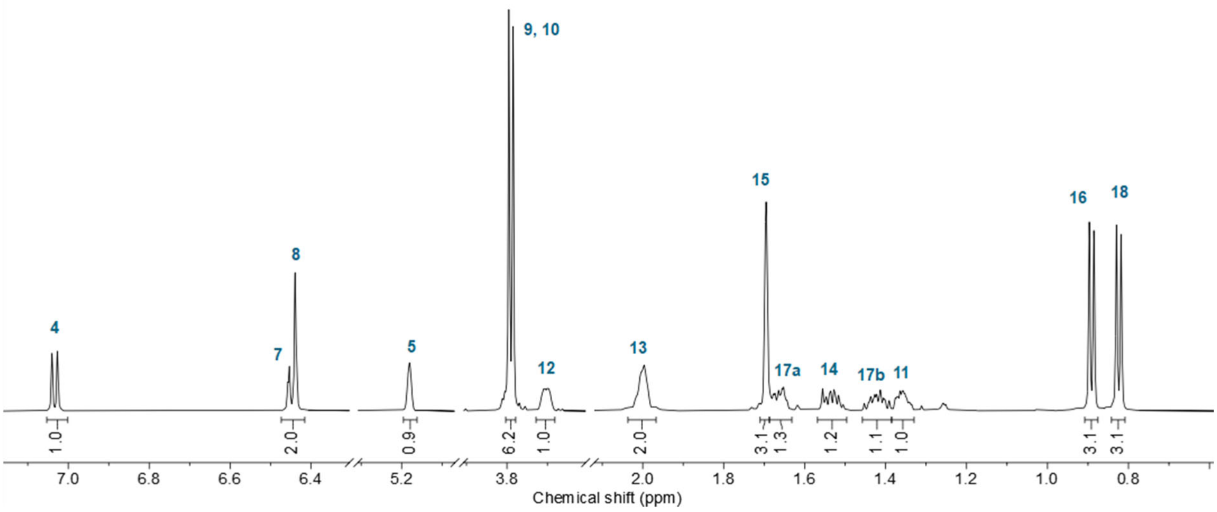


$^{13}C$ NMR (151 MHz, $CDCl_3$, 298K)

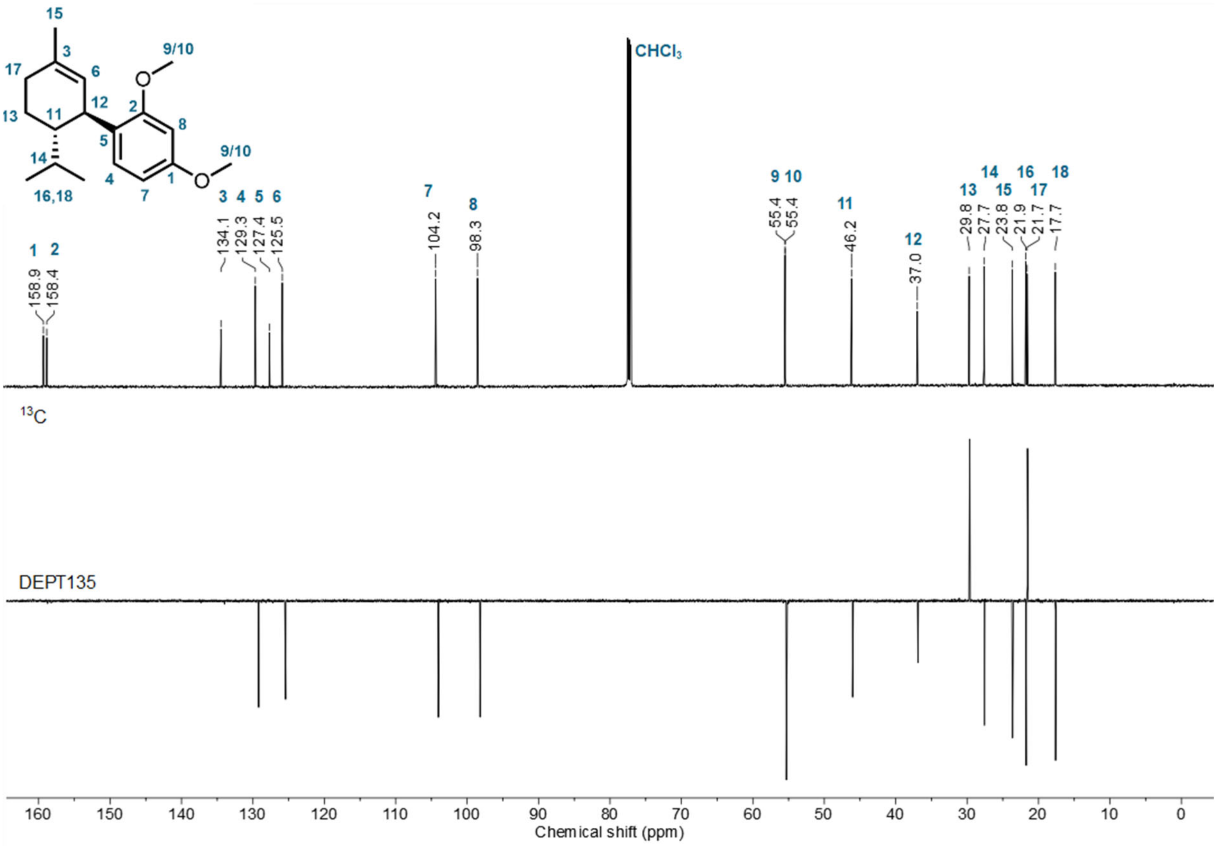

COSY (600 MHz, $CDCl_3$, 298K)

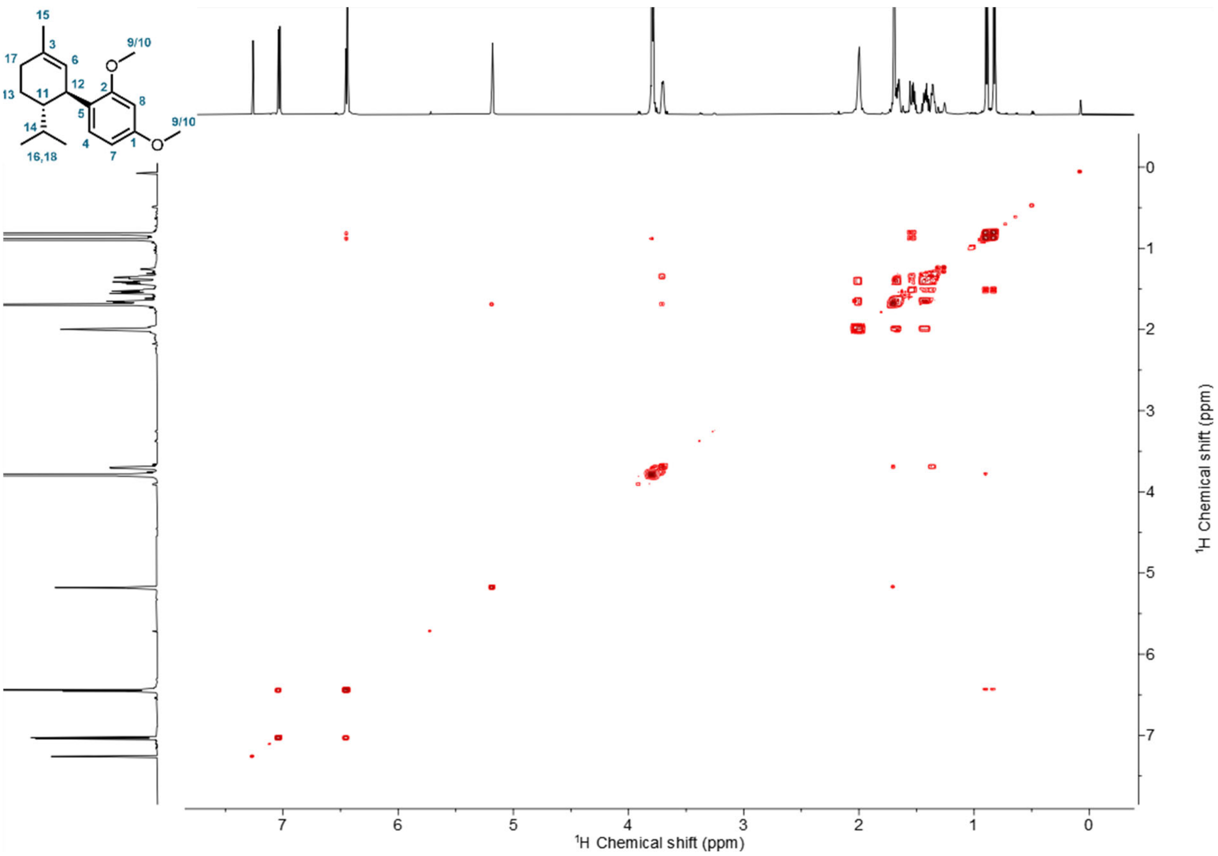


NOESY (600 MHz, $CDCl_3$, 298K)

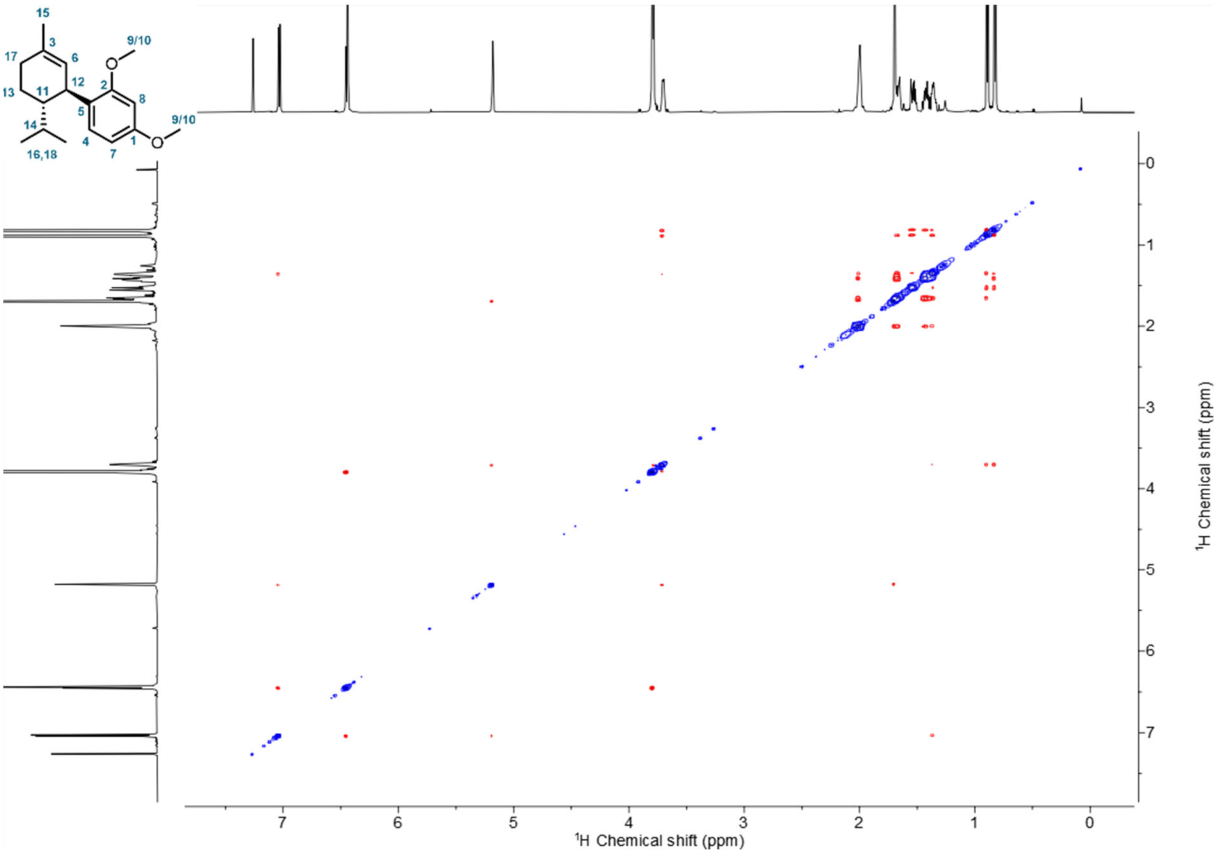

HSQC (600 MHz, $CDCl_3$, 298K)

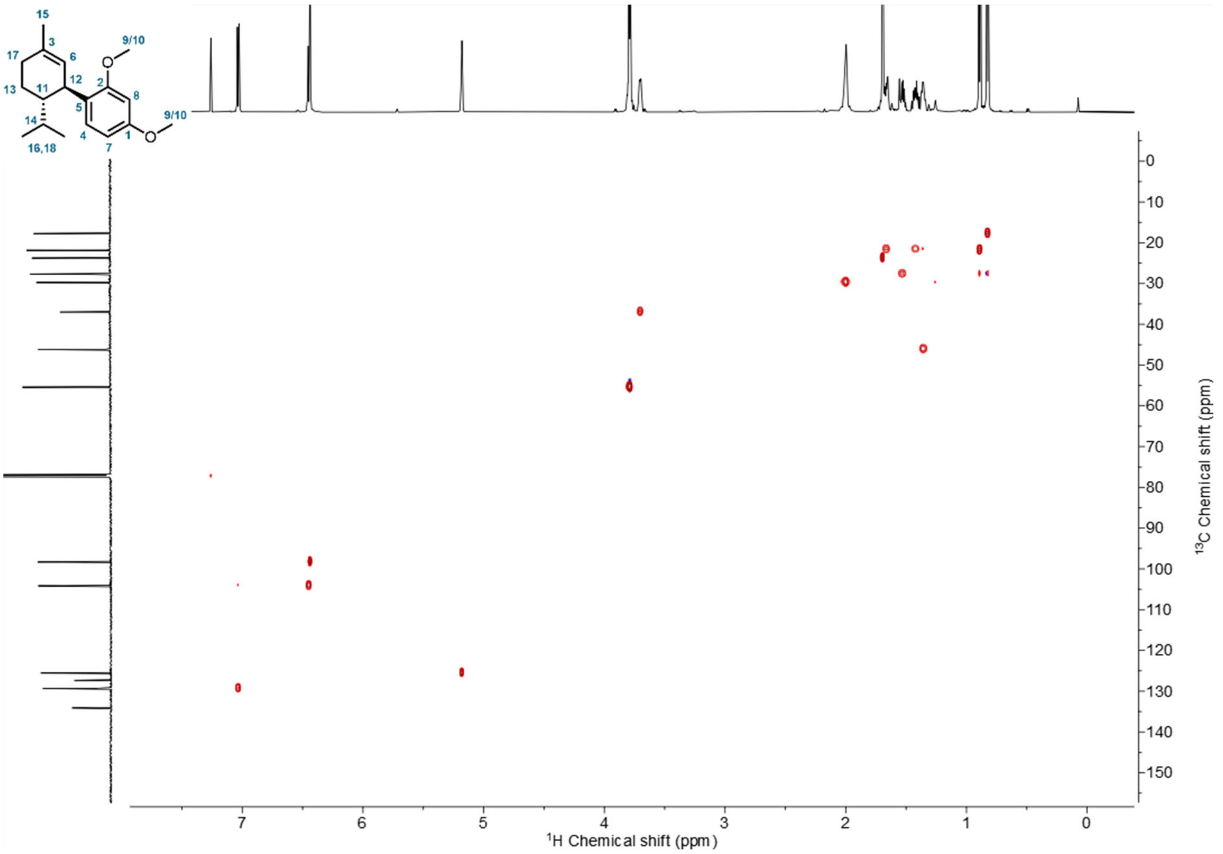


HMBC (600 MHz, $CDCl_3$, 298K)

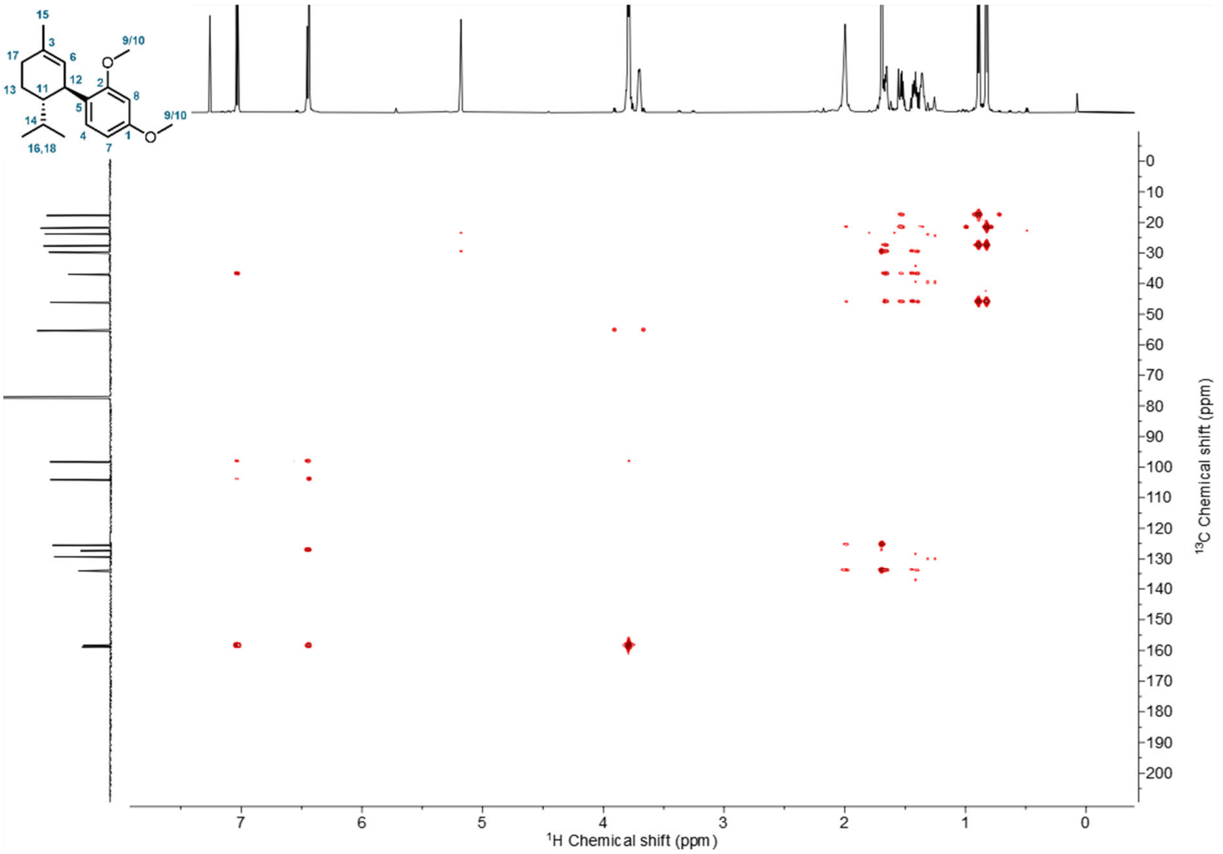

## S15.8 Cyclic ether 16

$^{1}H$ NMR (600 MHz, $CDCl_3$, 298K)

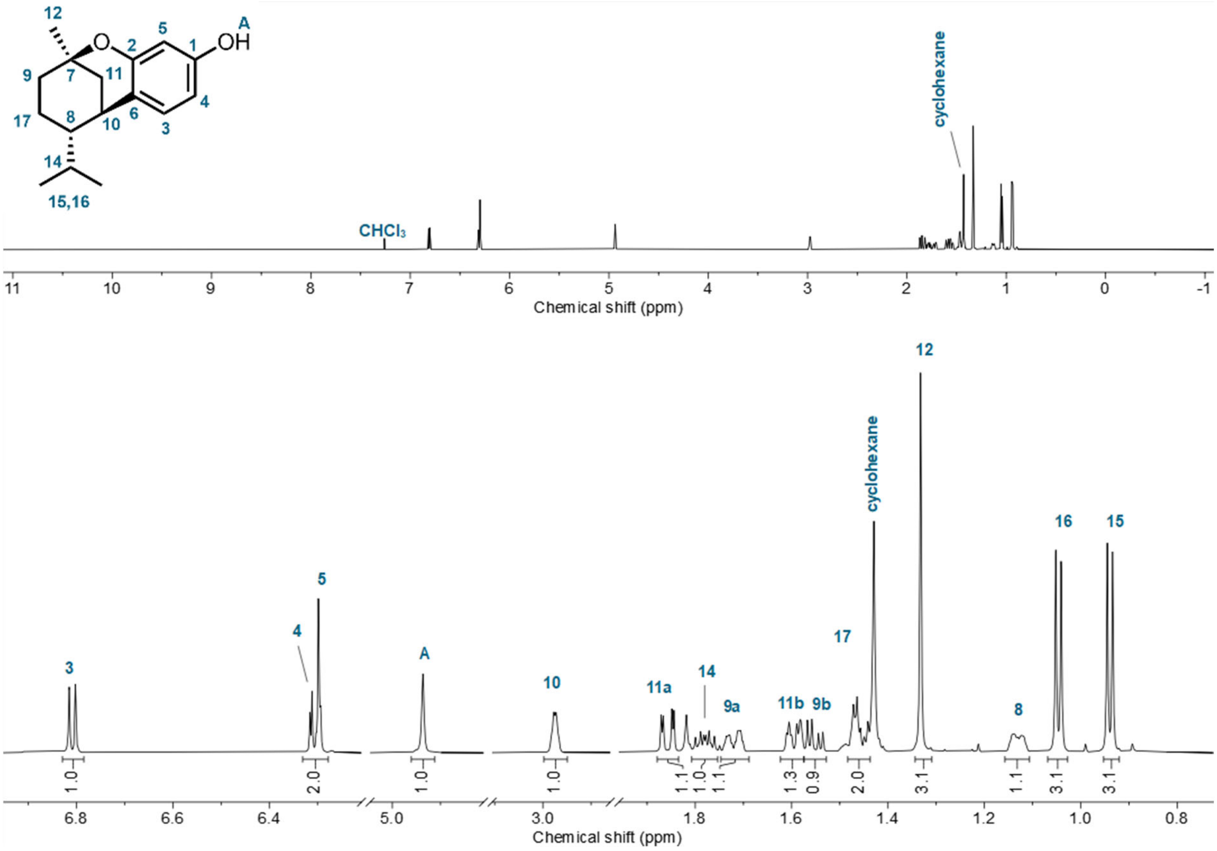


$^{13}C$ NMR (151 MHz, $CDCl_3$, 298K)

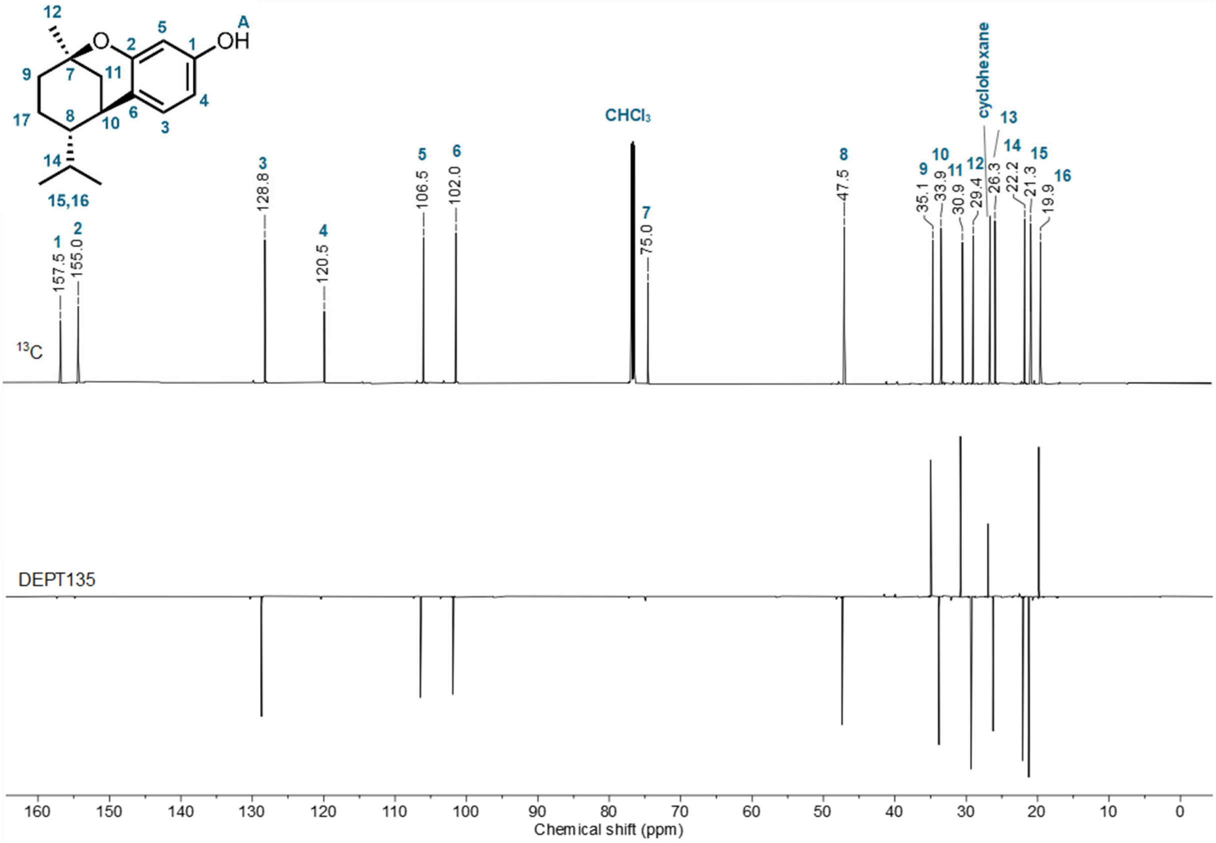

COSY (600 MHz, $CDCl_3$, 298K)

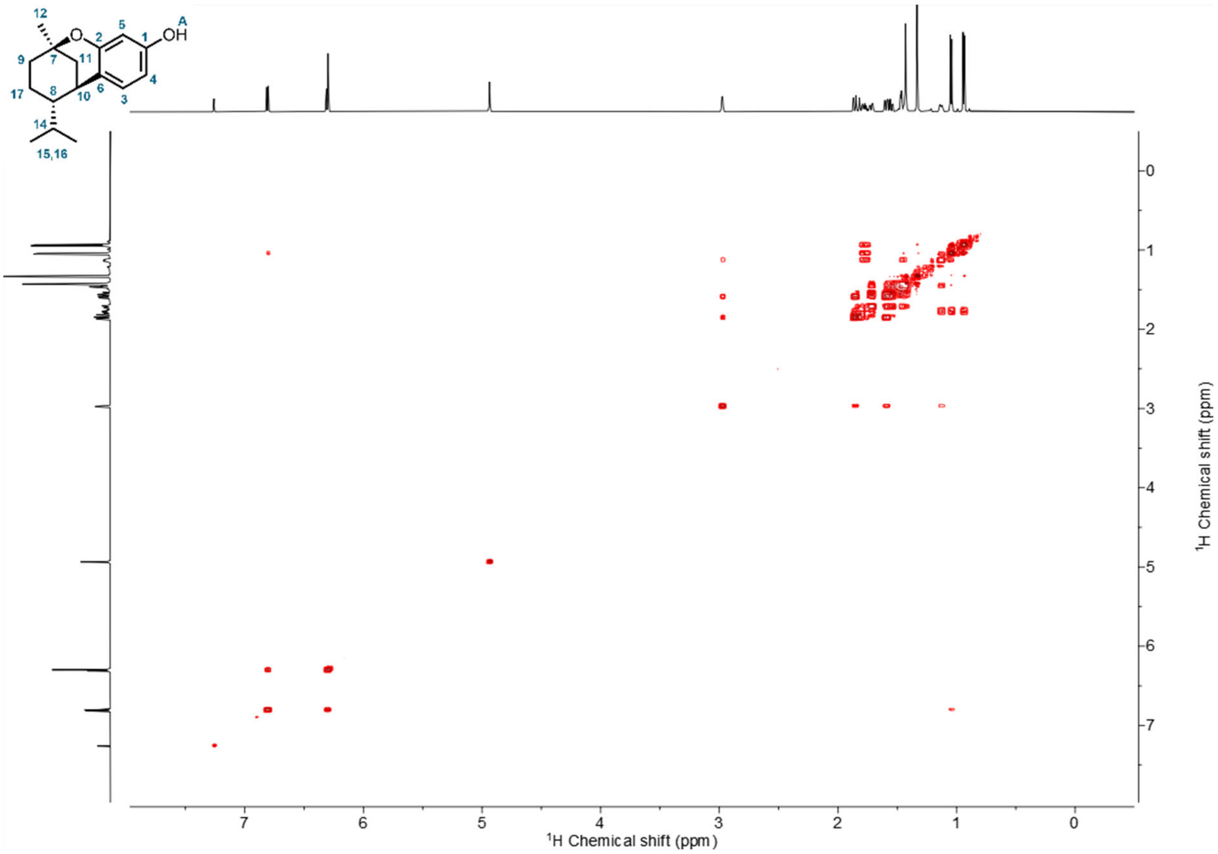


NOESY (600 MHz, $CDCl_3$, 298K)

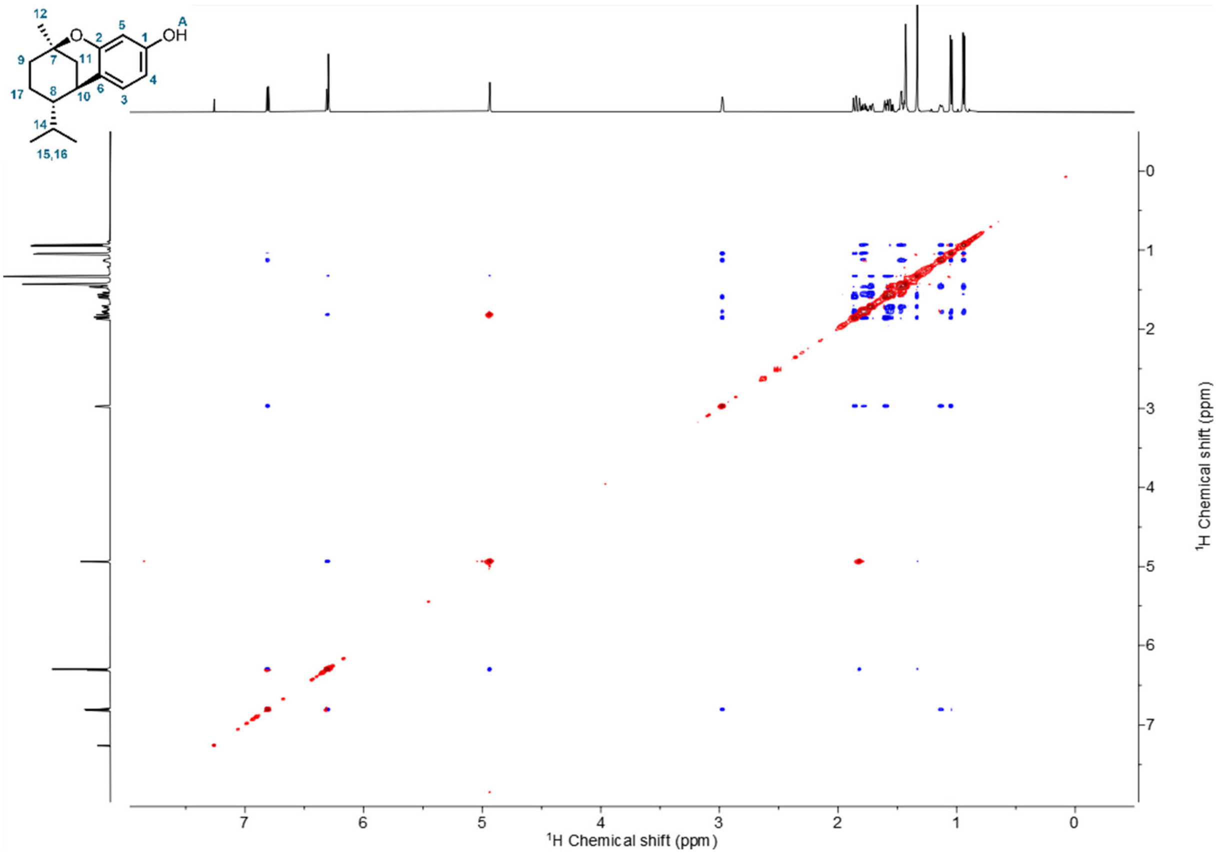

HSQC (600 MHz, $CDCl_3$, 298K)

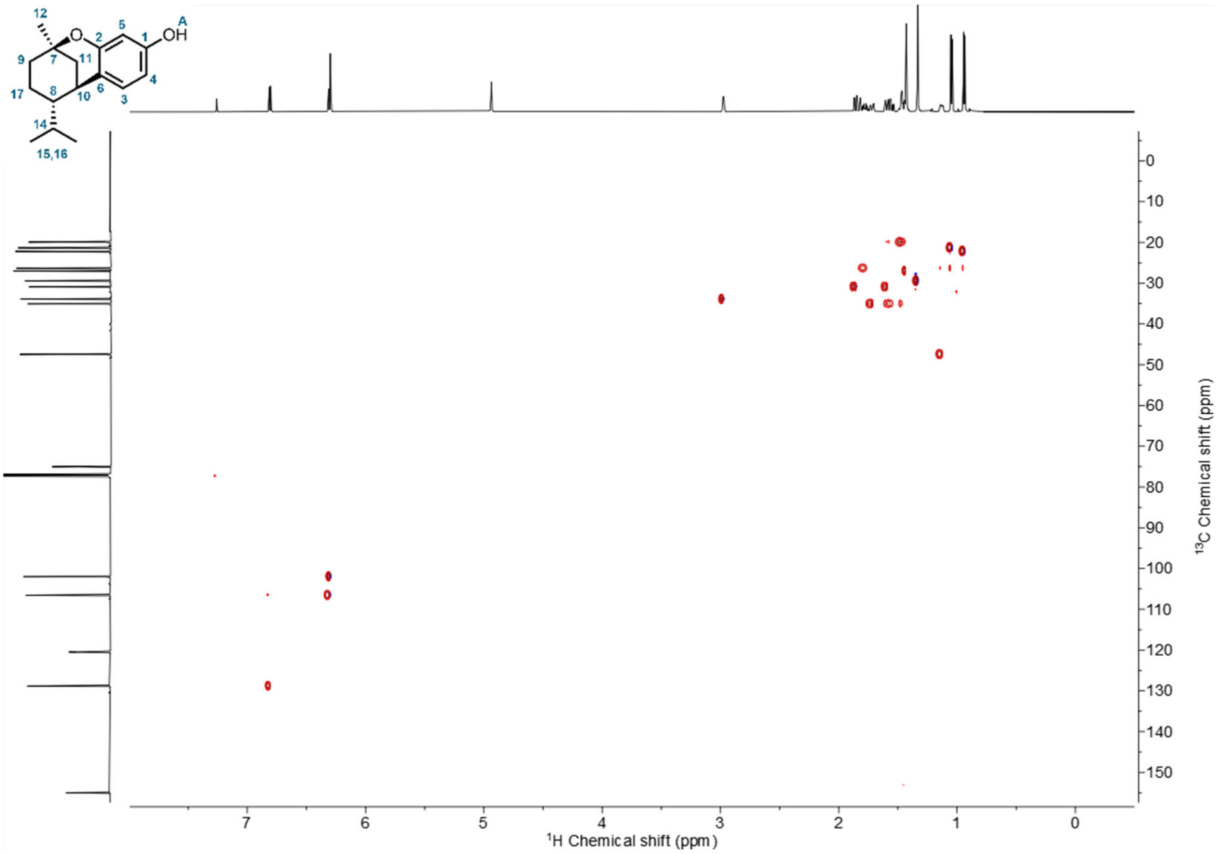


HMBC (600 MHz, $CDCl_3$, 298K)

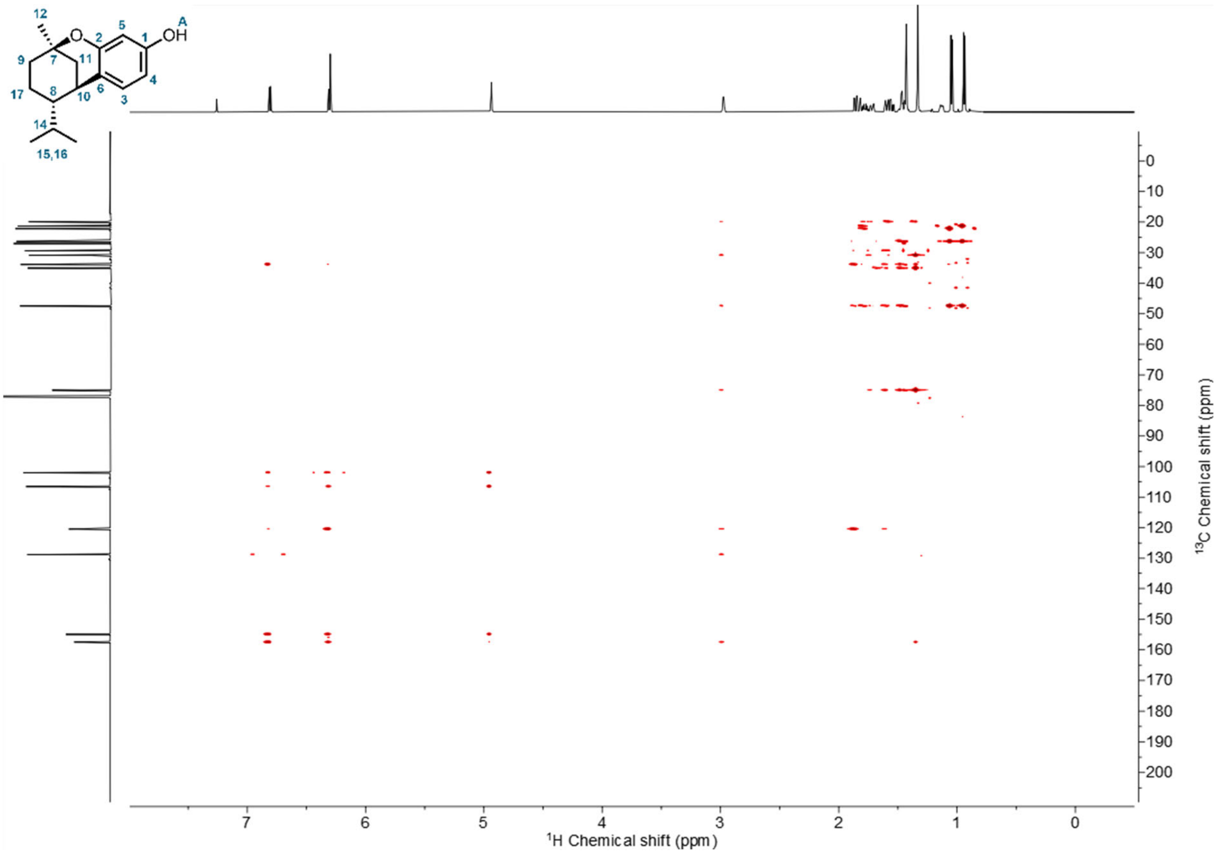

## S15.9 2-(*trans*-*p*-menth-1-en-3-yl)-1-methylpyrrole 17

$^{1}H$ NMR (600 MHz, $CDCl_3$, 298K)

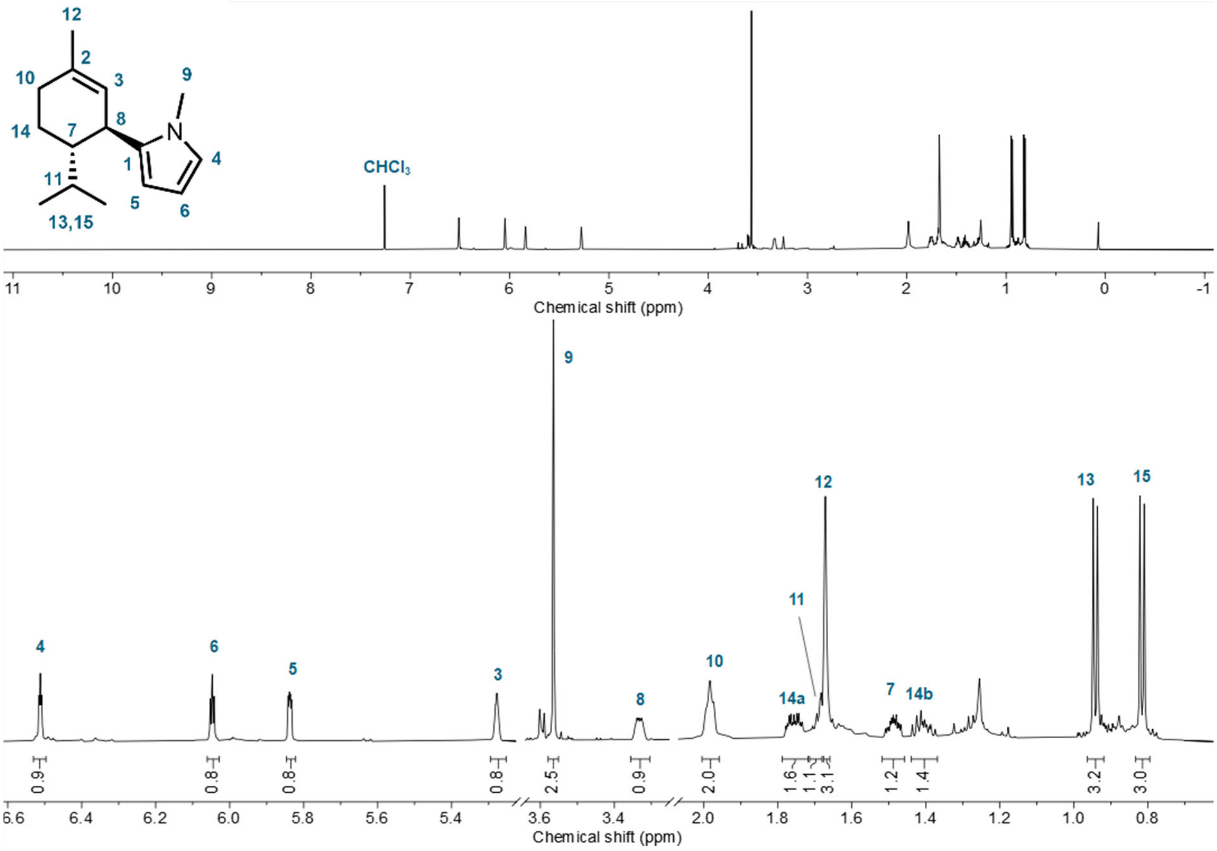


$^{13}C$ NMR (151 MHz, $CDCl_3$, 298K)

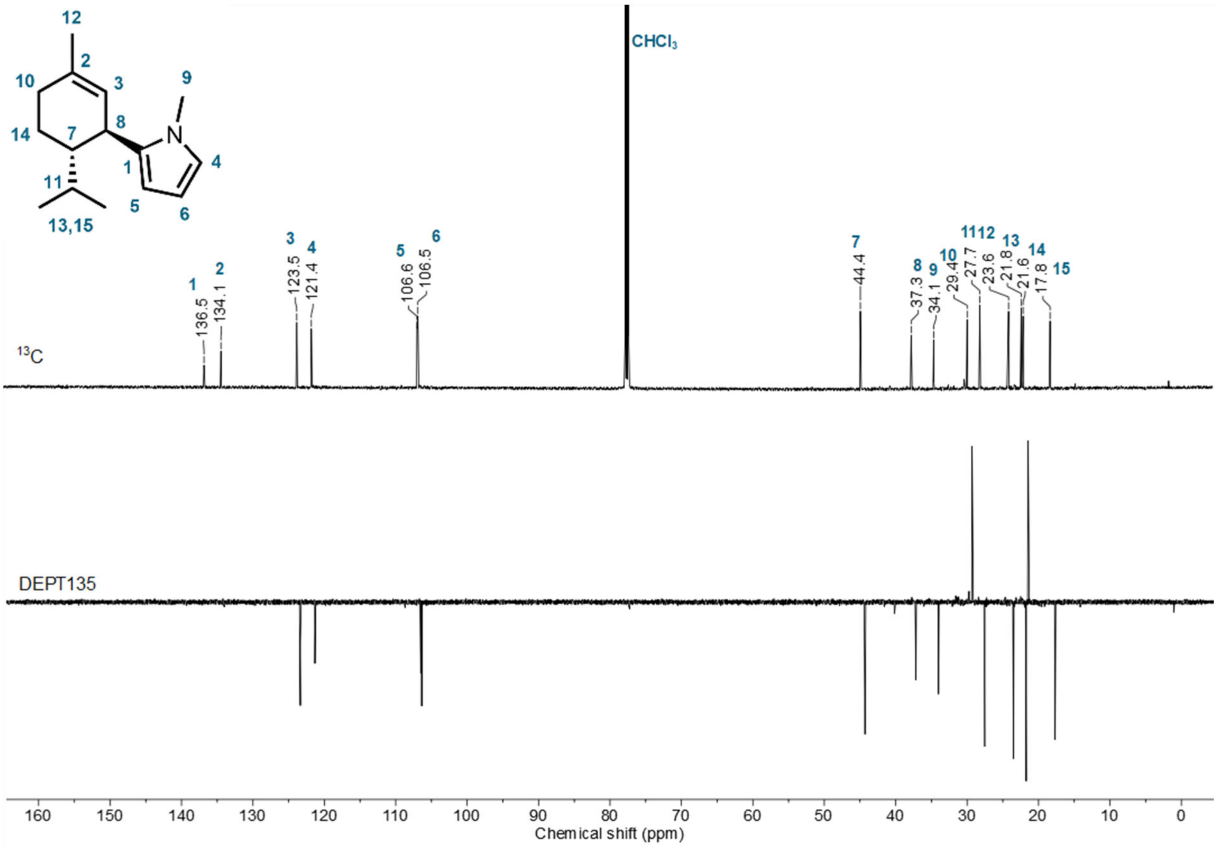

COSY (600 MHz, $CDCl_3$, 298K)

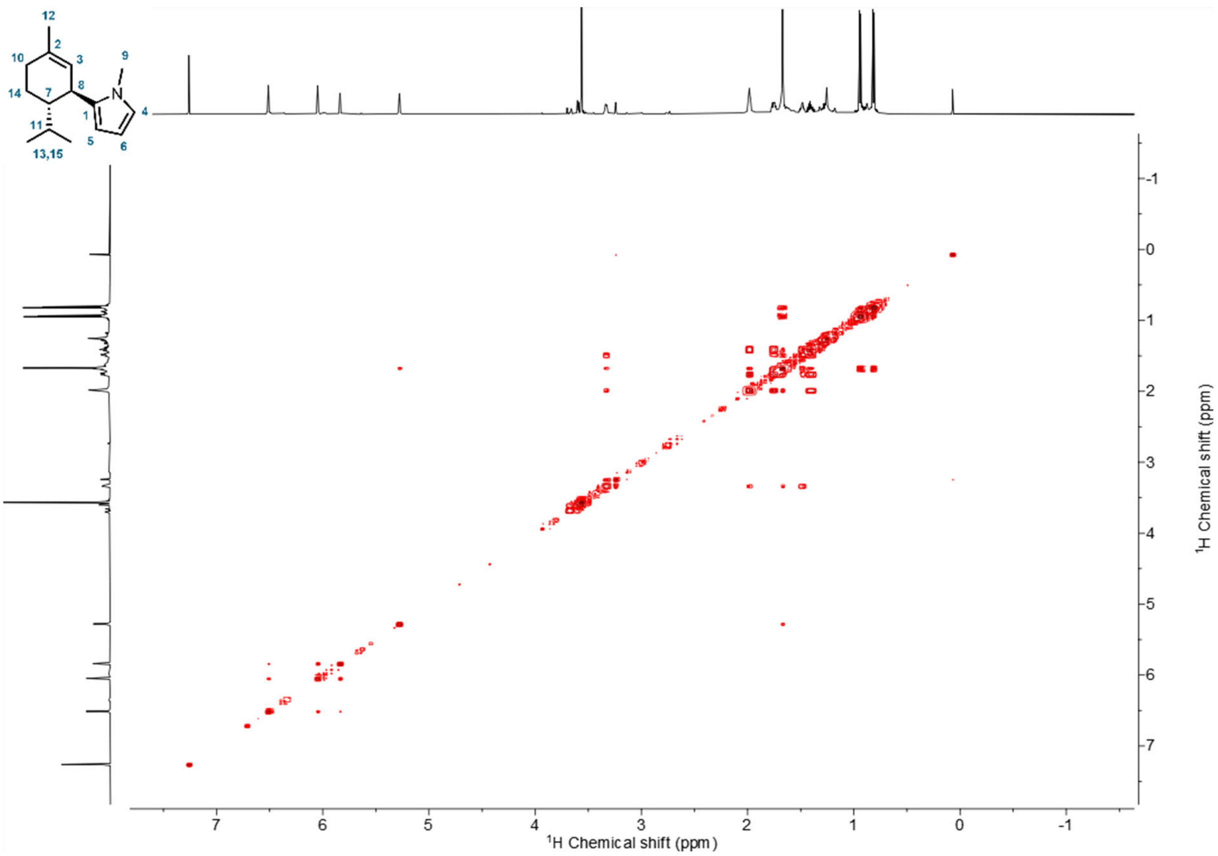


NOESY (600 MHz, $CDCl_3$, 298K)

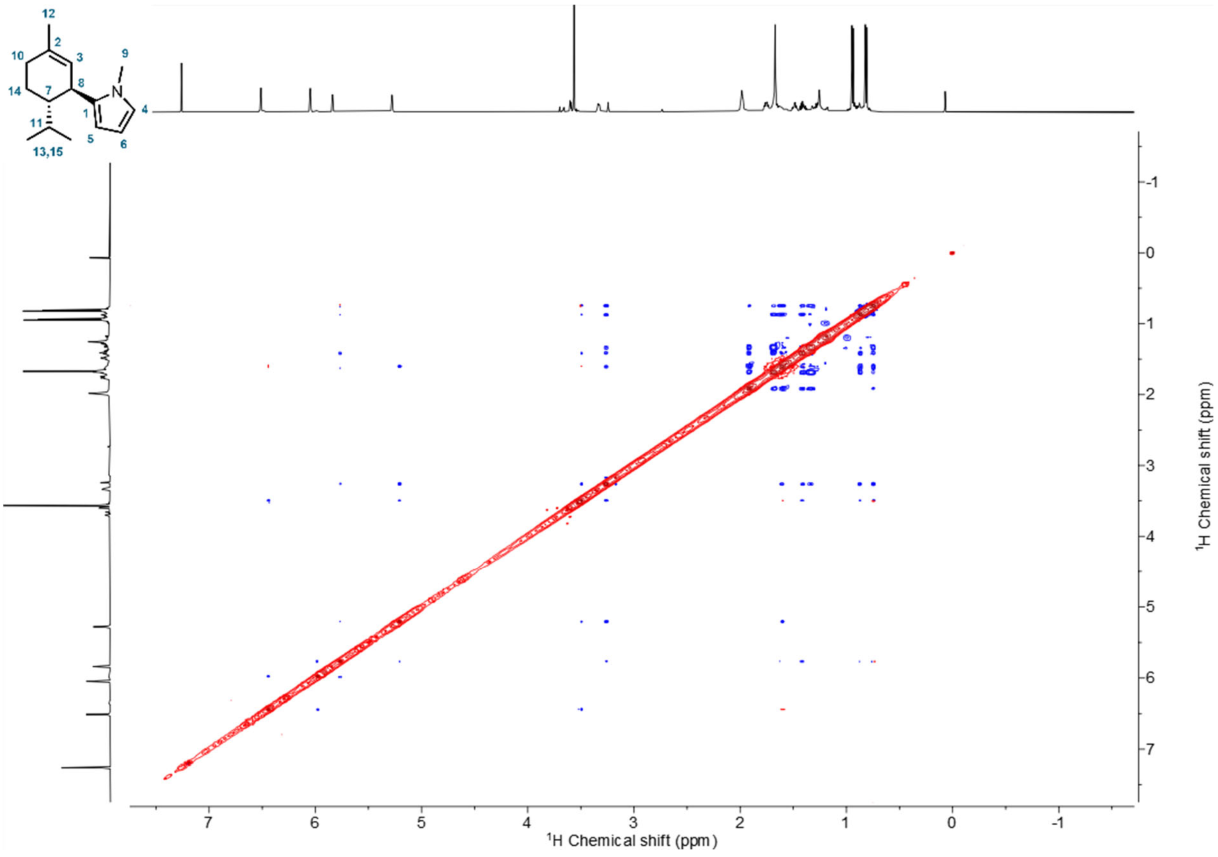

HSQC (600 MHz, $CDCl_3$, 298K)

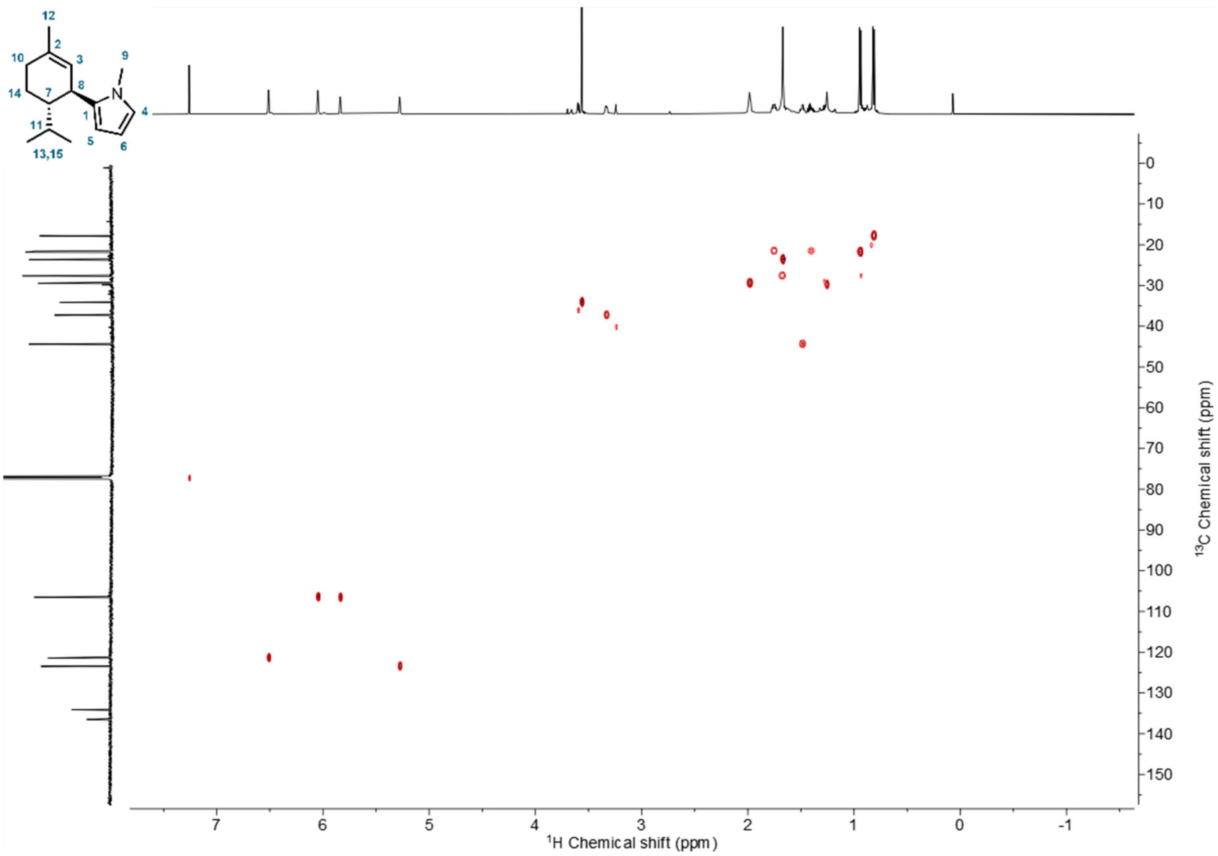


HMBC (600 MHz, $CDCl_3$, 298K)

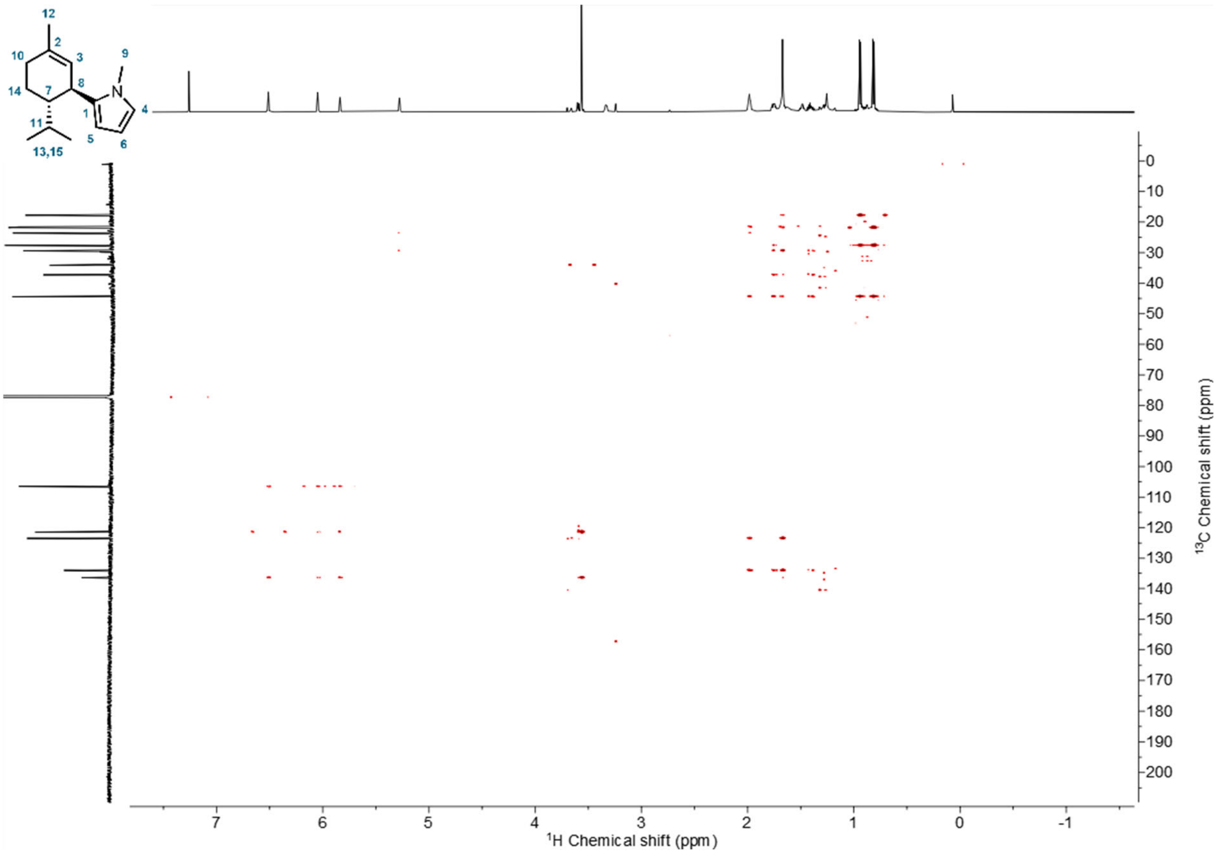

## S15.10 1-presilphiperfolanylpentane-1,4-dione 18

$^{1}H$ NMR (600 MHz, $CDCl_3$, 298K)

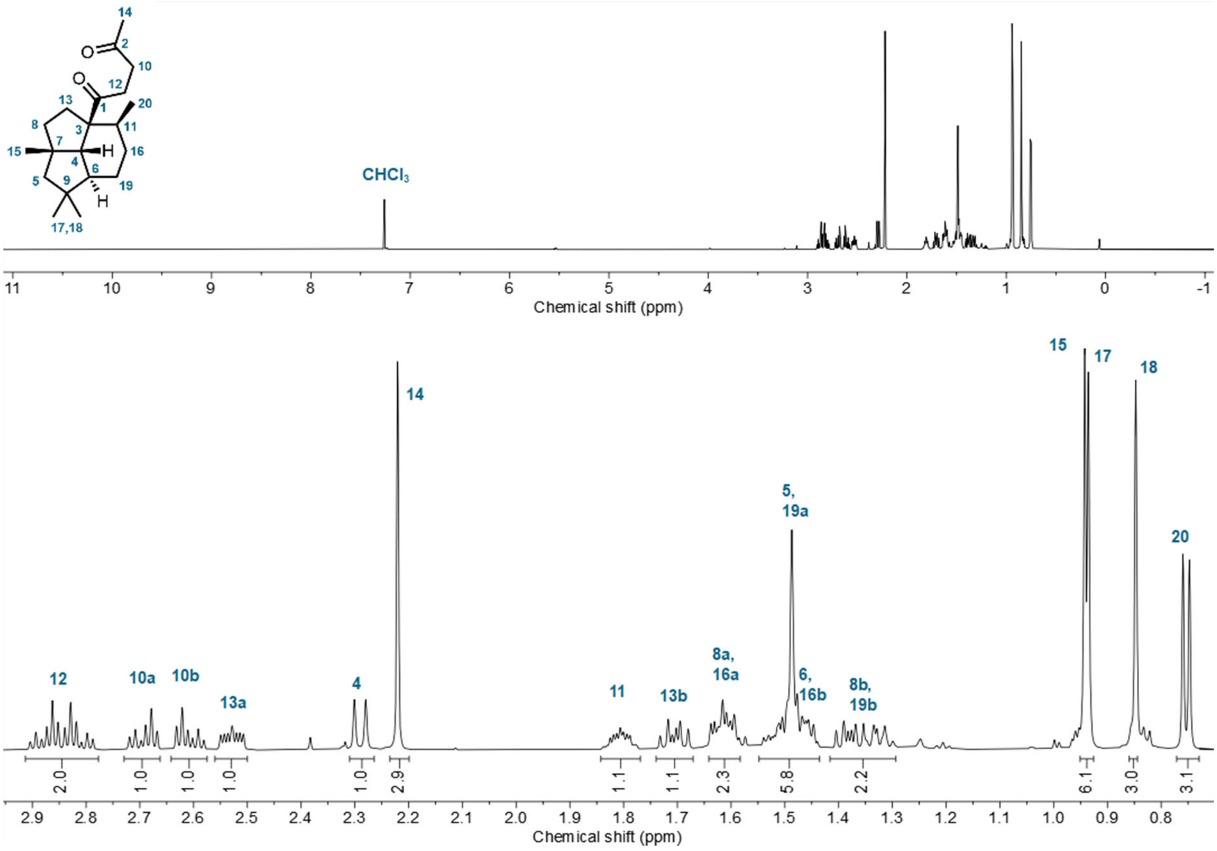


$^{13}C$ NMR (151 MHz, $CDCl_3$, 298K)

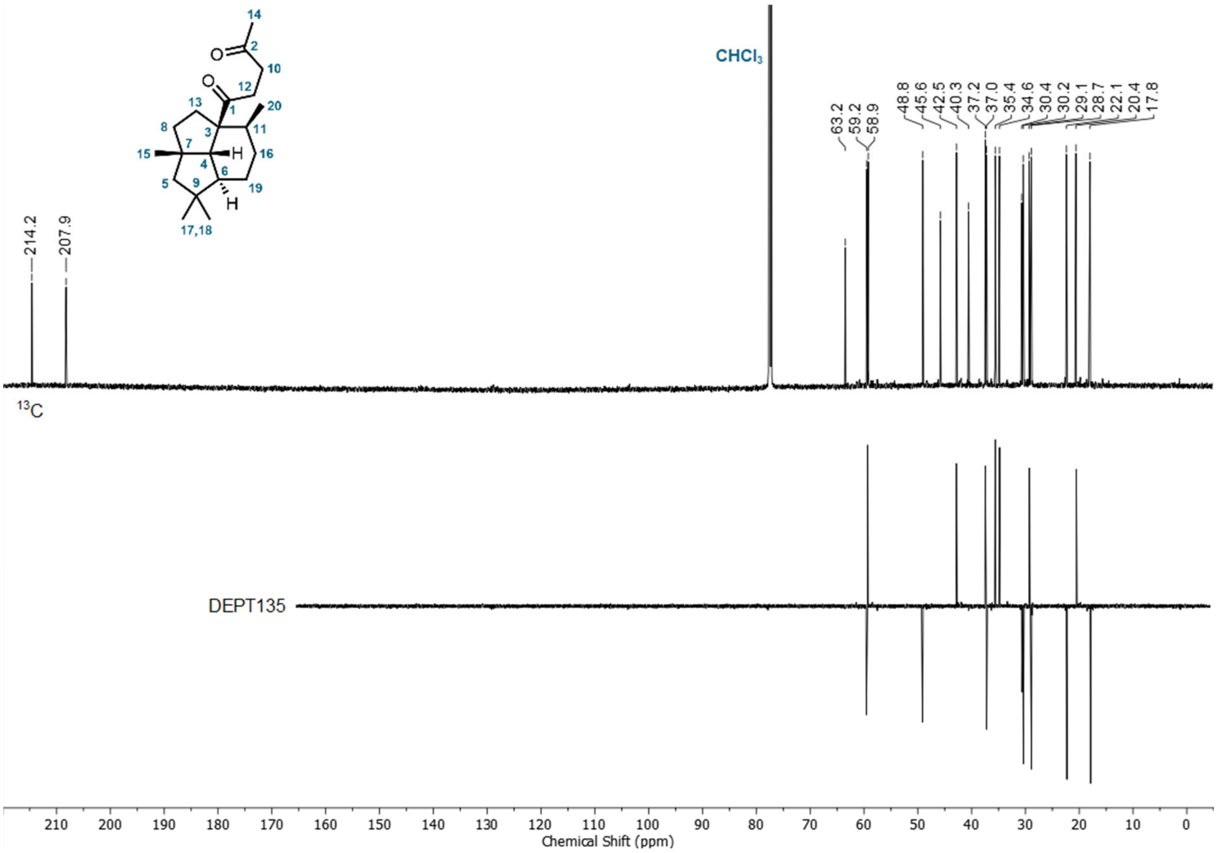

COSY (600 MHz, $CDCl_3$, 298K)

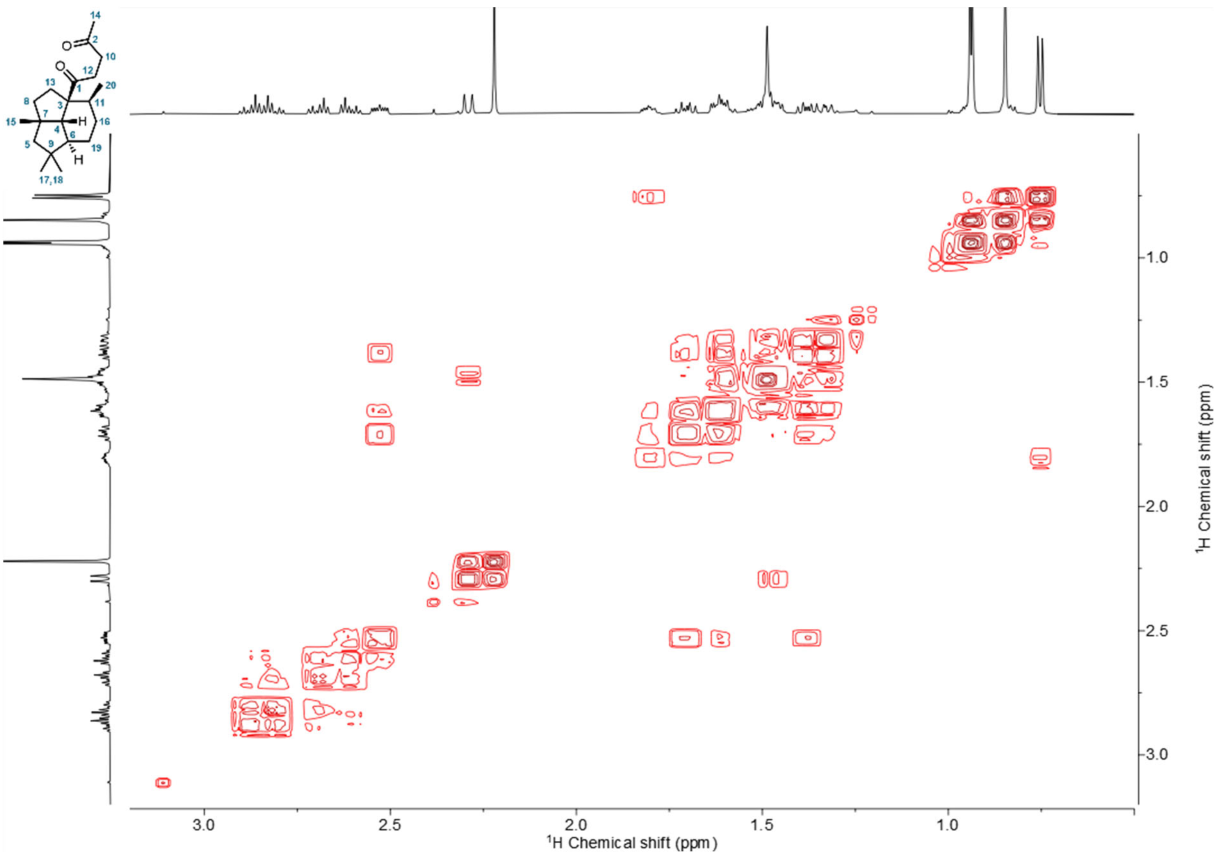


NOESY (600 MHz, $CDCl_3$, 298K)

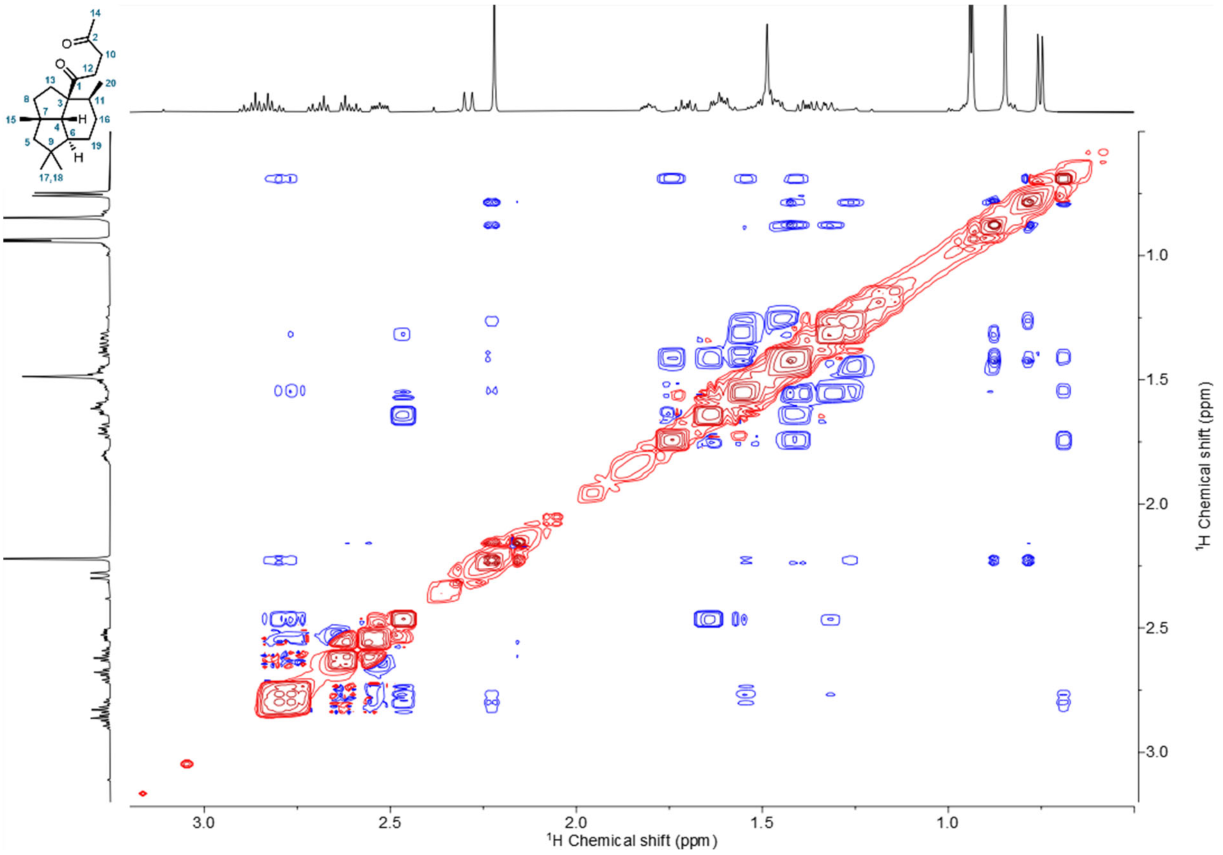

HSQC (600 MHz, $CDCl_3$, 298K)

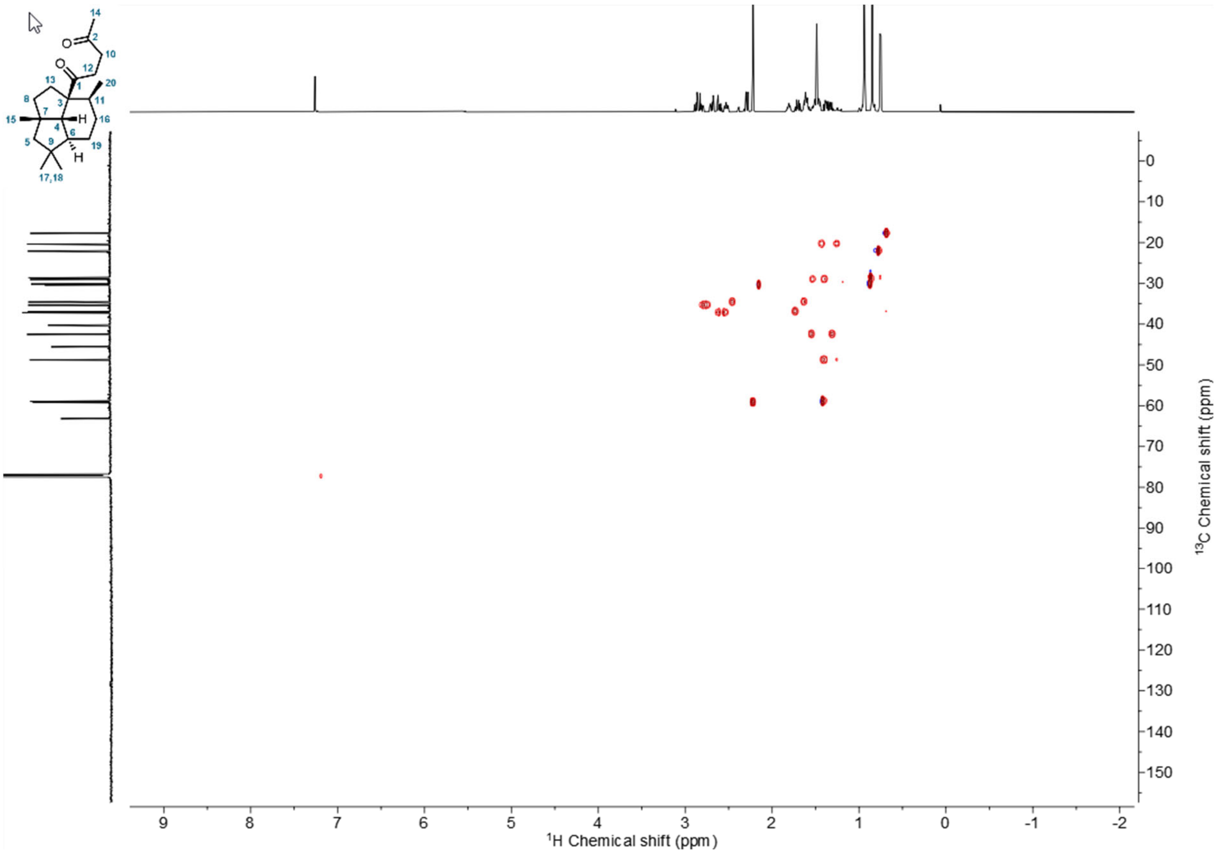

HMBC (600 MHz, $CDCl_3$, 298K)

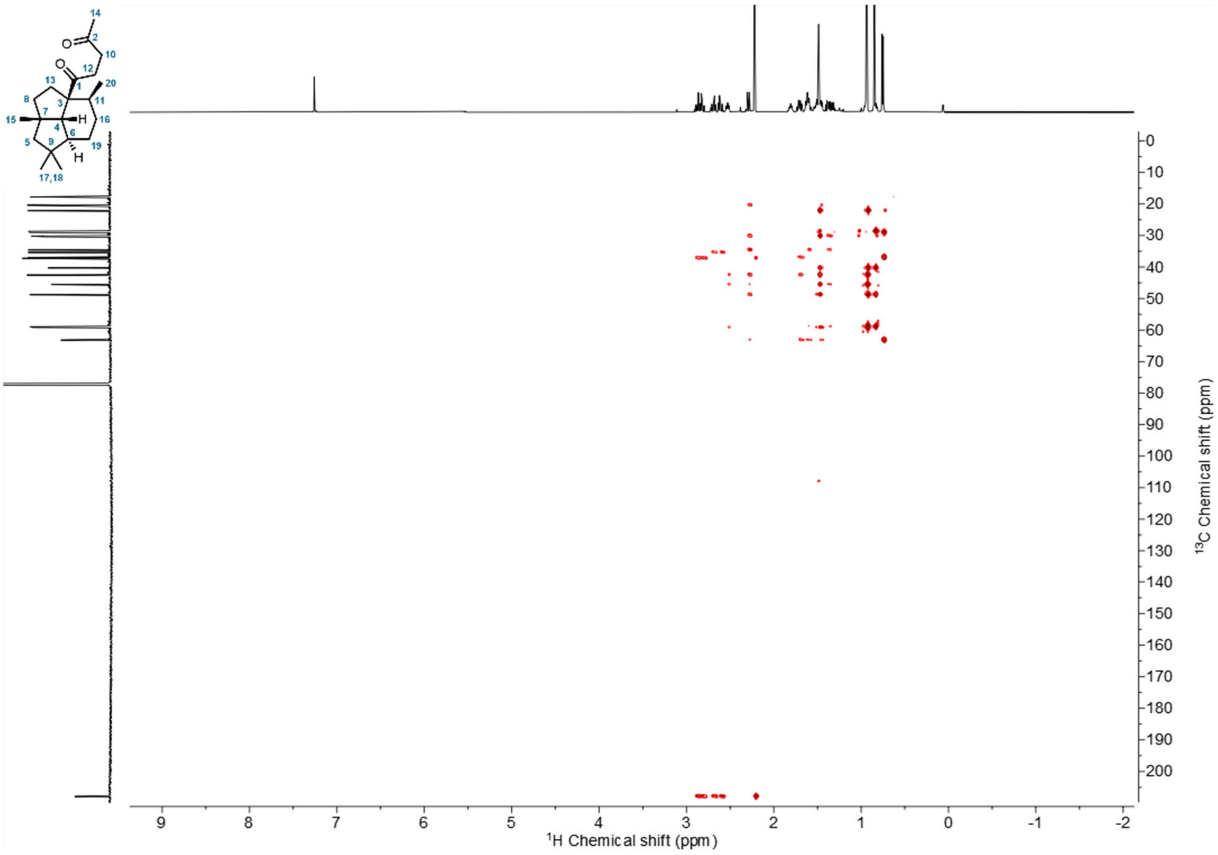

## S15.11 2-presilphiperfolanyl-1-methylpyrrole 19

$^{1}H$ NMR (600 MHz, $CDCl_3$, 298K)

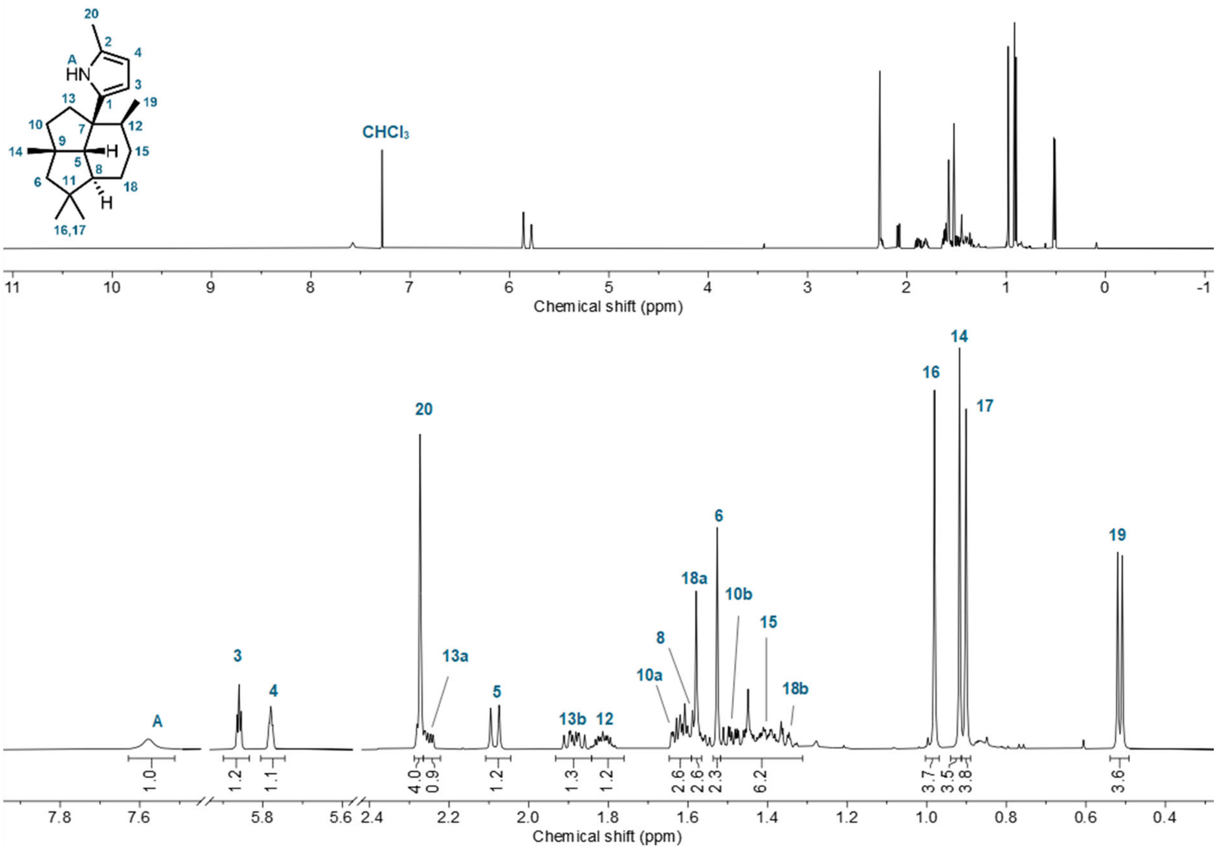


$^{13}C$ NMR (151 MHz, $CDCl_3$, 298K)

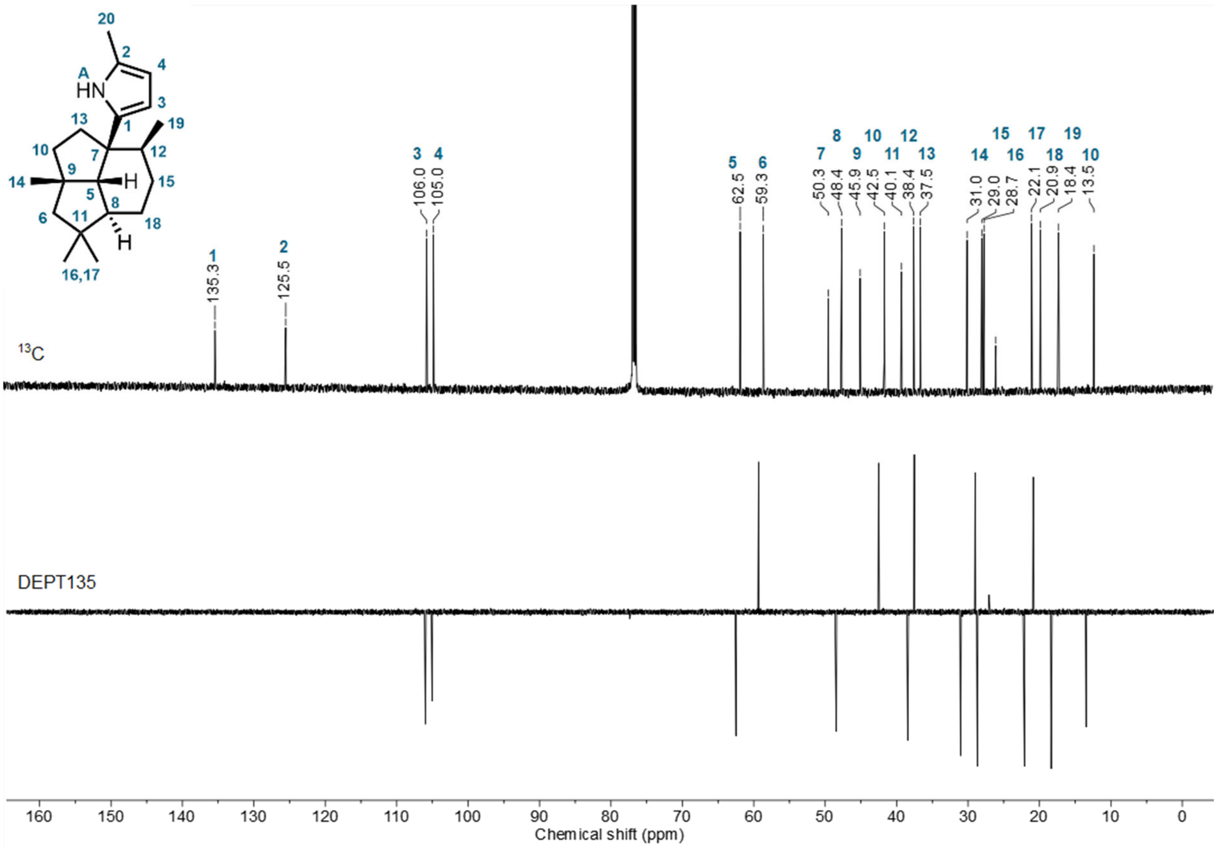

COSY (600 MHz, $CDCl_3$, 298K)

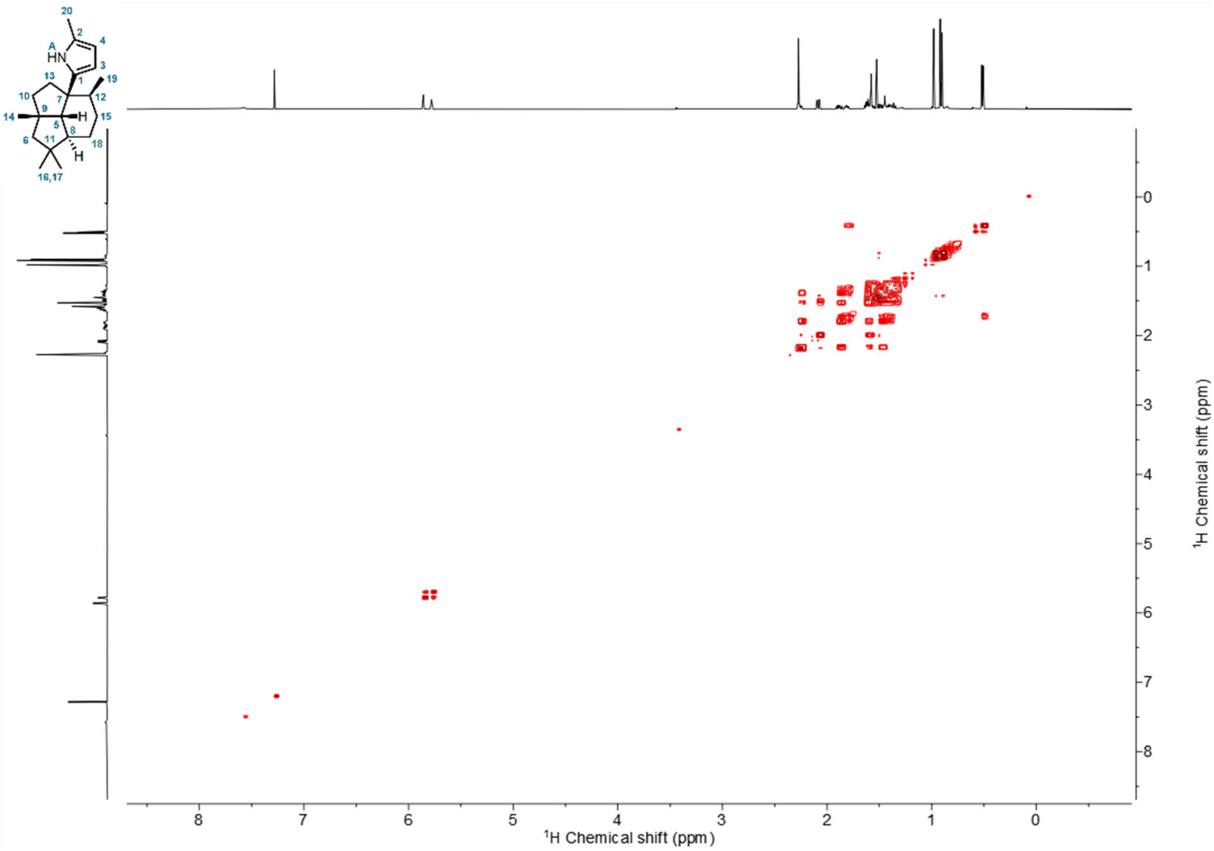


NOESY (600 MHz, $CDCl_3$, 298K)

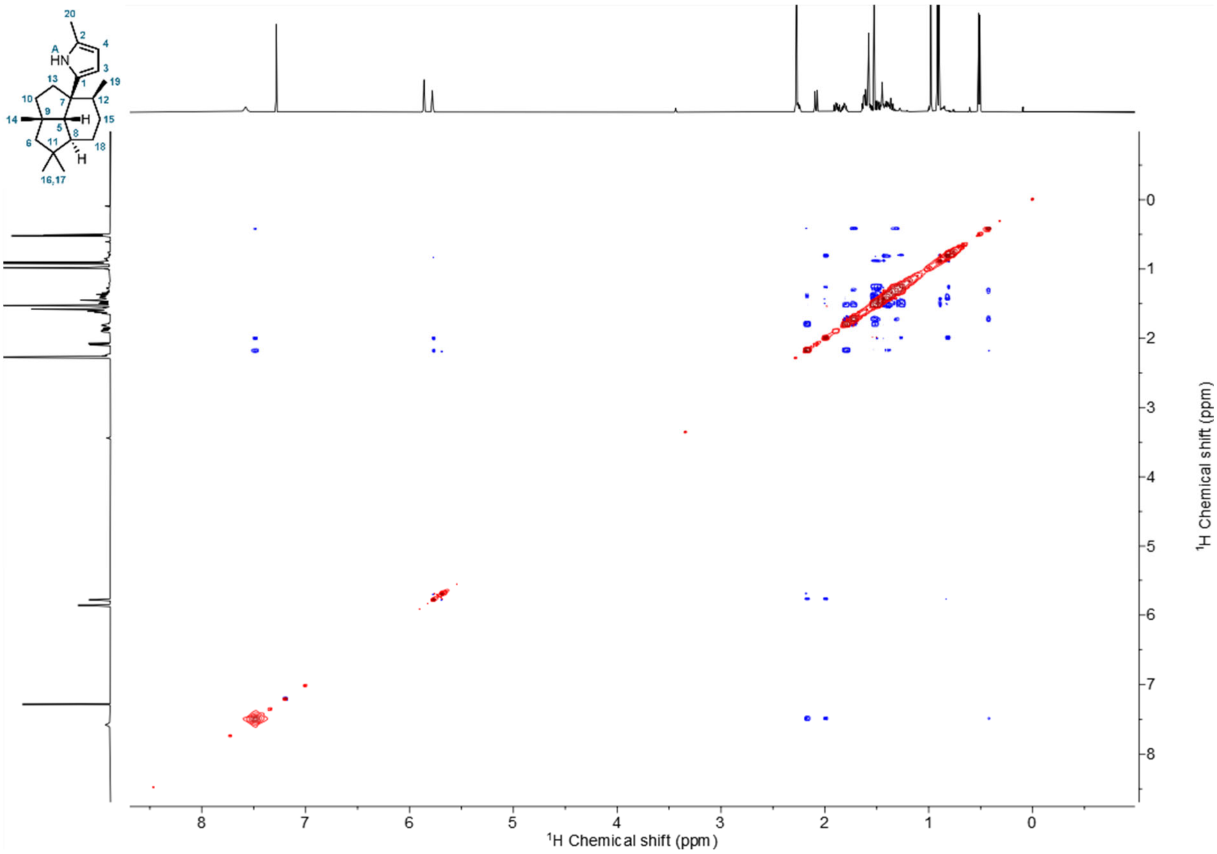

HSQC (600 MHz, CDCl$_3$, 298K)

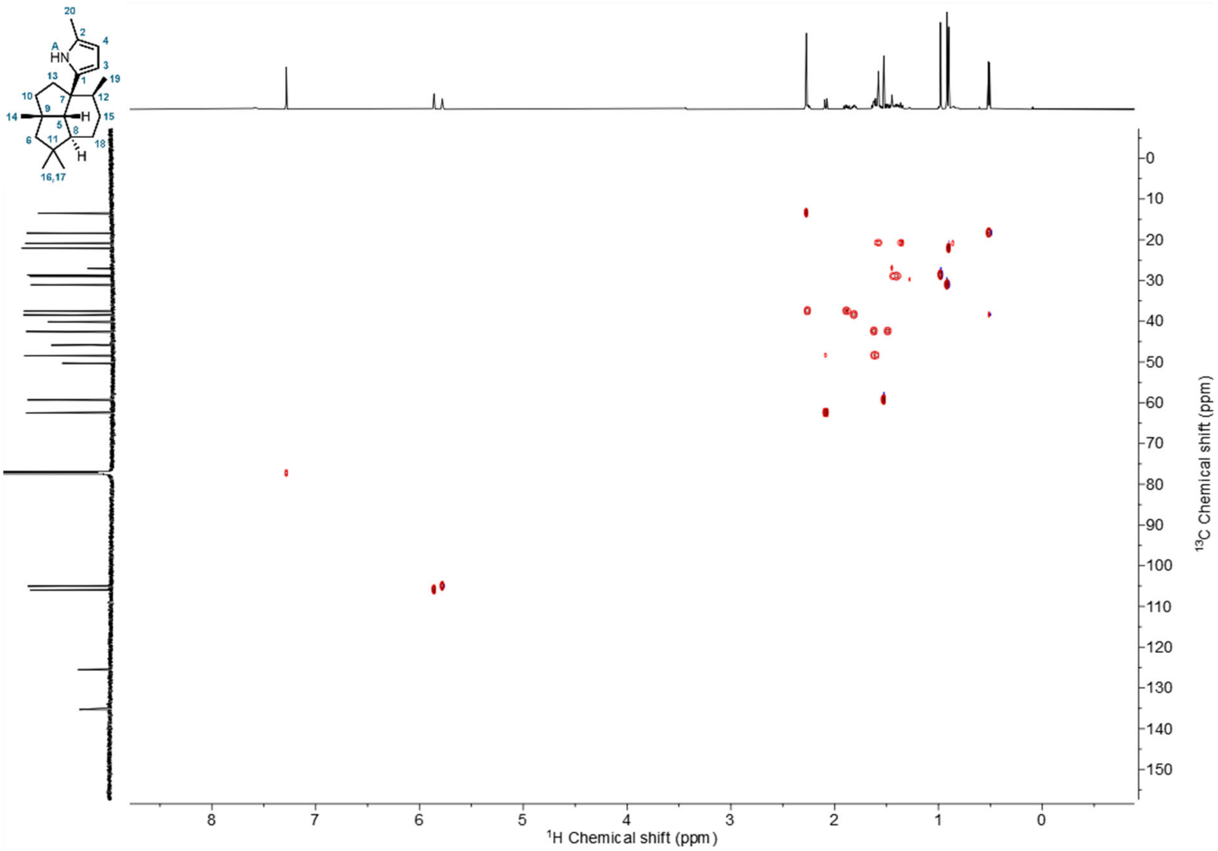


HMBC (600 MHz, CDCl$_3$, 298K)

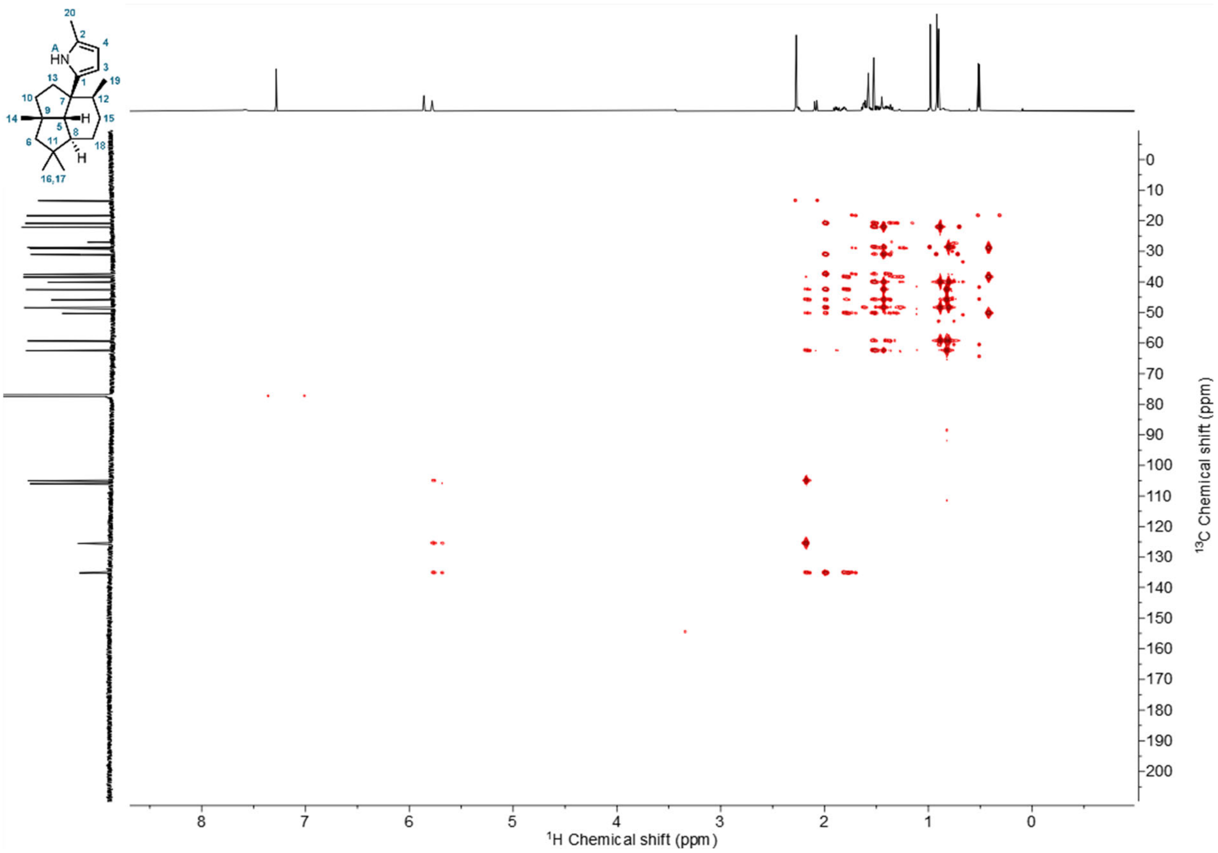

1,1 ADEQUATE (600 MHz, $CDCl_3$, 298K)

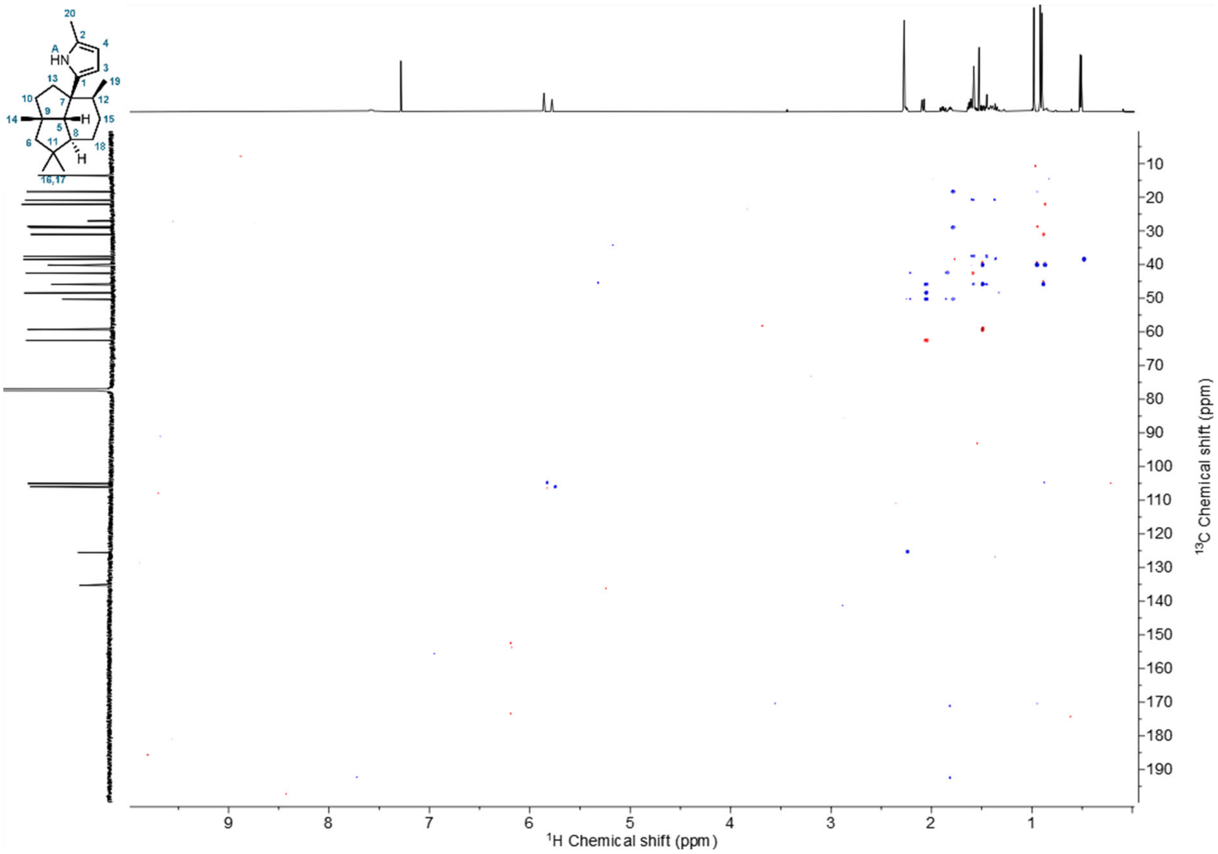

## S15.12 (2Z)-1-presilphiperfolanylpent-2-ene-1,4-dione 20

$^{1}$H NMR (600 MHz, $CDCl_3$, 298K)

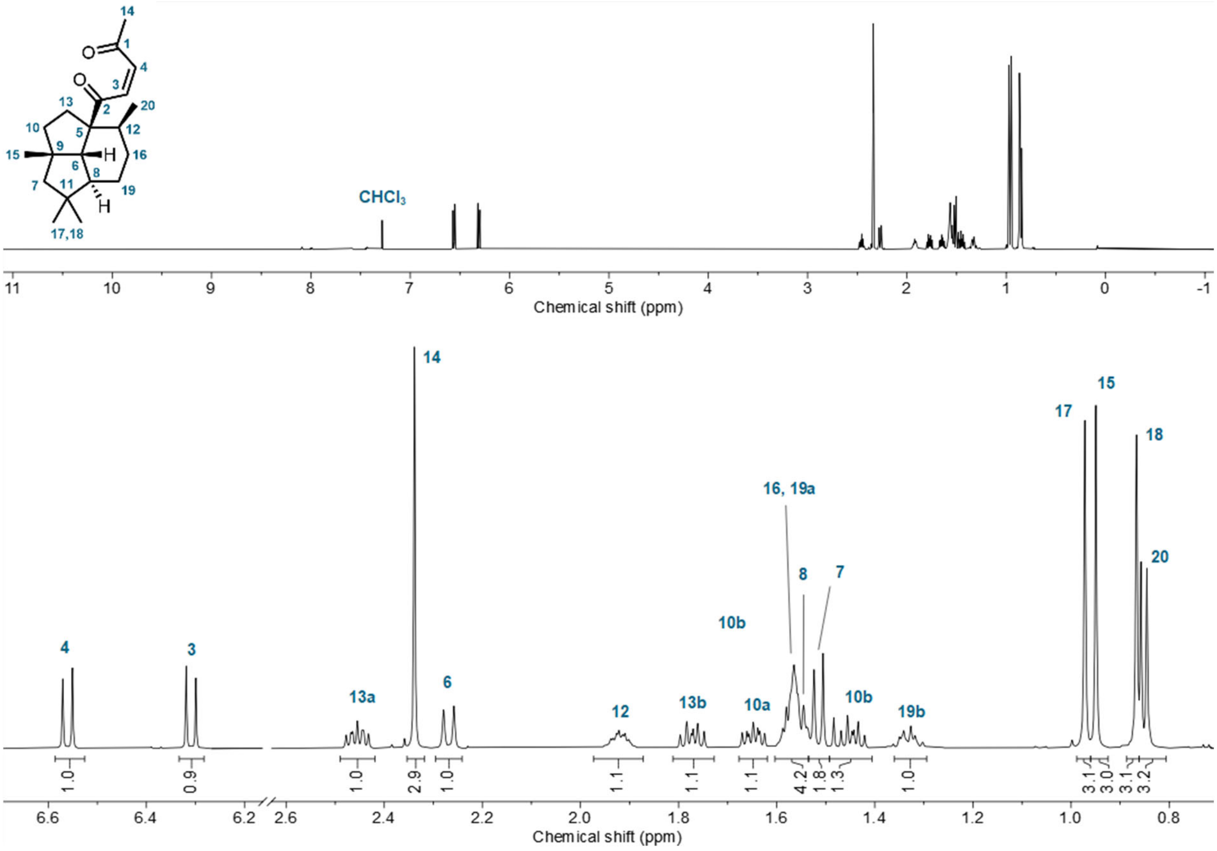


$^{13}$C NMR (151 MHz, $CDCl_3$, 298K)

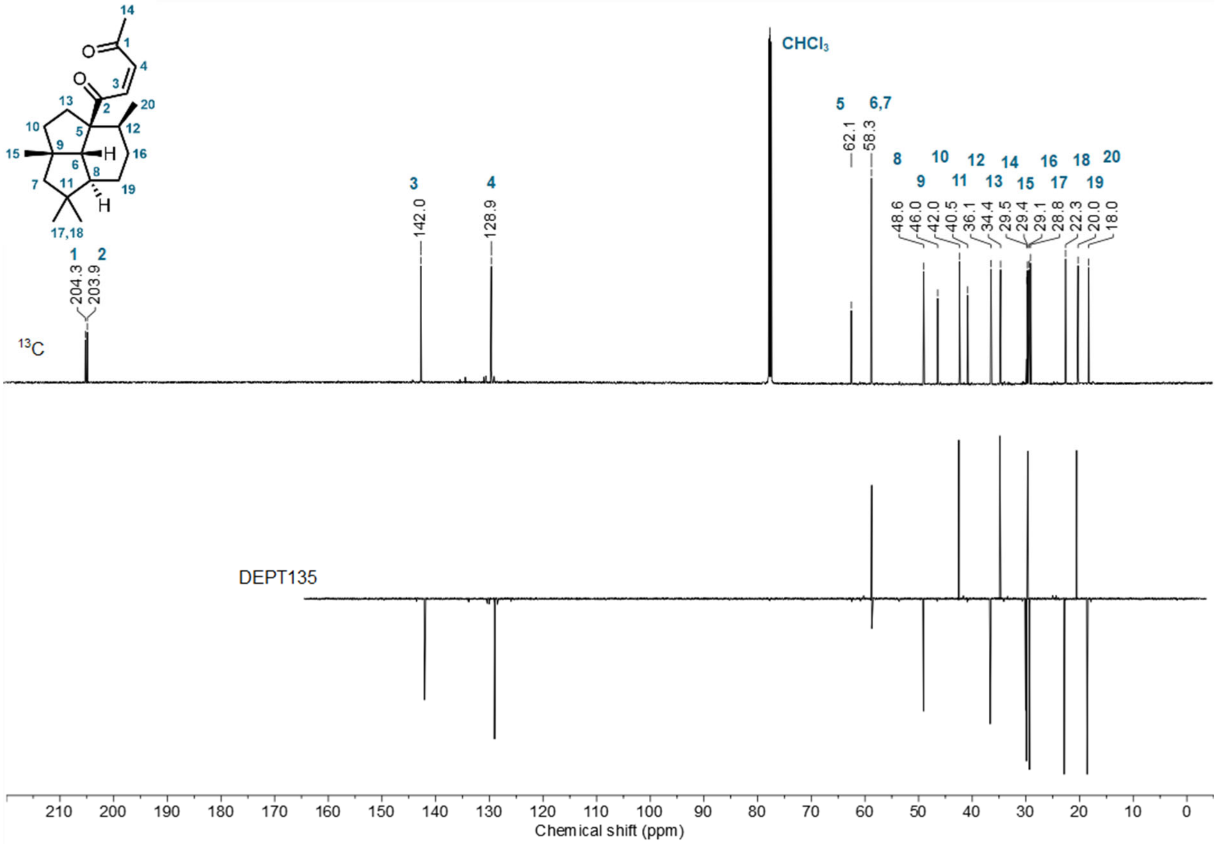

COSY (600 MHz, $CDCl_3$, 298K)

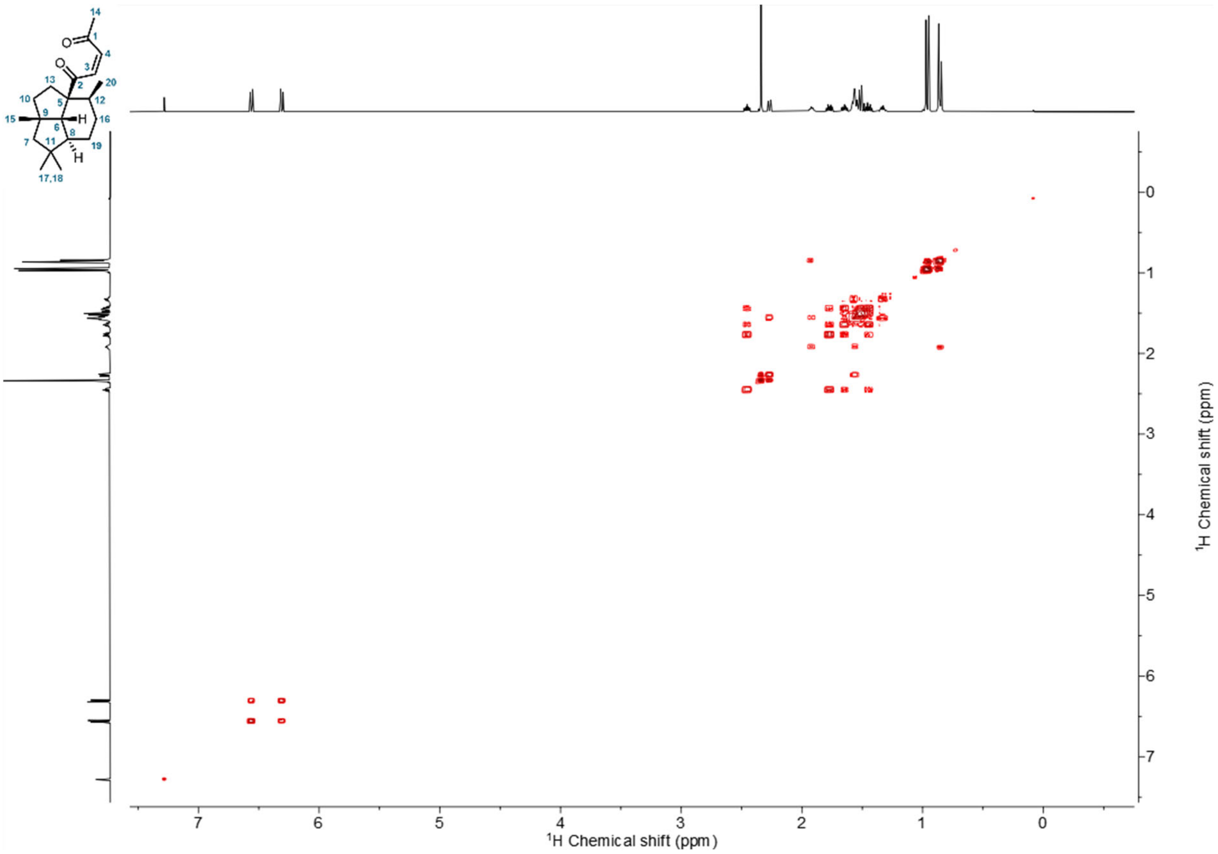


NOESY (600 MHz, $CDCl_3$, 298K)

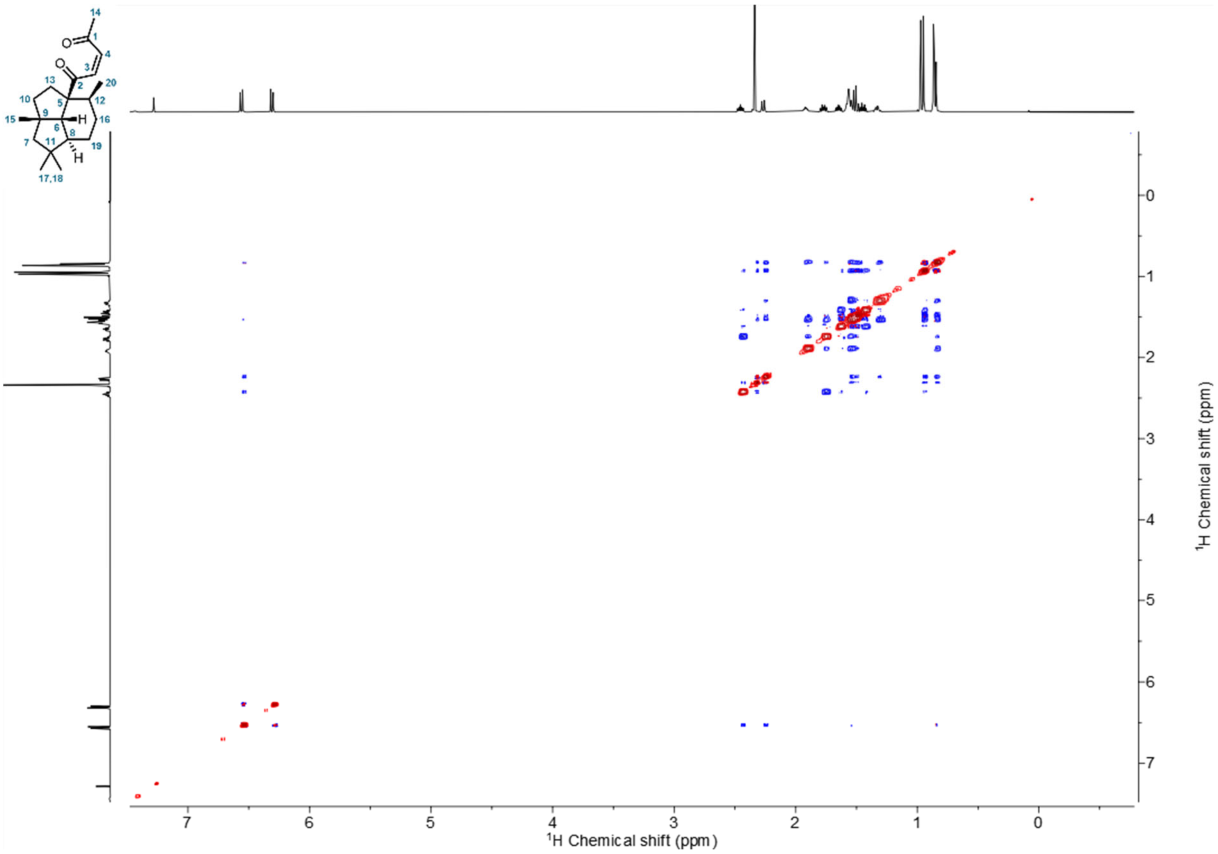

HSQC (600 MHz, $CDCl_3$, 298K)

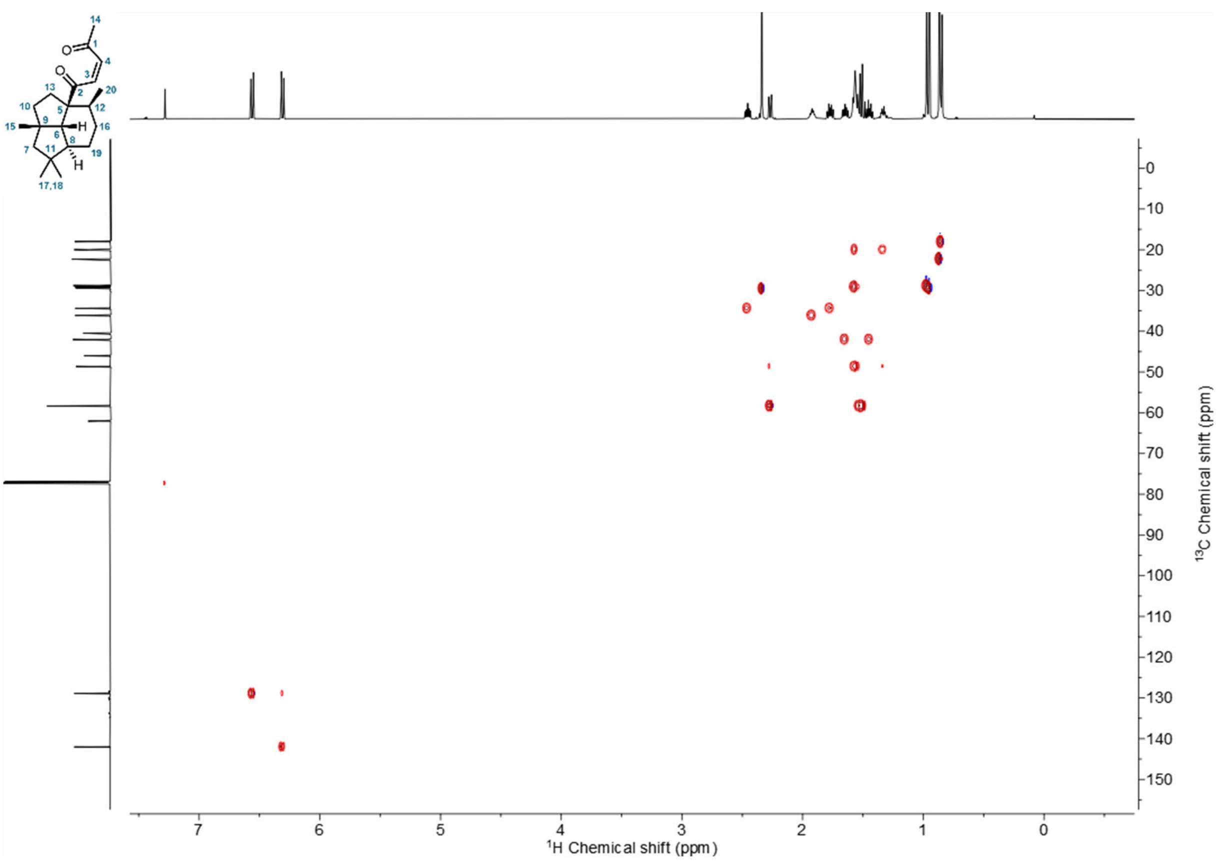


HMBC (600 MHz, $CDCl_3$, 298K)

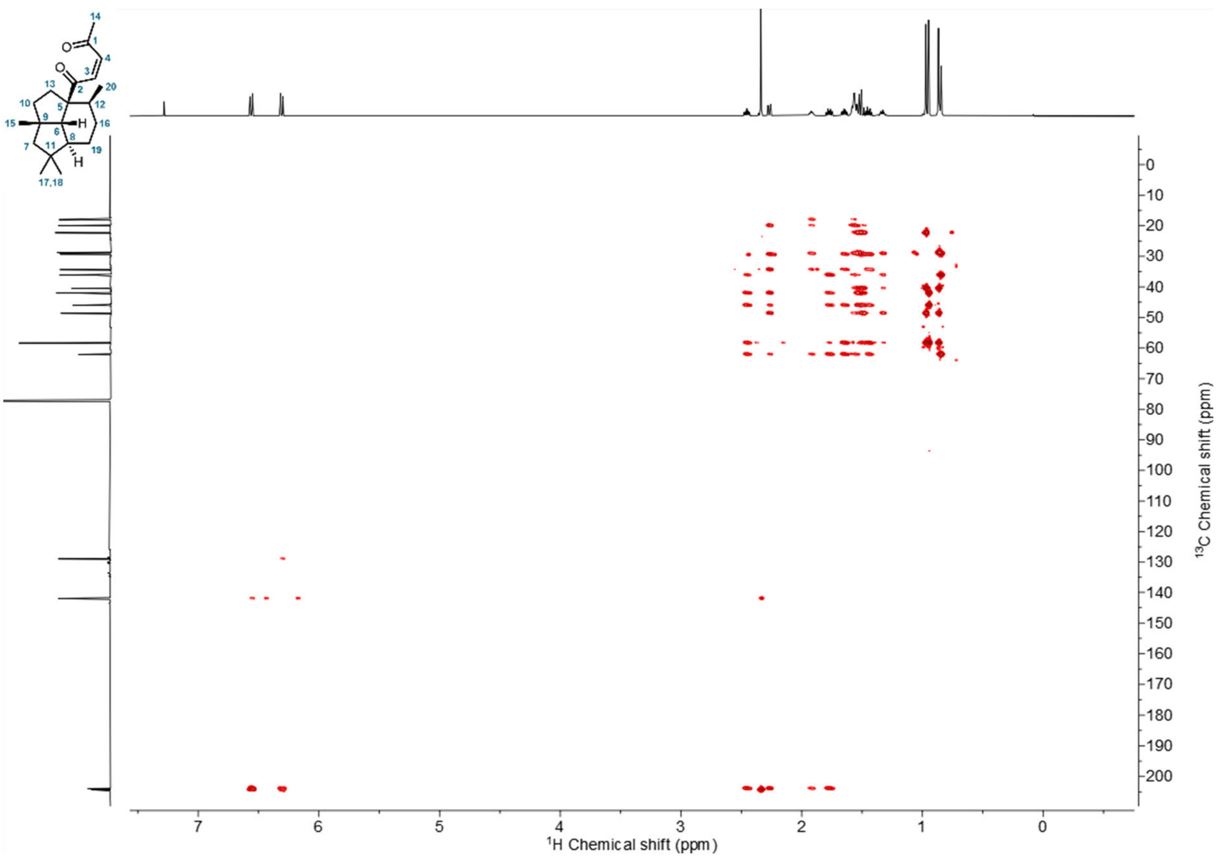

Supplement: Supplementary file 1 [file au5c00492_si_001.pdf]
